# Dose-dependent structural and electron-density features in the lytic polysaccharide monooxygenase *Nc*AA9D

**DOI:** 10.1107/S205979832600639X

**Published:** 2026-07-28

**Authors:** Samuel A. Miller, William B. O’Dell, Flora Meilleur

**Affiliations:** a Department of Molecular and Structural Biochemistry North Carolina State University 128 Polk Hall Raleigh NC27695 USA; b Neutron Scattering Division Oak Ridge National Laboratory PO Box 2008 Oak Ridge TN37831 USA; Institut de Biologie Structurale, France

**Keywords:** radiation damage, lytic polysaccharide monooxygenases, dioxygen binding, dioxygen species identification

## Abstract

Structural studies of copper-containing lytic polysaccharide monooxygenases (LPMOs) by X-ray crystallography are often complicated by radiation damage. In this study, we analyze a series of 36 X-ray crystal structures of *Nc*AA9D, a *Neurospora crassa* AA9-family LPMO, determined from data collected at cryogenic temperature from a single crystal to investigate the progressive effects of radiation damage at the active site of this enzyme. We report new insights into the dose-dependence of active-site geometry in LPMOs and utilize the unique pre-bound dioxygen site of *Nc*AA9D to analyze the impact of X-ray dose on the electron density of this species. It is well established that photoreduction of the LPMO active-site copper(II) leads to expulsion of its water ligands. We further characterize this displacement and the corresponding electron-density smearing, a phenomenon that can lead to the erroneous modeling of copper-bound dioxygen species. These findings suggest that radiation-dose series collected from a single crystal provide invaluable data to support unambiguous assignment of radiation-sensitive intermediates at the active site of LPMOs and other radiation-sensitive redox enzymes.

## Abstract 2

A series of 36 structures of the *Neurospora crassa* LPMO *Nc*AA9D were refined against X-ray crystal datasets collected with increasing dose from a single crystal to provide insights into dose-dependent structural changes at the active site of the enzyme. The findings underscore the importance of minimizing accumulated dose during cryo-X-ray crystallographic studies of lytic polysaccharide monooxygenases to prevent the misinterpretation of radiation damage-related electron-density features.

## 1. Introduction

Radiation damage to protein crystals is a well-established concern within the field of macromolecular X-ray crystallo­graphy, posing significant challenges for structural modeling and interpretation. Radiation damage to protein crystals is broadly split into two categories: global and specific. Global radiation damage manifests itself in reciprocal space, and is readily detected during the early steps of data processing, and is mitigated by truncating datasets to remove images exhibiting significant damage (Shelley & Garman, 2022 ▸). Conversely, specific radiation damage occurs at particular sites and can lead to metal-center and redox-cofactor reduction, disulfide-bond cleavage, and aspartate and glutamate decarboxylation (Garman & Weik, 2023 ▸). Specific radiation damage is not detectable until the model-building and refinement stage of data analysis, where its effects can lead to misinterpretation of a protein’s structural features and mechanisms of action (Owen *et al.*, 2006 ▸).

To address the challenges posed by specific radiation damage, dose-series studies have emerged as the gold standard for identifying radiation-induced structural changes (Shelley *et al.*, 2018 ▸). In this technique, X-ray dose is purposefully accumulated across multiple crystallographic datasets (Burmeister, 2000 ▸; Weik *et al.*, 2000 ▸; Bury *et al.*, 2015 ▸; Tandrup *et al.*, 2022 ▸; Rose *et al.*, 2024 ▸; Ebrahim *et al.*, 2019 ▸; de la Mora *et al.*, 2020 ▸), enabling scientists to correlate structural changes with X-ray doses calculated using software such as *RADDOSE*-3*D* (Bury *et al.*, 2018 ▸).

**Figure 1:**
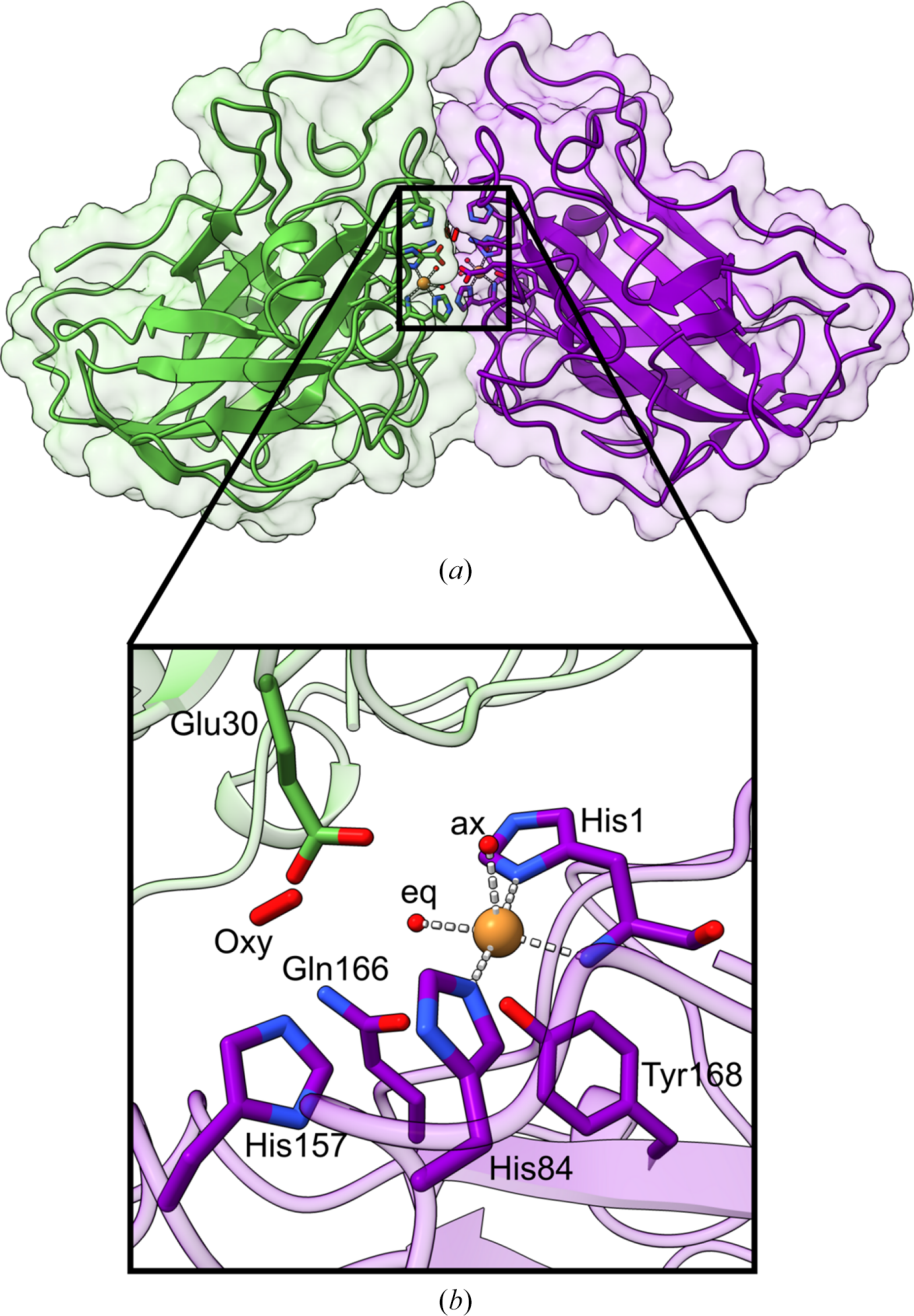
Representative structure of *Nc*AA9D (PDB entry 9z8o). (*a*) The two chains of *Nc*AA9D (chain *A* in purple and chain *B* in green) are arranged carbohydrate-binding face to carbohydrate-binding face, secluding the active sites from the bulk solvent and facilitating the formation of two pre-bound dioxygen sites. (*b*) The active and pre-bound dioxygen sites of chain *A* in *Nc*AA9D. In the Cu(II) state, the active-site copper is coordinated by the histidine brace and an axial and equatorial water. In *Nc*AA9D, a pre-bound dioxygen species is observable just past the equatorial water. It is surrounded by His157, Gln166, and Glu30 from the opposing chain. All visualizations of the protein structure and electron-density maps were created in *ChimeraX* (v1.8; Meng *et al.*, 2023 ▸).

Lytic polysaccharide monooxygenases (LPMOs) exemplify metalloproteins whose study is complicated by radiation damage. These monocopper enzymes are central to the industrial conversion of biomass into value-added products, and they have recently been implicated in a wide array of important biological functions, spanning pathogenesis, organism development, and symbiotic interactions (Sagarika *et al.*, 2022 ▸; Hansen *et al.*, 2023 ▸; Abraham *et al.*, 2016 ▸; Vandhana *et al.*, 2022 ▸). LPMOs contain a Cu atom coordinated by a ‘histidine brace’ motif, where the copper is bound in its equatorial plane by the N-terminal amino group, the N^δ^ atom of His1, and the N^ɛ^ atom of another conserved histidine. Positioned axially to the Cu atom is the hydroxyl group of a tyrosine residue, which is replaced by a phenylalanine in some AA10 LPMOs (Vaaje-Kolstad *et al.*, 2017 ▸). In the enzyme’s resting Cu(II) state the copper is also coordinated by two water molecules: one in the axial and one in the equatorial position (Fig. 1 ▸*b*).

Reduction of the active-site Cu(II) to Cu(I) is central to the LPMO catalytic mechanism. After this reduction, LPMOs activate dioxygen species to exhibit different activities (Rieder & Sørlie, 2023 ▸). The primary activity of LPMOs is their peroxygenase activity, where the activation of H_2_O_2_ leads to hydroxylation of a productively bound carbohydrate substrate at the C1 or C4 position, followed by spontaneous glycosidic bond breakage (Bissaro *et al.*, 2017 ▸). In their oxidase activity, LPMOs can also bind and activate O_2_ to drive the production of H_2_O_2_ (Munzone *et al.*, 2024 ▸). Despite general agreement on this overall mechanism, there are differing views as to whether the active-site copper binds dioxygen species at the axial or equatorial position. Earlier studies proposed axial dioxygen binding and activation based on X-ray crystallographic and computational studies (Li *et al.*, 2012 ▸; Solomon *et al.*, 2011 ▸; Kim *et al.*, 2014 ▸), whereas more recent studies have used computational and spectroscopic information to support the hypothesis of equatorial dioxygen activation (Kjaergaard *et al.*, 2014 ▸; Hedegård & Ryde, 2017 ▸; Wang *et al.*, 2019 ▸). Further supporting the latter hypothesis, we previously demonstrated dioxygen binding and activation at the equatorial position of *Nc*AA9D *via*X-ray and neutron crystallography (O’Dell, Swartz *et al.*, 2017 ▸; O’Dell *et al.*, 2017 ▸; Schröder *et al.*, 2021 ▸, 2022 ▸). However, resolving such details, especially modeling dioxygen species, remains complicated by radiation-damage effects at the active site during X-ray crystallographic studies.

Previous studies of radiation damage in LPMOs have demonstrated that excessive X-ray dose photoreduces the copper center from Cu(II) to Cu(I), causing the axial and equatorial waters to migrate away from the Cu atom (Gudmundsson *et al.*, 2014 ▸; Tandrup *et al.*, 2022 ▸). Tandrup *et al.* (2022 ▸) also proposed various active-site angles which could be diagnostic for active-site photoreduction, underscoring the need for dose-series studies to evaluate and identify features of radiation damage and copper photoreduction in LPMOs.

In our prior studies of a *Neurospora crassa* AA9 LPMO, *Nc*AA9D (UniProt Accession Q1K8B6), we identified crystal-packing features which facilitate active-site seclusion and pre-bound dioxygen formation (O’Dell, Agarwal & Meilleur, 2017 ▸). Here, we expand upon this work with a cryo-temperature dose-series study of *Nc*AA9D to probe dose-dependent changes in its active and pre-bound dioxygen sites. We collected 36 datasets at high resolution (1.10 Å) from a single crystal using a helical data-collection strategy and used them to construct 36 wedge datasets with similar dose and 36 pseudohelix datasets with increasing dose. We utilize the large number of datasets to leverage statistical analysis of the structures’ characteristics to highlight several features which correlate with X-ray dose accumulation and extend our current knowledge of radiation damage in LPMOs.

## 2. Methods

### 2.1. Protein expression and purification

*Nc*AA9D was expressed in *Komagataella phaffii* (previously *Pichia pastoris*) as previously described in detail by O’Dell, Swartz *et al.* (2017 ▸). A pPICZαA expression vector containing the bleomycin resistance gene and the coding sequence of *Nc*AA9D was transformed into *K. phaffii* SuperMan_5_(HIS^+^) (BioGrammatics). This strain was chosen because it reduces the mannosylation of N-linked glycans, which improves the diffraction quality of *Nc*AA9D crystals (O’Dell, Swartz *et al.*, 2017 ▸; Jacobs *et al.*, 2009 ▸). The resulting transformants were selected for bleomycin resistance using Zeocin (Thermo Fisher Scientific) and screened for methanol-utilization phenotype. The positive colonies were used to express *Nc*AA9D. Protein expression was performed using a fed-batch approach at pH 5.2 in a bioreactor system, using fermentation basal salts medium, fermentation trace metals solution, glycerol, and Zeocin. The bioreactor was inoculated with overnight culture, and the inoculum was grown until it reached an OD_600_ of 110.0. Methanol was then added to induce the production of *Nc*AA9D for 48 h at 25°C.

The procedure for *Nc*AA9D purification was also described by O’Dell, Swartz *et al.* (2017 ▸). *Nc*AA9D was secreted into the culture medium, so the enzyme was harvested by centrifuging the culture and keeping the supernatant. *Nc*AA9D was purified from the supernatant by hydrophobic interaction chromatography, anion-exchange chromatography, and size-exclusion chromatography.

### 2.2. Protein crystallization

*Nc*AA9D crystals were prepared as described by O’Dell, Swartz *et al.* (2017 ▸), where long crystals were obtained by microseeding. To prepare a crystal microseed stock, hanging drops were prepared by mixing 1 µl *Nc*AA9D (12.5 mg ml^−1^ in 20 m*M* sodium acetate pH 5.0) and 1 µl reservoir solution [22%(*w*/*v*) PEG 3350, 100 m*M* HEPES free acid pH 6.0]. The drops were equilibrated against 1 ml reservoir solution. Plate-like crystals grew within a week, and the crystals were crushed using PTFE Seed Beads (Hampton Research) according to the manufacturer’s recommendations.

To grow large crystals of *Nc*AA9D suitable for this experiment, a sitting drop was prepared with 66 µl *Nc*AA9D, 129 µl 25% PEG 3350, 100 m*M* HEPES free acid pH 6.0, and 3 µl diluted *Nc*AA9D microseed stock. The large drops were equilibrated against 50 ml reservoir solution at 18 °C. Multiple elongated cuboid crystals of *Nc*AA9D grew in the drop.

### 2.3. Data collection

X-ray diffraction data were collected on the Southeast Regional Collaborative Access Team beamline 22-ID at the Advanced Photon Source. The unattenuated beam had a size of 40 × 30 µm (FWHM, rectangular Gaussian profile). The beam’s energy, flux, and dimensions at the crystal position were set to 12.4 keV, 0.49 × 10^12^ photons s^−1^, and 20 × 20 µm, respectively. All diffraction data were collected on a MAR 300HS high-speed CCD detector, with a crystal-to-detector distance of 108.00 mm.

**Figure 2:**
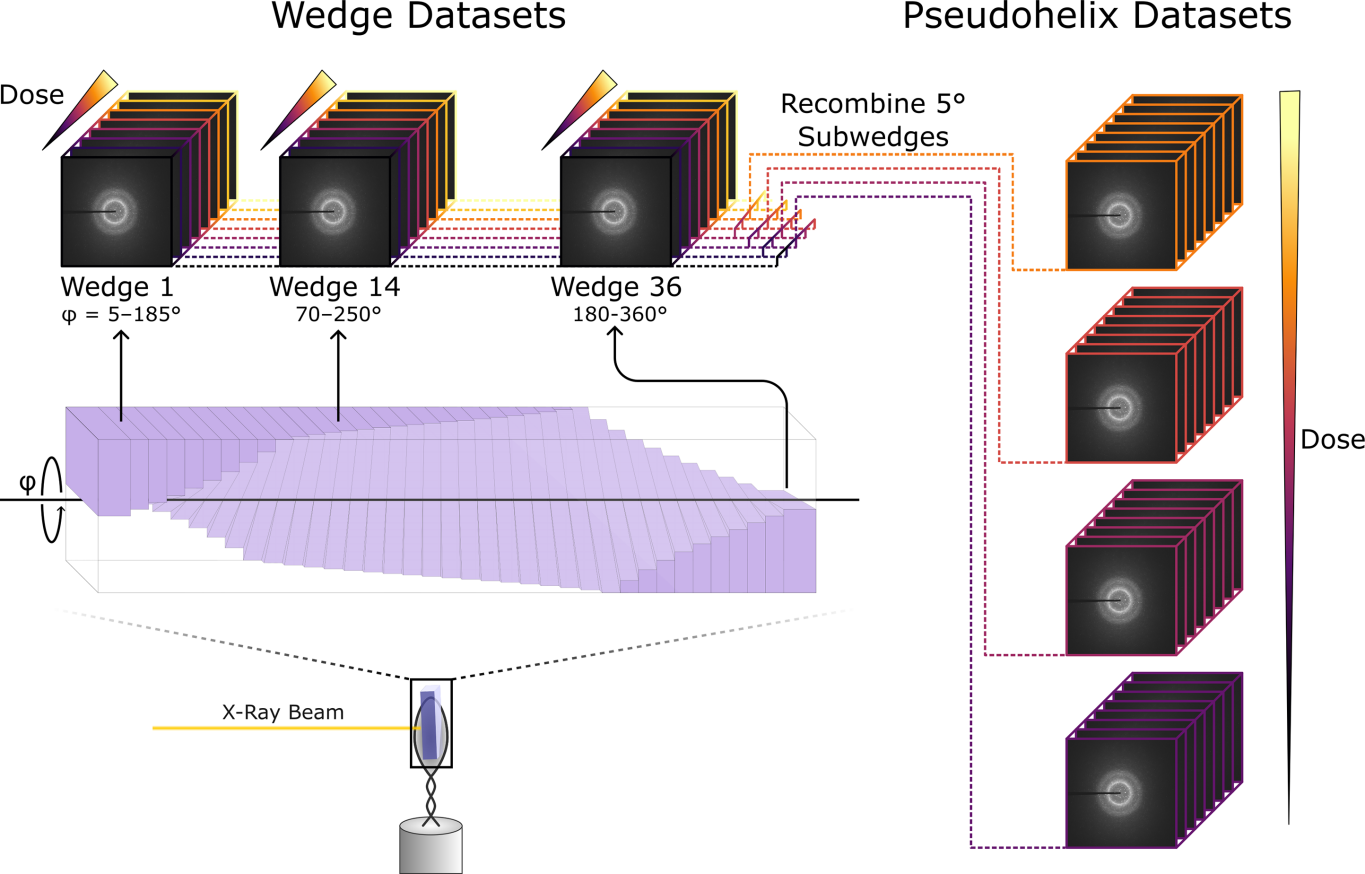
Schematic of the data-collection procedure. 38 wedge datasets, each consisting of 180° of data, were collected, incrementing the starting φ angle by 5° between each wedge. Dose progressively accumulates during wedge collection, so later frames inherently represent a higher dose. After excluding the two wedge datasets from each end of the crystal, the 36 remaining were split into 5° subwedges, and these were recombined to generate 36 dose-representative pseudohelix datasets.

A crystal of *Nc*AA9D (approximately 250 × 1200 × 250 µm) was aligned with its long axis perpendicular to the beam path (parallel to the goniometer’s φ rotation axis). A total of 38 wedge datasets were collected from the crystal at 100 K. Each dataset comprised a 180° wedge (180 frames; 1° φ oscillation; 1 s exposure). Each wedge was spaced from the others by a 30 µm translation along the long axis of the crystal. In addition, the start φ angle of each wedge was incremented 5° relative to the previous dataset (Fig. 2 ▸). For example, the first wedge dataset was collected from 0° to 180°, the second from 5° to 185°, and so on.

### 2.4. Data processing

#### 2.4.1. Identification of potential dataset recombinations

Thirty-eight 180° wedge datasets were obtained during data collection. The wedge datasets at both ends of the crystal were discarded to mitigate any potential inaccuracies arising from their position near the crystal’s edge. For simplicity, these 36 internal wedge datasets will be referred to as wedges 1–36.

Because the starting φ angles of each of the wedge datasets were increased in 5° increments, additional datasets may be constructed by combining 5° ‘subwedges’ from each of the 36 wedge datasets. We refer to these recombined datasets as ‘pseudohelix’ datasets because of their geometrical similarity to true helical data collection but lack of translation over 5° subwedges. Considering all the subwedge combinations as a sliding window, there are a total of 176 possible pseudohelix datasets, and each pseudohelix represents a snapshot in a series of increasing doses. For example, the lowest dose pseudohelix dataset may be constructed by combining the first 5° subwedge from each wedge. The resulting pseudohelix dataset represents the same angular range as wedge 1 (φ = 5–185°) but has a lower dose because all its frames are from earlier in data collection. The second pseudohelix would represent the angular range φ = 6–186°, and so on.

#### 2.4.2. Diffraction decay-weighted dose calculation

The data-collection strategy used for this study likely resulted in dose-dependent hole burning at the center of the rotation axis (Warkentin *et al.*, 2017 ▸; de la Mora *et al.*, 2020 ▸). To more accurately reflect the functional dose of each dataset, the average diffraction decay-weighted dose (DDWD) was calculated for the possible wedge and pseudohelix datasets using the procedure described previously by Dickerson *et al.* (2024 ▸).

First, using *DIALS* (v3.26), unscaled and unmerged datasets were generated at 1.1 Å resolution (largest inscribed circle) for the first 25 possible pseudohelix datasets, thus representing a representative dose range while avoiding datasets where intensity decay may be too severe (Winter *et al.*, 2018 ▸). The Wilson *B* factor and Wilson scale factor were estimated for each dataset using *WILSON* in the *CCP*4 suite (Agirre *et al.*, 2023 ▸).

Next, the Wilson *B* factors and scale factors *K* (the reciprocal of the Wilson scale factor) were plotted against the average fluence-weighted dose (FWD) estimated by *RADDOSE*-3*D* (Bury *et al.*, 2018 ▸). The parameters γ, β, and *B*_0_ of the Leal intensity-decay model were then estimated using least-squares regression (Leal *et al.*, 2013 ▸). The Wilson *B* factors plotted against average FWD were used to estimate β and *B*_0_, and the scale factors *K* plotted against average FWD were used to estimate γ. For both regressions, points were excluded if they lay outside the region of the plot showing the expected trend (Supplementary Fig. S1). To estimate γ, non­linear least squares was used with the Gauss–Newton method (Bates & Watts, 1988 ▸). The estimated parameters were used as an input to *RADDOSE*-3*D*, along with the experimental geometry, to calculate an average DDWD for every possible pseudohelix and wedge dataset (Dickerson *et al.*, 2024 ▸).

*ParaView* (v6.0.1; Ayachit, 2015 ▸) was used to visualize dose distribution in the protein crystal, using the dose-state output file from *RADDOSE*-3*D*.

To assist in visualizing the complex dose distribution generated by our data-collection strategy, a fluence- and diffraction decay-weighted dose distribution was calculated for each pseudohelix. *RADDOSE*-3*D* provides CSV files containing the dose and fluence of each voxel for each ‘image’ it calculates. Using the procedure outlined by Dickerson *et al.* (2024 ▸) and implemented in the *RADDOSE*-3*D* source code, the contribution of each voxel to a given dataset was estimated. Each voxel’s normalized contribution was then paired with its corresponding dose to generate an effective dose distribution for the dataset: an estimate of which doses most strongly contribute to a dataset.

#### 2.4.3. Sampling

Of the 176 potential pseudohelix datasets, 36 were selected for structure determination using hybrid deterministic and weighted random sampling. First, every 25th dataset was selected by deterministic sampling (eight datasets in total), leaving 168 remaining pseudohelices. From these, 28 datasets were selected by weighted random sampling, where the weights were the increase in average DDWD from the previous dataset. This mixture of deterministic and weighted random sampling ensures that the selected sample set is representative of the possible range of DDWDs and start angles obtained during the experiment.

#### 2.4.4. Data indexing, integration, and merging

X-ray diffraction data were indexed, integrated, and merged in *DIALS* (v3.26; Winter *et al.*, 2018 ▸). All of the datasets were processed to 1.1 Å resolution, as dictated by the largest inscribed circle on the detector. The 36 wedge datasets were processed as is, without any additional modifications. Additional steps were employed to construct the pseudohelix datasets sampled in Section 2.4.3.

To construct the 36 sampled pseudohelices, five-frame ‘subwedges’ were extracted from each wedge dataset and assembled into dose-representative pseudohelix datasets. Each pseudohelix contains 180° of data, with 5° contributed by each wedge. This approach is similar to that used in small-wedge serial crystallography, except that we used multiple small wedges collected from a single crystal, rather than multiple crystals as described in such studies (Cherezov *et al.*, 2007 ▸; Rasmussen *et al.*, 2011 ▸; Chaussavoine *et al.*, 2022 ▸). Statistics for each wedge and pseudohelix dataset are listed in Supplementary Tables S1–S12.

#### 2.4.5. Structure refinement

Phases were obtained from the previously published structure of *Nc*AA9D in the Cu(II) resting state (PDB entry 5tkg; O’Dell, Agarwal & Meilleur, 2017 ▸). We refined this input model against the X-ray data from pseudohelix 1 by alternating between refinement with *phenix.refine* (v2.0-5936) and manual model building/refinement with *Coot* (v1.1.18) (Liebschner *et al.*, 2019 ▸; Emsley *et al.*, 2010 ▸). The resulting pseudohelix 1 model served as the starting model for refinement of all other structures against their respective datasets (both wedges and pseudohelices). This was performed with ten macrocycles of rigid-body, *XYZ*, *B*-factor, and occupancy refinement in *phenix.refine* (v1.21.2-5419). To ensure the integrity of dose-dependent structural changes, the *B* factors of atoms with a partial occupancy were held fixed during refinement. This approach allowed either the occupancy or *B* factor of an atom to act as the sole indicator of local electron-density changes, minimizing the risk of overlooking dose-related structural variations. Model-refinement statistics for each wedge and pseudohelix dataset are listed in Supplementary Tables S1–S12.

### 2.5. Data analysis

#### 2.5.1. Principal component analysis

Principal component analysis (PCA) was used to distinguish potentially distinct clusters of wedge and pseudohelix datasets. First, the hydrogens were removed from each structure, and the *B* factors, occupancies and *XYZ* coordinates of the remaining atoms were compiled into two matrices: one for the wedge structures and another for the pseudohelix structures. In the matrices, each row represented a structure and each column represented the value of an atomic parameter. Each column of the matrices was centered and scaled to represent *Z*-scores.

Following this, PCA was performed on the centered and scaled matrices using singular value decomposition. Using the *R* package *PCDimension* (v1.1.14), the broken stick method was used to determine the optimal number of dimensions to keep (Supplementary Fig. S6; Wang & Coombes, 2025 ▸; Jackson, 1993 ▸). Two dimensions were kept for the wedge structures, and three were kept for the pseudohelix structures. Following this, *k*-means clustering was conducted to identify distinct clusters of structures. The *R* package *NbClust* (v3.0.1) was utilized to determine the relevant number of clusters and conduct the clustering (Charrad *et al.*, 2014 ▸, 2022 ▸). The *R* packages *factoextra* (v2.0.0) and *plotly* (4.12.0) were used to assist in visualizing the results of PCA and *k*-means clustering (Kassambara *et al.*, 2026 ▸; Sievert, 2019 ▸; Sievert *et al.*, 2026 ▸).

To compare the similarity among the wedge and pseudohelix datasets, root-mean-square deviations (RMSDs) were calculated between every pair of models for atomic position, *B* factor, and occupancy.

#### 2.5.2. Structural parameter analysis

The structures were analyzed in the *R* Statistical Software (v4.5.2) using the *bio*3*d* package (v2.4.5; R Core Team, 2025 ▸; Grant *et al.*, 2021 ▸, 2024 ▸). The *R* packages *tidyverse* (v2.0.0), *janitor* (2.2.1), *vroom* (v1.7.1), and *fst* (v0.9.8) were used to assist in data import, cleaning, and wrangling, and the *R* packages *ggplot*2 (v4.0.3), *systemfonts* (v1.3.2), *cowplot* (v1.2.0), *ggnewscale* (v0.5.2), and *ggforce* (v0.5.0) were used to generate two-dimensional data visualizations (Wickham *et al.*, 2019 ▸, 2026 ▸; Wickham, 2016 ▸, 2023 ▸; Firke *et al.*, 2024 ▸; Hester *et al.*, 2026 ▸; Klik, 2022 ▸; Pedersen *et al.*, 2026 ▸; Wilke, 2025 ▸; Campitelli, 2025 ▸; Pedersen, 2025 ▸). The scripts used for statistical analysis and plot generation are publicly available at https://github.com/sadamill/NcAA9D_DoseSeries.

Multiple measurements were recorded for both chains of all the pseudohelix datasets. Occupancies of the following atoms were recorded: H_2_O_ax,in_, H_2_O_eq,in_, dioxygen, intact Glu30 in chain *B*, and CO_2_ in chain *B*. These atoms were monitored because of their proximity to the *Nc*AA9D active and pre-bound dioxygen sites and their dose-responsive nature. We only track decarboxylation and its associated CO_2_ in chain *B* (associated with pre-bound and active site *A*) since there was no observable decarboxylation of Glu30 in chain *A* (associated with pre-bound and active site *B*). We also recorded the active-site geometry by measuring various angles and distances. The active-site angles θ_1_, θ_2_, θ_3_, θ_H–H_, θ_T_, θ_H1_, and θ_HN_ were calculated as defined previously (Vu & Ngo, 2018 ▸; Tandrup *et al.*, 2022 ▸). Finally, we measured distances between the active-site copper and H_2_O_ax,in_, H_2_O_eq,in_, N_term_, His1 N^δ^, His84 N^ɛ^, and Tyr168 O^η^.

To compare the measured angles, distances, and occupancies across structures, we performed multiple linear regression of the target characteristic against DDWD and chain ID. These regressions resulted in a model describing the dose- and chain-responsiveness of a given characteristic, with the following equation:
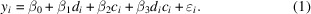
Here, *y_i_* represents an observed value of the characteristic at a given DDWD *d_i_* and in a given chain *c_i_*. β_0_ represents the *y* intercept, β_1_ describes the dose response of the characteristic, β_2_ describes the effect of the chain on the characteristic, and β_3_ describes whether the chains exhibit distinct dose sensitivities. In this experiment, the chain ID was treated as a factor with two levels, *A* and *B*, to which *R* assigned the indicator values 0 and 1 for the linear regression. Each observed data point is different from the model prediction by its residual, ɛ*_i_*. Because the data may exhibit nonconstant variance between chains, generalized least squares was utilized with
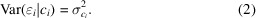
This allows the residual term to vary depending on the chain, as opposed to ordinary least squares, which assumes homoscedasticity. Generalized least squares was conducted with the *R* package *nlme* (v3.1.169; Pinheiro *et al.*, 2025 ▸).

We used the *R* package *emmeans* (v2.0.2) to estimate the dose responsiveness of the characteristic in each chain and the contrast between the calculated slopes (Lenth *et al.*, 2026 ▸). These slopes and contrasts were recorded, as well as additional statistical information (Supplementary Tables S13–S15). The *p*-value threshold of 0.05 was used to determine whether the calculated slopes and their respective contrasts were statistically significant.

We excluded the wedge structures from linear regression analysis. Unlike the pseudohelix series, the wedge series was not designed to represent a monotonic progression due to a single explanatory variable, and there was no *a priori* physical basis to expect a linear position-dependent response in the measured parameters. We therefore characterized wedge heterogeneity using unsupervised methods, PCA and *k*-means clustering (see Section 2.5.1), which capture multivariate structure and do not assume a linear, monotonic trend.

#### 2.5.3. Electron-density analysis

To identify regions of dose-dependent electron-density change, an isomorphous difference map between the highest and lowest dose pseudohelices (Δ*F*_dose_) was calculated with *phenix.fobs_minus_fobs_map* in the *Phenix* software suite (Liebschner *et al.*, 2019 ▸). An isomorphous difference map between wedge 36 and wedge 1 (Δ*F*_wedge_) was also calculated to act as a negative control for dose response, since these datasets have nearly identical DDWDs (5.45 and 5.48 MGy, respectively). Phases were retrieved from the lowest dose pseudohelix and wedge 1 for calculation of the Δ*F*_dose_ and Δ*F*_wedge_ maps, respectively.

## 3. Results and discussion

### 3.1. Generation of dose-representative *Nc*AA9D structures

For this study, we used a single elongated cuboid crystal of *Nc*AA9D (approximately 250 × 1200 × 250 µm). Because of the crystal’s unique dimensions, we were able to collect 38 separate wedge datasets from the same crystal, each containing 180 frames and starting at a φ angle increased by 5° from the previous wedge’s starting φ angle. We discarded the wedge datasets from the far ends of the crystal to avoid any potential edge-related artifacts. With the remaining 36 wedges, up to 176 pseudohelix datasets could be constructed by combining 5° ‘subwedges’ from each of the wedge datasets (Fig. 2 ▸). The wedges sample different regions of the crystal along the long axis and all represent a similar dose (Fig. 2 ▸). The pseudohelices, by contrast, share similar angular coverage to the wedges while spanning a range of doses and all regions of the crystal. For example, the first of the possible pseudohelices may be constructed by combining the first 5° of data from each wedge. This pseudohelix represents the same angular range as wedge 1 (φ = 5–185°) but has a significantly lower dose and represents a position-averaged dataset derived in part from all 36 wedges.

### 3.2. Analysis of dose accumulation

**Figure 3:**
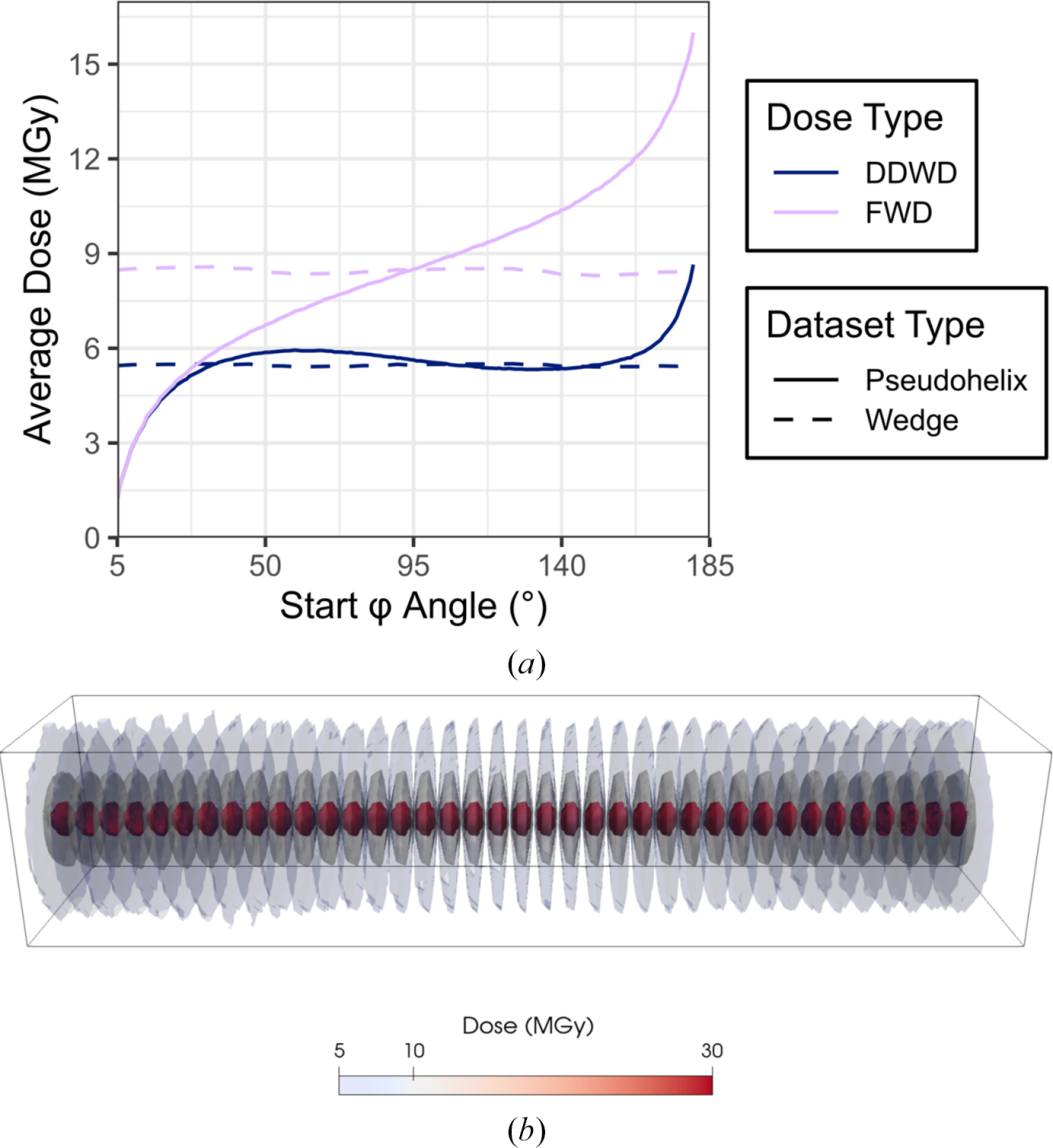
*RADDOSE*-3*D* results. (*a*) Average DDWDs (navy) and FWDs (lavender) estimated for all the wedge (dashed) and pseudohelix (solid) datasets. It is evident that the pseudohelix datasets progressively accumulate dose. The diffraction decay weighting is evident from the slower DDWD accumulation. (*b*) Distribution of dose in the crystal after the collection of 38 wedge datasets. Each collection segment contains a very high-dose region centered around the rotation axis, which exceeds the Garman limit of 30 MGy. The wedges were spaced apart far enough to prevent significant accumulation of dose in neighboring wedges. Dose isosurfaces are drawn at 5 MGy (light blue), 10 MGy (blue) and 30 MGy (red). All graphs were created using the *ggplot*2 package (v4.0.3) and (*b*) was made using *ParaView* (v6.0.1; Wickham *et al.*, 2026 ▸; Ayachit, 2015 ▸).

Despite the wedge datasets being collected from the same crystal, the results from *RADDOSE*-3*D* indicate that their spacing was sufficient to prevent dose from ‘bleeding over’ into subsequent wedges, thus preventing accumulation of dose from wedge to wedge (Fig. 3 ▸*a*). These results are further supported by the visible formation of a striped pattern along the crystal’s long axis, qualitatively consistent with irradiation at discrete translation steps (Supplementary Fig. S2). This striping is likely due to changes in the refractive index at irradiated positions and has been reported in other radiation-damage studies (Warkentin *et al.*, 2017 ▸). Our results align with previous studies of photoelectron escape, which conclude that damage transferred outside the beam footprint is negligible with beams larger than 15 µm and at X-ray energies near 12 keV (Nave & Hill, 2005 ▸; Sanishvili *et al.*, 2011 ▸). We used a 20 × 20 µm beam with an energy of 12.4 keV, putting our beam parameters well within these specifications.

The data-collection strategy used in this study likely causes dose-dependent hole burning at the center of the φ rotation axis (Warkentin *et al.*, 2017 ▸; de la Mora *et al.*, 2020 ▸). During data collection, the crystal volume at (or near) the intercept of the beam and φ rotation axis remained within the beam for the full duration of the wedge (180 frames × 1 s), whereas off-axis volumes moved into and out of the beam during rotation. Visualization of the crystal’s dose distribution reveals a high-dose region (≥30 MGy) at the center of each collection site (Fig. 3 ▸*b*). These crystal volumes exceed the Garman limit of 30 MGy: the dose at which the average experimental intensity is approximately 30% lower (Owen *et al.*, 2006 ▸). Based on the faster decay of high-resolution intensities, a resolution-dependent dose limit of 10 MGy Å^−1^ has been proposed, which would make the dose limit of this *Nc*AA9D crystal approximately 11 MGy (Howells *et al.*, 2009 ▸; Garman & Weik, 2017 ▸). Taken together, the above-described high-dose regions are expected to contribute less to the overall diffraction pattern.

Considering the highly damaged, low diffraction-efficiency regions, the average fluence-weighted dose (FWD) would overestimate the effective dose represented by the datasets. Rather, we used *RADDOSE*-3*D* to calculate the average diffraction decay-weighted dose (DDWD) of each dataset, which downweighs more damaged crystal volumes (Bury *et al.*, 2018 ▸; Dickerson *et al.*, 2024 ▸). The average DDWD provides a better measure of dose for this study because it accounts for the effects of global radiation damage, thus providing a more accurate estimate of the extent of site-specific radiation damage in our datasets. As expected, the wedge datasets all represent similar average DDWDs, ranging from 5.41 to 5.51 MGy. On the other hand, the pseudohelix datasets represent a wider range of average DDWDs, ranging from 1.22 to 8.65 MGy (Fig. 3 ▸*a*).

The average DDWD of each dataset is substantially lower than its respective FWD. The difference between FWD and DDWD is exemplified by the plateau and slight decrease in DDWD past the 45th potential pseudohelix (Fig. 3 ▸*a*): at this point, intensity decay at the high-dose crystal volumes becomes severe enough that it outpaces their dose accumulation. These results from *RADDOSE*-3*D* support our use of DDWD and demonstrate the utility of this metric for our study. They also indicate that comparisons of dose values calculated with different dose metrics are generally incompatible.

The accumulation of DDWD across the pseudohelix datasets is nonlinear. This is likely due to the data-collection strategy, where regions of the crystal closer to the φ rotation axis experience repeated irradiation and accumulate dose more continuously. We consider the accumulation of dose across the pseudohelices to occur in three general phases. (i) At first, dose rapidly accumulates in crystal regions closer to the φ rotation axis, causing a rapid initial increase in dose. (ii) Intensity decay at high-dose crystal volumes becomes severe enough that it outpaces their dose accumulation, causing a plateau and slight decrease in average DDWD past the 45th pseudohelix. (iii) As the crystal nears 180°, previously exposed crystal regions begin to accumulate more dose, causing a rapid increase in average DDWD in the later pseudohelices.

This pattern of dose accumulation is demonstrated by the evolution of fluence- and intensity decay-weighted dose distributions across the pseudohelices. In this series of distributions, a small number of crystal regions quickly ‘run off’ from the rest of the distribution as they accumulate dose more rapidly (Supplementary Fig. S3 and Movie S1). Incidentally, the ‘run-off’ region results in the formation of a right-skewed and bimodal dose distribution in the mid-dose range, causing the average DDWD to be pulled to higher values. This effect is mitigated, but not prevented, by diffraction decay-weighting. Complex spatial dose distributions are known to complicate dose modeling (Atakisi *et al.*, 2019 ▸), and most modern radiation-damage studies aim to illuminate the whole crystal volume with a top-hat beam profile or use small rotations to prevent this effect (Koulas *et al.*, 2026 ▸; Bourenkov *et al.*, 2026 ▸).

Taken together, our data-collection strategy prevented any dose from being carried over from wedge to wedge. We observe dose-dependent hole-burning at the center of the φ rotation axis, which was accounted for by calculating the average DDWD for every dataset. We explore the accumulation of dose within the crystal across the pseudohelices, finding that the increase in DDWD is nonlinear, mainly due to the experimental geometry. We further observe that the accumulation of dose across the crystal is uneven, creating a strongly skewed and bimodal dose distribution.

### 3.3. Dataset sampling, processing, and structure determination

**Figure 4:**
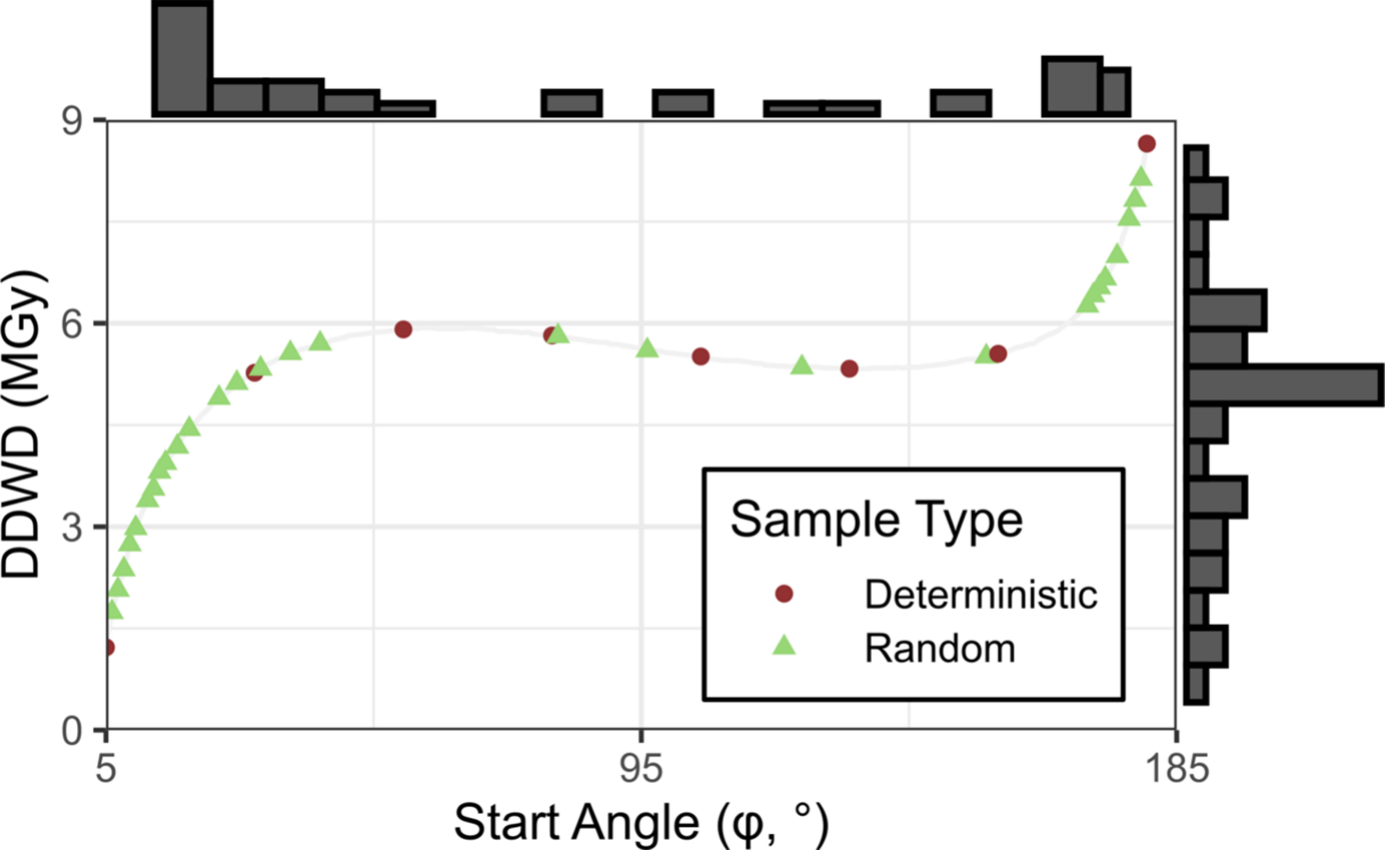
Sampling from the 176 possible pseudohelix datasets (gray line) by deterministic (maroon circles) and weighted random (green triangles) sampling. Every 25th dataset was manually selected to ensure a more even sampling of start angles. The remaining datasets were selected by weighted random sampling, so that datasets in regions of more rapid dose accumulation were more likely to be chosen. The result is a sample set which is representative of all the possible DDWDs and angular ranges, as shown by the marginal histograms.

Although there were 176 potential pseudohelix datasets, only 36 of these were selected for processing and structure determination. This was performed with hybrid deterministic and weighted random sampling. The sampling was conducted in such a way that the selected pseudohelix datasets provide coverage across the full spectrum of possible DDWDs and start φ angles (Fig. 4 ▸). After this, the 36 selected pseudohelix datasets and the 36 wedge datasets were indexed, integrated, scaled and merged using *DIALS* (Winter *et al.*, 2018 ▸). The lowest dose pseudohelix dataset was used to build and refine the pseudohelix 1 model, which was then used to refine all of the structures against their respective datasets using an identical refinement protocol, without manual model rebuilding.

### 3.4. Overview of *Nc*AA9D structures

All of the wedge and pseudohelix *Nc*AA9D structures were refined at 1.10 Å resolution (Supplementary Tables S1–S12). Although some small periodic dose- and position-specific trends are observed in the refinement statistics, none of these significantly outlie any of the others, indicating that the refinement procedure we used is robust. Of note, we observe a progressive increase in the Wilson *B* factor and average *B* factor of the pseudohelix datasets, consistent with global radiation damage (Supplementary Fig. S4). The 72 structures determined are very similar to each other: the maximum all-atom RMSD between any two structures is 0.250 Å (between wedges 18 and 35; Supplementary Fig. S5).

Overall, the structures are similar to those previously reported for *Nc*AA9D in the Cu(II) resting state. The structures exhibit a fibronectin-like/immunoglobulin-like β-sandwich core typical of LPMOs, and all of them have a planar carbohydrate-binding surface which contains the enzyme’s monocopper active site (Vaaje-Kolstad *et al.*, 2017 ▸; Bissaro *et al.*, 2018 ▸). Like other reports of *Nc*AA9D, the protein crystallized in space group *P*2_1_, with two noncrystallographic symmetry (NCS)-related molecules per asymmetric unit (Li *et al.*, 2012 ▸; O’Dell, Agarwal & Meilleur, 2017 ▸; Schröder *et al.*, 2022 ▸). The carbohydrate-binding surfaces of the two NCS-related molecules are packed against each other, effectively sequestering their normally solvent-exposed active sites (Fig. 1 ▸*a*).

The active sites of both chains consist of a Cu atom coordinated by the N-terminus (N_term_), the N^δ^ atom of His1 (His1 N^δ^), and the N^ɛ^ atom of His84 (His84 N^ɛ^), commonly called the histidine-brace motif. The hydroxyl group of Tyr168 (Tyr168 O^η^) is located at the axial position of the copper (Fig. 1 ▸*b*). Waters occupy the axial (H_2_O_ax_) and equatorial (H_2_O_eq_) positions in the enzyme’s resting Cu(II) state. The sequestered nature of the binding faces and active site of *Nc*AA9D also allows the observation of a ‘pre-bound’ dioxygen species positioned adjacent to the equatorial copper coordination site (Fig. 1 ▸*b*). Surrounding the pre-bound oxygen are the highly conserved residues His157 and Gln166, as well as Glu30 from the opposing chain (O’Dell, Agarwal & Meilleur, 2017 ▸; Schröder *et al.*, 2022 ▸).

### 3.5. Decoupling crystal heterogeneity- and dose-dependent effects

Our data-collection and resampling strategy was designed to decouple dose-dependent effects (pseudohelices) from crystal position-dependent effects (wedges). This is because each wedge model represents a different region of the crystal at a similar X-ray dose, while each pseudohelix model represents the average structure across the whole crystal at different X-ray doses. Accordingly, variation across wedges should primarily report crystal heterogeneity at an approximately constant dose, while variation across pseudohelices should primarily report dose dependence after averaging over crystal position.

To test this hypothesis, we performed principal component analysis (PCA) on the refined pseudohelix and wedge models. This technique is commonly used in molecular-dynamics trajectories to produce a reduced-dimensionality representation of conformational space (Roccatano, 2025 ▸). Using an analogous workflow, we performed PCA with respect to the scaled and centered (*Z*-scored) *B* factors, occupancies, and coordinates of all non-H atoms. We then performed *k*-means clustering to identify groups of similar structures, using multiple clustering indices to identify the relevant number of clusters for each set of models (Charrad *et al.*, 2014 ▸).

**Figure 5:**
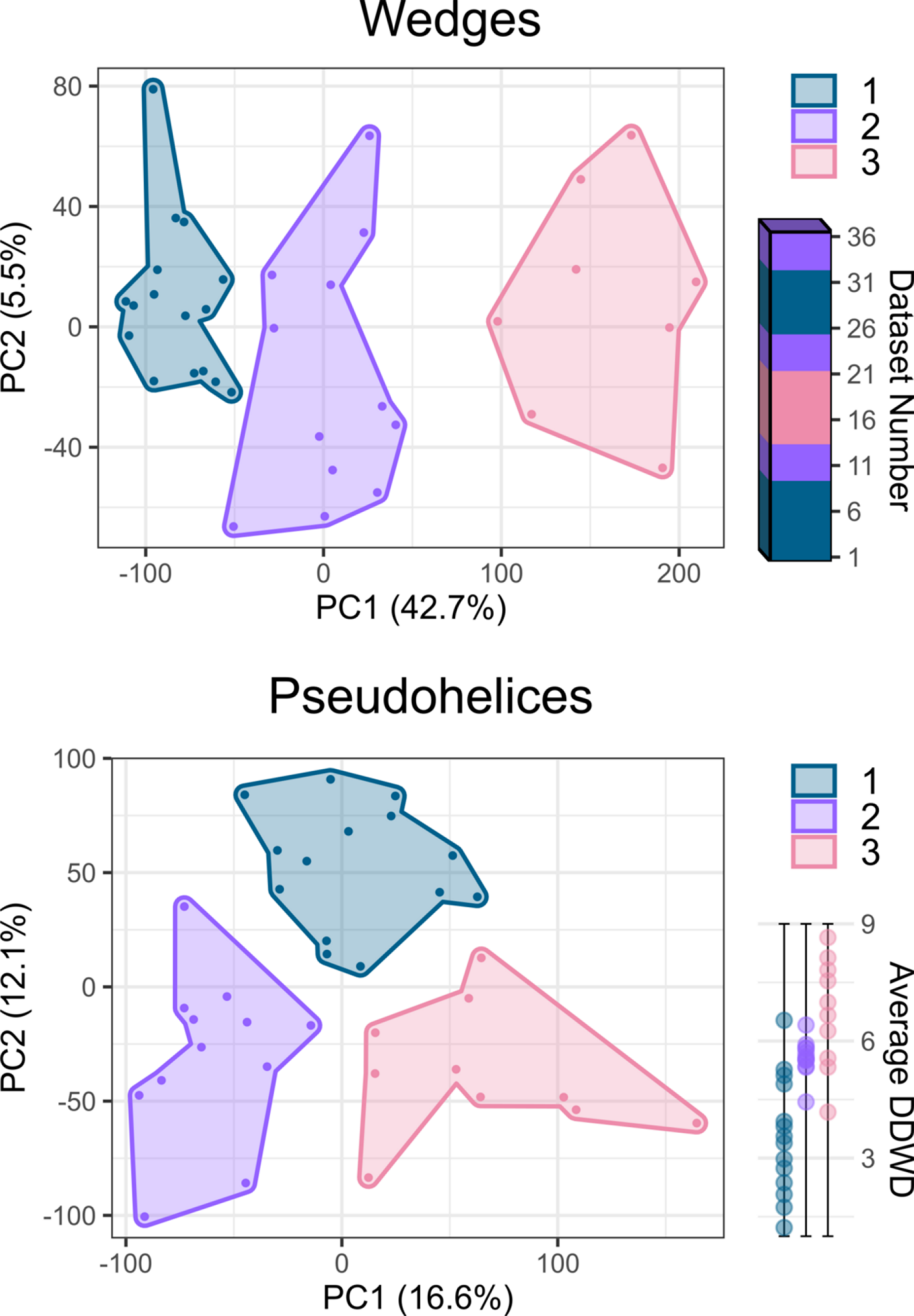
Principal component analysis of wedge (top) and pseudohelix (bottom) structures. Principal component plots (left) were used to conduct *k*-means clustering. Each set of structures was divided into three clusters, represented as navy, purple and pink envelopes (left). The wedge structures are grouped into clusters generally representing the center and extremities of the crystal, and the pseudohelix structures exhibit dose-dependent progression (right).

*K*-means clustering identified three distinct clusters for the pseudohelix and wedge models. While the pseudohelix models appear to transition progressively from one group to another as DDWD increases, the wedge models are grouped into clusters roughly corresponding to central versus terminal wedge positions along the crystal (Fig. 5 ▸, Supplementary Widget S1). This pattern is consistent with wedge variation being driven primarily by heterogeneity between crystal regions, whereas pseudohelix variation is consistent with dose-dependent effects.

### 3.6. Dose-dependent changes at the active site of *Nc*AA9D

To track dose-dependent structural changes in *Nc*AA9D, we developed a custom analysis script in the *R* statistical software (R Core Team, 2025 ▸). We designed the script to record specific measurements of interest for each of the 36 refined pseudohelix models: occupancies of atoms near the active site, copper coordination distances, and active-site angles. The script then calculated a multiple linear regression model for each measurement to determine whether the measurements exhibited dose-dependence, as has been performed successfully in previous reports of radiation damage (Bury *et al.*, 2015 ▸; Ebrahim *et al.*, 2019 ▸). The regression model also allowed us to determine whether the two chains exhibit significantly different dose sensitivity for a given characteristic *via* an interaction term. The detailed regression results and raw data are tabulated in Supplementary Tables S13–S15 and Supplementary Tables S16–S18, respectively. To determine the effects of dose and heterogeneity on the observed electron density, we also calculated isomorphous difference maps between the first and last pseudohelices (Δ*F*_dose_) and wedges (Δ*F*_wedge_), respectively.

Anisotropic unit-cell expansion can mimic apparent movement of atoms, thus necessitating the use of fractional coordinate analysis (Taberman *et al.*, 2019 ▸). *Nc*AA9D exhibits minimal change in unit-cell parameters and unit-cell volume, which indicates negligible lattice expansion over the dose range (Supplementary Fig. S4 and Supplementary Tables S1–S6). Therefore, our interpretation of dose-dependent active-site changes does not require fractional coordinates.

Our determined structures of *Nc*AA9D exhibit characteristics of radiation damage common to all proteins. A general hallmark of radiation damage in protein structures is the cleavage of disulfide bonds, commonly indicated by a negative peak in Δ*F*_dose_ maps (Burmeister, 2000 ▸). Our structures of *Nc*AA9D have two disulfide bonds per chain, all of which exhibit evidence of dose-dependent cleavage in the Δ*F*_dose_ map (Supplementary Fig. S7). No symptoms of disulfide-bond cleavage are present in the Δ*F*_wedge_ maps, further supporting the appropriateness of our data-collection strategy for isolating dose-dependent effects from crystal heterogeneity-dependent effects (Supplementary Fig. S8).

Previous studies have shown that X-ray dose accumulation in LPMOs reduces the active-site copper, leading to the ejection of H_2_O_ax_ and H_2_O_eq_ away from the copper coordination sphere (Gudmundsson *et al.*, 2014 ▸; Muderspach *et al.*, 2019 ▸; Tandrup *et al.*, 2022 ▸). We also observe these effects in our study of *Nc*AA9D: H_2_O_ax_ in both chains and H_2_O_eq_ in chain *A* have been modeled as partial-occupancy alternate conformations at the ‘in’ (H_2_O_in_) and ‘out’ (H_2_O_out_) positions.

We compared the copper–water distances in our structures with those reported in crystallographic (O’Dell, Agarwal & Meilleur, 2017 ▸; Schröder *et al.*, 2022 ▸; Tandrup *et al.*, 2022 ▸), X-ray absorption spectroscopy (Kjaergaard *et al.*, 2014 ▸; Hall *et al.*, 2023 ▸; Joseph *et al.*, 2025 ▸), and computational (Hedegård & Ryde, 2017 ▸) studies. We found that our Cu–H_2_O_in_ and Cu–H_2_O_out_ distances agree with literature-reported values for Cu(II)-LPMO and Cu(I)-LPMO, respectively. These copper–water distances also agree with the definitions put forward by Tandrup *et al.* (2022 ▸): a Cu–H_2_O_eq_ distance of <2.2, 2.2–2.9, or >2.9 Å and a Cu–H_2_O_ax_ distance of <2.7, 2.7–3.2, or >3.2 Å corresponds to a Cu(II)-, mixed-, or Cu(I)-state active site, respectively (Supplementary Fig. S10*a*). For the interested reader, we have compiled a table comparing H_2_O_ax/eq_ structural parameters for all published dose-aware LPMO and all *Nc*AA9D crystal structures (Supplementary Table S19). Taken together, the ‘in’ and ‘out’ water positions correspond to the Cu(II) and Cu(I) states, respectively.

**Figure 6:**
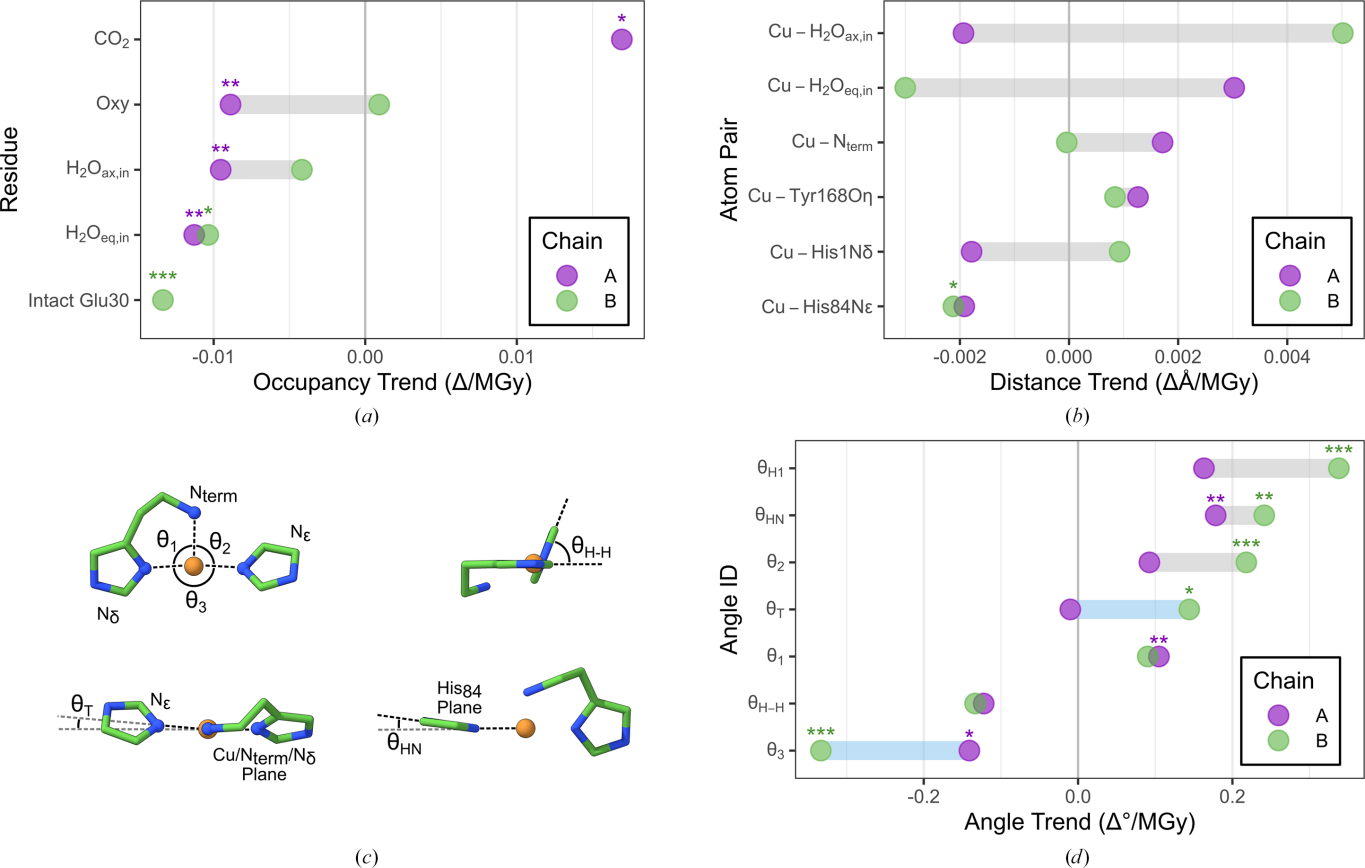
Dose-dependent trends in (*a*) occupancies, (*b*) distances and (*d*) angles for chain *A* (purple) and chain *B* (green). Statistical significance of individual trends is marked with asterisks (*, *p* < 0.05; **, *p* < 0.01; ***, *p* < 0.0001) and statistically significant contrasts between chains are highlighted with a light blue bar. (*c*) Schematic of the angles measured. θ_1_, θ_2_ and θ_3_ represent the angle formed between the copper and two of its nitrogen ligands. θ_H–H_ is the acute angle between the imidazole ring planes of His1 and His84. θ_T_ is the angle between the Cu–N_term_–His1 N^δ^ plane and the Cu–His84 N^ɛ^ line. θ_H1_ and θ_HN_ denote angles between an imidazole ring plane and a line extending from the copper to the copper-coordinating atom in the same histidine. For *Nc*AA9D, θ_H1_ corresponds to His1 and θ_HN_ to His84. The measurement of θ_HN_ is illustrated above. For this study, the imidazole ring planes were defined by the coordinates of their N^δ^, N^ɛ^ and C^γ^ atoms.

**Figure 7:**
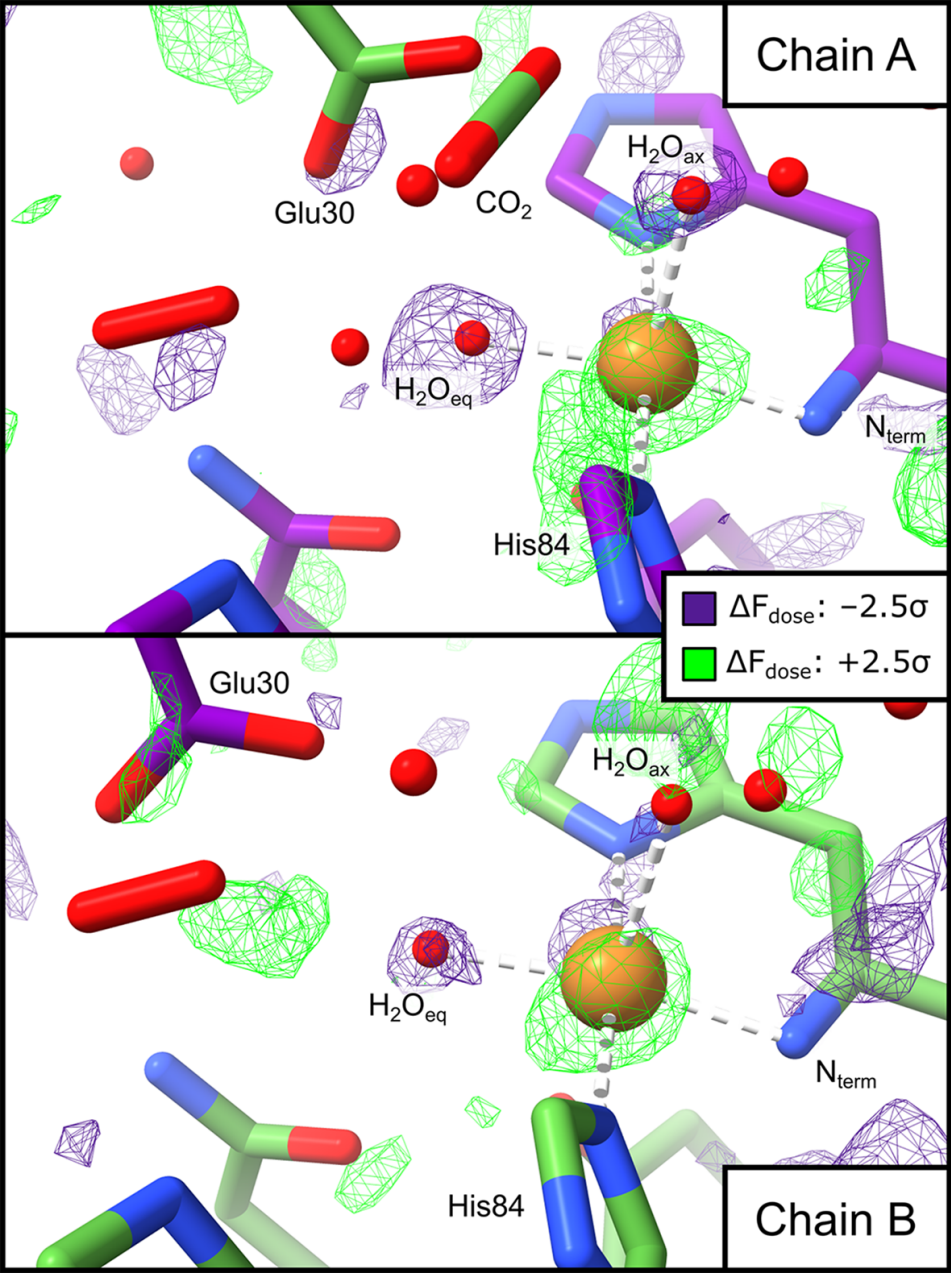
Dose-dependent electron-density changes at the active and pre-bound sites of chain *A* (top, purple) and chain *B* (bottom, green). The Δ*F*_dose_ map is contoured at ±2.5σ and represented as a green (positive) and purple (negative) mesh. Negative features at H_2_O_ax_ and H_2_O_eq_ in both chains indicate photoreduction of the active-site copper in both chains. Negative Δ*F*_dose_ features at the dioxygen species in chain *A* and Glu30 in chain *B* indicate that specific radiation damage affects this pre-bound dioxygen site more than the other. Δ*F*_dose_ density at the copper site shows adjacent positive and negative features, consistent with a slight dose-dependent shift of the copper position.

**Figure 8:**
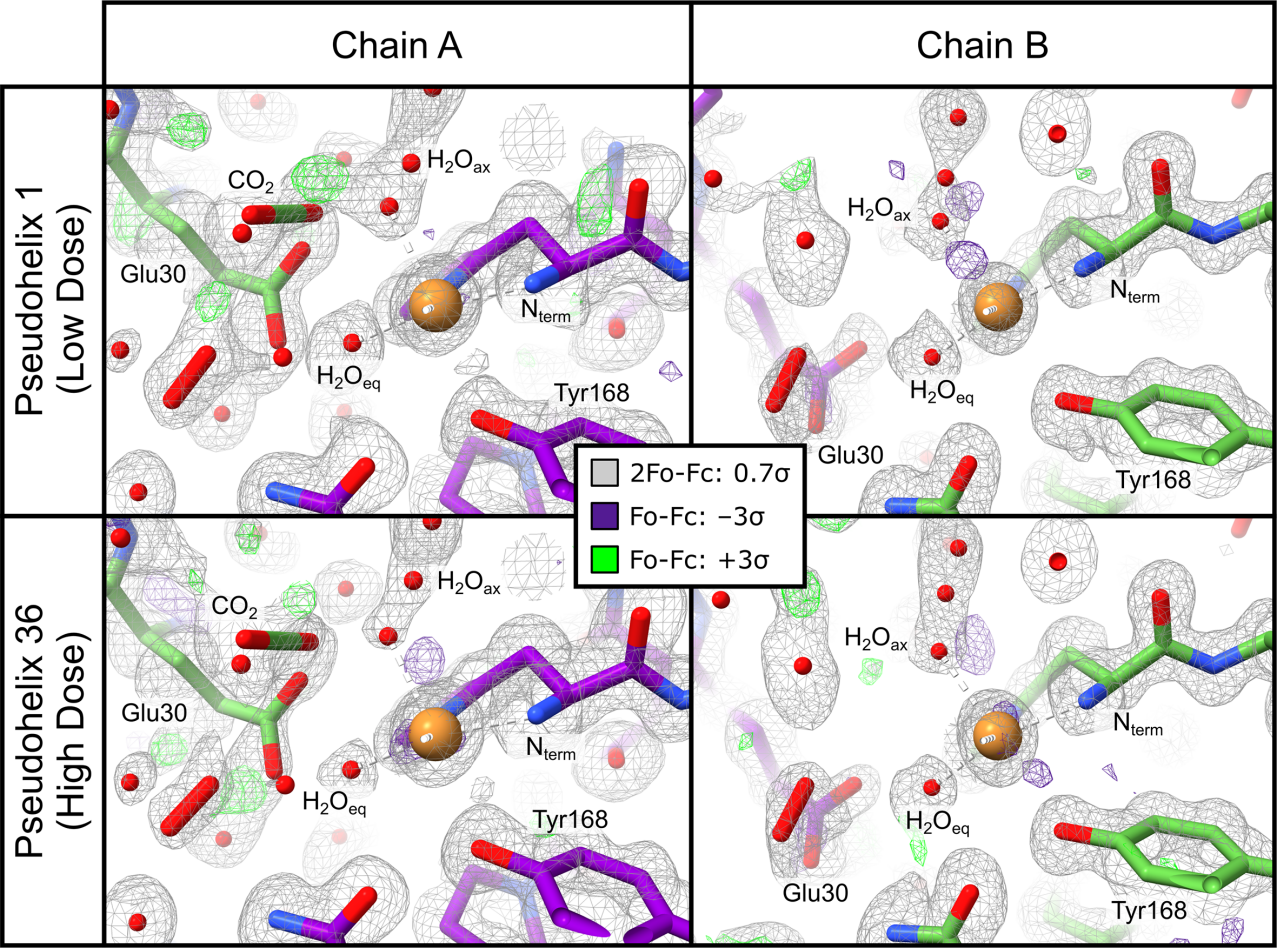
Electron-density maps of the active and pre-bound dioxygen sites of chain *A* (purple) and chain *B* (green) at low and high X-ray doses. The electron density visibly spreads out at H_2_O_ax_ upon reduction of the active-site copper. For all models, the occupancies of H_2_O_ax_, H_2_O_eq_, CO_2_ and the pre-bound dioxygen species are allowed to change. The 2*mF*_o_ − *DF*_c_ electron density is contoured at 1.0σ and represented as a gray mesh. The *mF*_o_ − *DF*_c_ electron density is contoured at ±3σ and represented as a green (positive) and purple (negative) mesh. The active-site copper is shown as an orange sphere, waters as red spheres and dioxygen as a red stick.

We see a negative dose-dependent trend in the occupancies of H_2_O_ax,in_ and H_2_O_eq,in_ in chain *A* and only H_2_O_eq,in_ in chain *B*, indicating more pronounced photoreduction of the copper in chain *A* (Fig. 6 ▸*a*). Further supporting our occupancy findings, the Δ*F*_dose_ map shows clear negative density at H_2_O_ax,in_ and H_2_O_eq,in_ in chain *A* and at H_2_O_eq,in_ in chain *B* (Fig. 7 ▸). This feature is accompanied by a ‘smearing out’ of the electron density, which can be observed when comparing the 2*mF*_o_ − *DF*_c_ maps from the lowest and highest dose datasets (Fig. 8 ▸). In their dose-series study, Tandrup *et al.* (2022 ▸) report analogous ‘in/out’ water behavior in *Ls*AA9A (with occupancies locked at 0.5), and their electron-density maps exhibit qualitatively similar evolution with dose.

In an extensive study on radiation damage to *Ls*AA9A and *Ta*AA9A, Tandrup *et al.* (2022 ▸) measured multiple active-site angles, θ_1_, θ_2_, θ_3_, θ_T_, θ_H–H_, θ_H1_, and θ_HN_, which we evaluated across all our *Nc*AA9D structures (Fig. 6 ▸*c*). In our dataset, we observe significant dose-dependent trends for multiple angle parameters (Fig. 6 ▸*d*). Tandrup *et al.* (2022 ▸) suggested that a θ_3_ angle below 170° and a θ_T_ angle above 3° could signify a photoreduced active site. In our analysis, θ_T_ exhibits a small magnitude trend, and only in chain *B* (*p* = 0.011). For θ_3_, negative dose-dependent trends were observed in both chains; θ_3_ ranges from 172.9° to 168.6° in chain *A* and from 173.9° to 169.8° in chain *B*. Both chains in *Nc*AA9D reach the θ_3_ photoreduction criterion proposed by Tandrup *et al.* (2022 ▸). However, despite exposing our crystals to high X-ray doses generally higher than those used by Tandrup *et al.* (2022 ▸) (Supplementary Fig. S10*b*), the overall magnitude of change observed across our structures (∼4°) is smaller than that (≥5°) observed by Tandrup *et al.* (2022 ▸). While our results support the dose-sensitivity of θ_3_ in LPMOs, they also suggest a dependence on the ortholog and crystal morphology being studied.

In our analysis, we measured copper coordination distances across all of our *Nc*AA9D structures and detected a small-magnitude dose-dependent decrease in the Cu–His84 N^ɛ^ distance only in chain *B* (*p* = 0.028; Fig. 6 ▸*b*). The Cu–His84 N^ɛ^ distance shortened by a total of 0.026 Å in chain *B* during the dose series, which is close to the bond-distance error of ±0.017 Å calculated using the DPI (Kumar *et al.*, 2015 ▸).

Taken together, we observe progressive dose-dependent changes in *Nc*AA9D consistent with photoreduction, as tracked by active-site occupancies, distances, and angles. Although several active-site parameters change systematically with dose, the overall response remains modest, even at high doses. This contrasts with the observations of Tandrup *et al.* (2022 ▸), who reported that the reduced form of LPMOs could be obtained at ∼1 MGy. We attribute our relatively small-magnitude structural changes to a combination of three possible effects. Firstly, we emphasize that the dose values in the present work represent a weighted average over the crystal volumes and therefore do not imply that the entire diffracting volume has experienced the dose reported by average DDWD values. This effect has been explored in detail in Section 3.2 and means even the highest average dose datasets can retain a measurable low-dose contribution, which plausibly contributes to the persistence of ‘in’ waters at high dose and the absence of a clearly fully photoreduced endpoint. Secondly, we acknowledge that *in crystallo*, the active site of *Nc*AA9D is sequestered from the bulk solvent. Radiation-damage effects are largely mediated by radiation-induced moieties generated in the bulk solvent (Garman, 2010 ▸). Due to the unique arrangement of the *Nc*AA9D chains, these reducing agents may have less access to the buried *Nc*AA9D active site, thus reducing its rate of photoreduction compared with other LPMOs. Thirdly, we acknowledge that cryogenic temperatures significantly reduce protein flexibility, which can result in the trapping of radiation-damage intermediates in a buried active site (Colletier *et al.*, 2008 ▸; Koulas *et al.*, 2026 ▸). This effect may manifest itself in our structures, where the expulsion of copper-coordinated waters requires the rearrangement of a well ordered solvent network and its surrounding protein residues. In this case, the rate of copper photoreduction could outpace the rate of water movement, making our observed reduction rates lower than the true rate.

Together, these considerations suggest that the apparent discrepancy with previous studies on LPMO photoreduction may reflect differences in crystal form/packing and active-site solvent accessibility, as well as the spatial distribution of dose within the illuminated volume, rather than a true difference in radiation sensitivity.

### 3.7. Dose-dependent changes in the pre-bound dioxygen site of *Nc*AA9D

We also tracked dose-dependent changes in the pre-bound dioxygen site of *Nc*AA9D, located adjacent to the equatorial copper coordination site. A commonly recognized sign of specific radiation damage is the decarboxylation of glutamate and aspartate residues (Burmeister, 2000 ▸). We observe dose-dependent decarboxylation of Glu30 in chain *B*. Our statistical analysis revealed a negative dose-dependent trend in the occupancy of intact Glu30 in chain *B* (*p* = 2.83 × 10^−4^). This is further supported by negative Δ*F*_dose_ density at the carboxyl group of this residue (Fig. 7 ▸*a*) and the successful modeling of a CO_2_ molecule near Glu30 in chain *B* (Fig. 8 ▸). CO_2_ has been observed near sites of radiation damage-related decarboxylation in other protein crystal structures (Arnott *et al.*, 2017 ▸).

Interestingly, we observe a concurrent dose-dependent decrease in the occupancy of the pre-bound dioxygen species in chain *A* (*p* = 0.007) but not chain *B* (*p* = 0.922) (Fig. 6 ▸*a*). The occupancy trend of the pre-bound dioxygen species in chain *A* is further supported by a corresponding negative peak in the Δ*F*_dose_ map (Fig. 7 ▸*a*) and a shrinking of the observed electron density observable upon comparison of the lowest and highest dose 2*mF*_o_ − *DF*_c_ maps (Fig. 8 ▸).

Although dose-response slopes do not differ significantly between chains, the active and pre-bound dioxygen site of chain *A*, and the nearby Glu30 in chain *B*, appear more radiation-sensitive, consistent with our previous reports of asymmetric oxygen activation in *Nc*AA9D. Using X-ray and neutron crystallography, we have previously observed the formation of activated dioxygen species in only chain *A* of *Nc*AA9D upon the reduction of crystals with ascorbate (O’Dell, Agarwal & Meilleur, 2017 ▸; O’Dell, Swartz *et al.*, 2017 ▸; Schröder *et al.*, 2021 ▸, 2022 ▸). Interestingly, the chain exhibiting O_2_ activation upon chemical reduction in previous studies is the same chain more impacted by radiation damage in our dose series. Combined, our previous and current studies imply that when crystallized under these conditions, one active site of *Nc*AA9D is more readily reduced than the other. This behavior appears to occur independently of the electron source (whether from conventional chemical reductants or water radiolysis).

## 4. Conclusion

Metalloproteins are especially sensitive to radiation, and LPMOs are no exception to this tendency. Multiple studies of LPMOs have revealed that photoreduction of their active-site copper results in expulsion of the axial and equatorial waters from the copper coordination sphere (Gudmundsson *et al.*, 2014 ▸; Muderspach *et al.*, 2019 ▸; Tandrup *et al.*, 2022 ▸). We observe a similar effect in our study of *Nc*AA9D, supported by statistical analysis of atomic occupancies and Δ*F*_dose_ maps. Additionally, we find that the movement of the waters away from the active-site copper creates a characteristic ‘smearing out’ of electron density (Fig. 8 ▸). The electron density of the disordered waters looks quite like the electron density of a partial-occupancy dioxygen species and is therefore easy, even tempting, to misinterpret.

In their study on *Ls*AA9A and *Ta*AA9A, Tandrup *et al.* (2022 ▸) went beyond previous studies and explored the possibility of other effects of photoreduction. Principally, they observed changes in active-site coordination angles with increasing X-ray dose and suggested that the angles θ_3_ and θ_T_ can be used as a diagnostic for LPMO photoreduction. While we observed dose-dependent trends in some active-site angles of *Nc*AA9D, our observations are different from those previously reported. This indicates that redox state- and dose-responsive active-site geometry is ortholog-specific, highlighting the importance of analyzing active-site geometry with careful consideration of orthological and crystal form contexts.

The axial and equatorial waters may be the most reliable proxy measure of copper redox state in LPMOs, but the results presented here caution against interpreting such features with heavy-handedness and indicate that other techniques may be more effective. Direct measurement of the LPMO copper is likely to provide the most reliable indication of its redox state. X-ray absorption (XAS; Hemsworth *et al.*, 2013 ▸; Kjaergaard *et al.*, 2014 ▸; Paradisi *et al.*, 2019 ▸; Zhao *et al.*, 2023 ▸), electron paramagnetic resonance (Kjaergaard *et al.*, 2014 ▸; Liao *et al.*, 2025 ▸), UV–visible (Kjaergaard *et al.*, 2014 ▸; Kracher *et al.*, 2018 ▸) and fluorescence (Bissaro *et al.*, 2016 ▸; Ayuso-Fernández *et al.*, 2024 ▸) spectroscopies have all been used successfully to track the redox state of the LPMO copper site. The low copper concentration coupled with weak signal from LPMO crystals makes UV–visible microspectrophoto­metry difficult to usefully interpret (Gudmundsson *et al.*, 2014 ▸). XAS has been used successfully for *in crystallo* studies of LPMO reduction (Hemsworth *et al.*, 2013 ▸), and fluorescence spectroscopy may provide a nondestructive handle to probe the copper redox state.

We further find that the apparent dose response can depend on the physical distribution of dose within the crystal, including heterogeneous or anisotropic dose deposition arising from factors such as beam profile, mother-liquor composition, and crystal morphology/orientation (Atakisi *et al.*, 2019 ▸). Although DDWD provides a valuable way to account for burned-out crystal regions, it is still a single-point summary of a distribution which may exhibit atypical characteristics such as skewness, kurtosis, or multimodality. When relying on dose calculations for data analysis, especially of data which will likely result in a complex dose distribution, scientists should consider analysis aware of the whole weighted dose distribution. Different single-point measures may also prove effective: for example, the median DDWD could provide a more reliable estimate of the ‘true’ central tendency and is not drawn out as much by outlier crystal regions.

This study also underscores the importance of statistically guided, multi-dataset structural biology. Although regularity is an inherent property of protein crystals, they, and the datasets collected from them, are heterogeneous. Different datasets may exhibit non-isomorphism or outlier behavior, which makes simple pairwise comparisons between endpoints risky. Multi-dataset analysis explicitly addresses this challenge, leveraging information across datasets to isolate systematic experimental effects from noise. For example, *PanDDA* uses comparison across multiple datasets to identify statistically significant ‘event’ sites against random electron-density variability (Pearce *et al.*, 2017 ▸; Weiss *et al.*, 2022 ▸), and other studies have utilized statistical methods to isolate dose-dependent effects from background noise in radiation dose-series experiments (Borek *et al.*, 2013 ▸; Ebrahim *et al.*, 2019 ▸). Here, we demonstrate the application of multiple linear regression and generalized least squares in dose-series crystallography, illustrating the breadth of well established statistical frameworks available for multi-dataset analysis.

Based on these insights and a rapidly growing body of research on radiation damage, we, as others before us, advise caution during the modeling of LPMO structures, especially when interpreting active-site features. Minimizing dose accumulation during X-ray crystallography experiments is the obvious recommendation to preserve biologically relevant features of a protein structure, especially in radiation-sensitive proteins such as LPMOs. However, the structures presented here show that modeling metal-bound dioxygen species is challenging even when doses are kept well below recommended thresholds. An alternative approach to collecting a single low-dose dataset is to collect a radiation dose series from a single crystal to track and support the unambiguous assignment of radiation-sensitive intermediates. While the crystal used for the study presented here is of unusually large dimensions, advances in synchrotron-based instrumentation allow similar studies to be conducted from much smaller crystals. Intentional data-collection schemes, including traditional crystallography methods and newly emerging serial techniques such as serial femtosecond crystallography, wedged serial crystallography, or synchrotron serial crystallography offer promising approaches to mitigate radiation damage (Bury *et al.*, 2018 ▸; Nass, 2019 ▸; Chaussavoine *et al.*, 2022 ▸; Nam, 2024 ▸).

## Supplementary Material

Supplementary Figures and Supplementary Tables S1-S18. DOI: 10.1107/S205979832600639X/xh5065sup1.pdf

Supplementary Table S19. DOI: 10.1107/S205979832600639X/xh5065sup2.xlsx

Supplementary Widget S1. DOI: 10.1107/S205979832600639X/xh5065sup3.html

Supplementary Movie S1. DOI: 10.1107/S205979832600639X/xh5065sup4.mp4

Diffraction images for wedges 1-19.: https://doi.org/10.18430/M3.IRRMC.7182

Diffraction images for wedges 20-38.: https://doi.org/10.18430/M3.IRRMC.7183

PDB reference: *Nc*AA9D, pseudohelices, 9z8o

PDB reference: 9z8p

PDB reference: 9z8q

PDB reference: 9z8r

PDB reference: 9z8s

PDB reference: 9z8t

PDB reference: 9z8u

PDB reference: 9z8v

PDB reference: 9z8w

PDB reference: 9z8x

PDB reference: 9z8y

PDB reference: 9z8z

PDB reference: 9z90

PDB reference: 9z92

PDB reference: 9z93

PDB reference: 9z94

PDB reference: 9z95

PDB reference: 9z96

PDB reference: 9z97

PDB reference: 9z98

PDB reference: 9z99

PDB reference: 9za5

PDB reference: 9za6

PDB reference: 9za7

PDB reference: 9za8

PDB reference: 9za9

PDB reference: 9zaa

PDB reference: 9zab

PDB reference: 9zac

PDB reference: 9zad

PDB reference: 9zae

PDB reference: 9zaf

PDB reference: 9zah

PDB reference: 9zai

PDB reference: 9zaj

PDB reference: 9zal

PDB reference: wedges, 9zam

PDB reference: 9zan

PDB reference: 9zaq

PDB reference: 9zar

PDB reference: 9zas

## Data Availability

The diffraction images used in this study have been deposited at The Integrated Resource for Reproducibility in Macromolecular Crystallography: https://doi.org/10.18430/M3.IRRMC.7182 (wedges 1–19) and https://doi.org/10.18430/M3.IRRMC.7183 (wedges 20–38). The atomic coordinates and structure factors for all pseudohelices and wedges 1, 9, 18, 27. and 36 have been deposited in the Protein Data Bank (https://www.rcsb.org/). All other structures and associated data are available from the authors upon request. The *R* scripts and *Bash* convenience scripts, along with their associated input files, have been deposited on GitHub and are accessible at https://github.com/sadamill/NcAA9D_DoseSeries.
