# Supplementary material for: Dose-dependent structural and electron-density features in the lytic polysaccharide monooxygenase NcAA9D
Source: Acta Crystallogr D Struct Biol. 2026 Jul 28;82(Pt 8):900–14. doi: 10.1107/S205979832600639X (PMC13431641; doi:10.1107/S205979832600639X)
Supplement: Supplementary file 1 [file d-82-00900-sup1.pdf]

STRUCTURAL
BIOLOGY

Volume 82 (2026)

Supporting information for article:

Dose-dependent structural and electron-density features in the lytic polysaccharide monooxygenase *Nc*AA9D

Samuel A. Miller, William B. O'Dell and Flora Meilleur

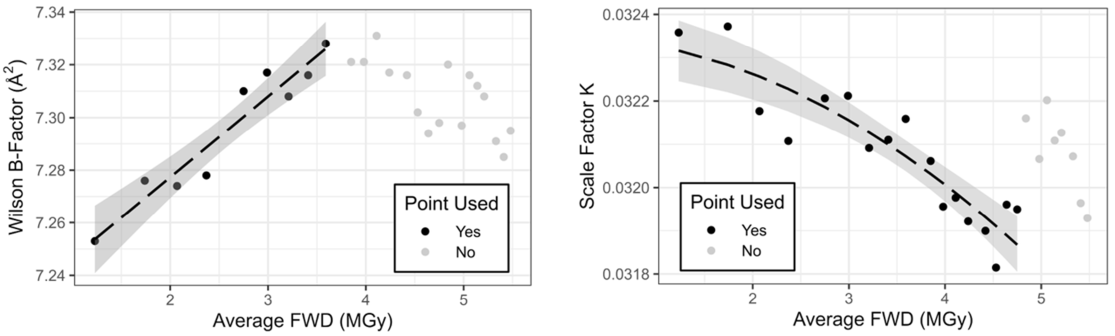

$$B = B_0 + \beta D$$

$$B_0 = 7.2160$$

$$\beta = 0.0307$$

$$K = \frac{1}{\text{Wilson Scale}} = c \times e^{-\gamma^2 D^2}$$

$$c = 0.0323$$

$$\gamma = 0.0257$$

Figure S1 Estimation of intensity decay parameters by least squares regression. The dose dependence of the Wilson B-factor was estimated by linear least squares regression, excluding points which didn’t exhibit a linear trend. The dose dependence of the scaling factor K was estimated by nonlinear least squares, again excluding points which didn’t exhibit the expected trend shape. Points included in regression analysis are colored black, and those excluded are colored light gray. The estimated trendlines are marked by a dashed line, with a 95% confidence interval shaded in gray. Confidence intervals for nonlinear least squares regression were calculated with the R package propagate (v1.1.0) (Spiess, 2026).

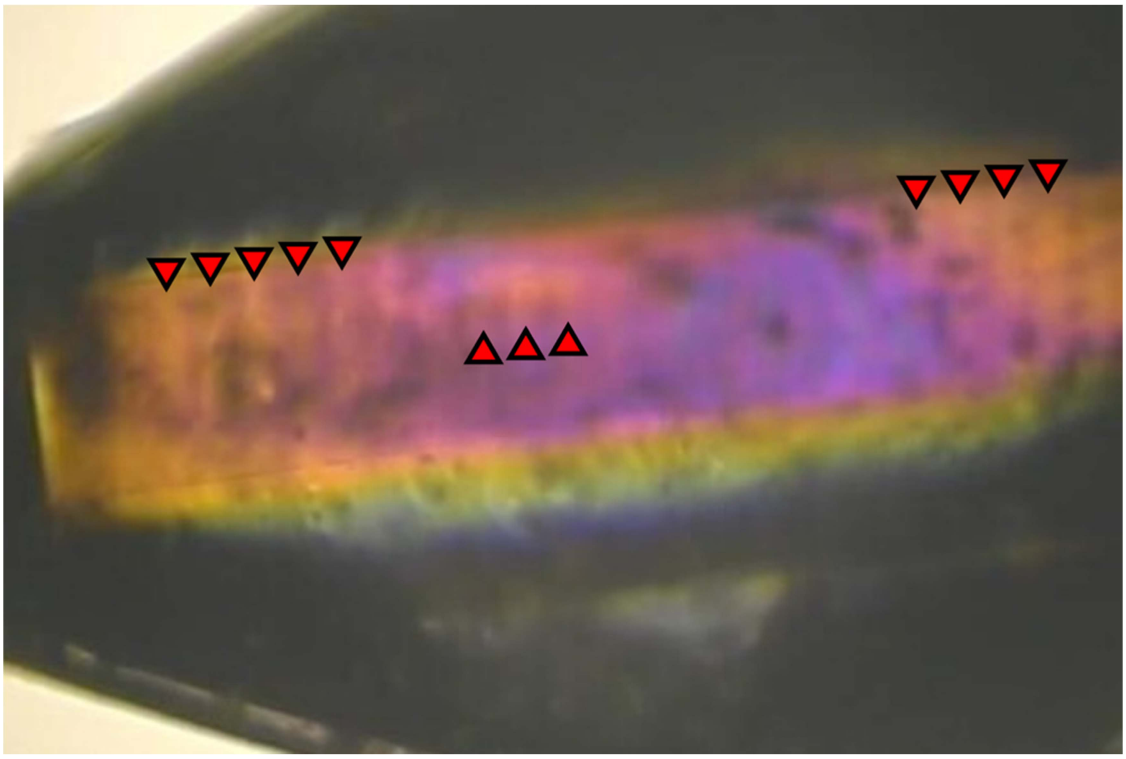

Figure S2 “Zebra stripe” pattern visible on the *Nc*AA9D crystal after multi-wedge data collection.

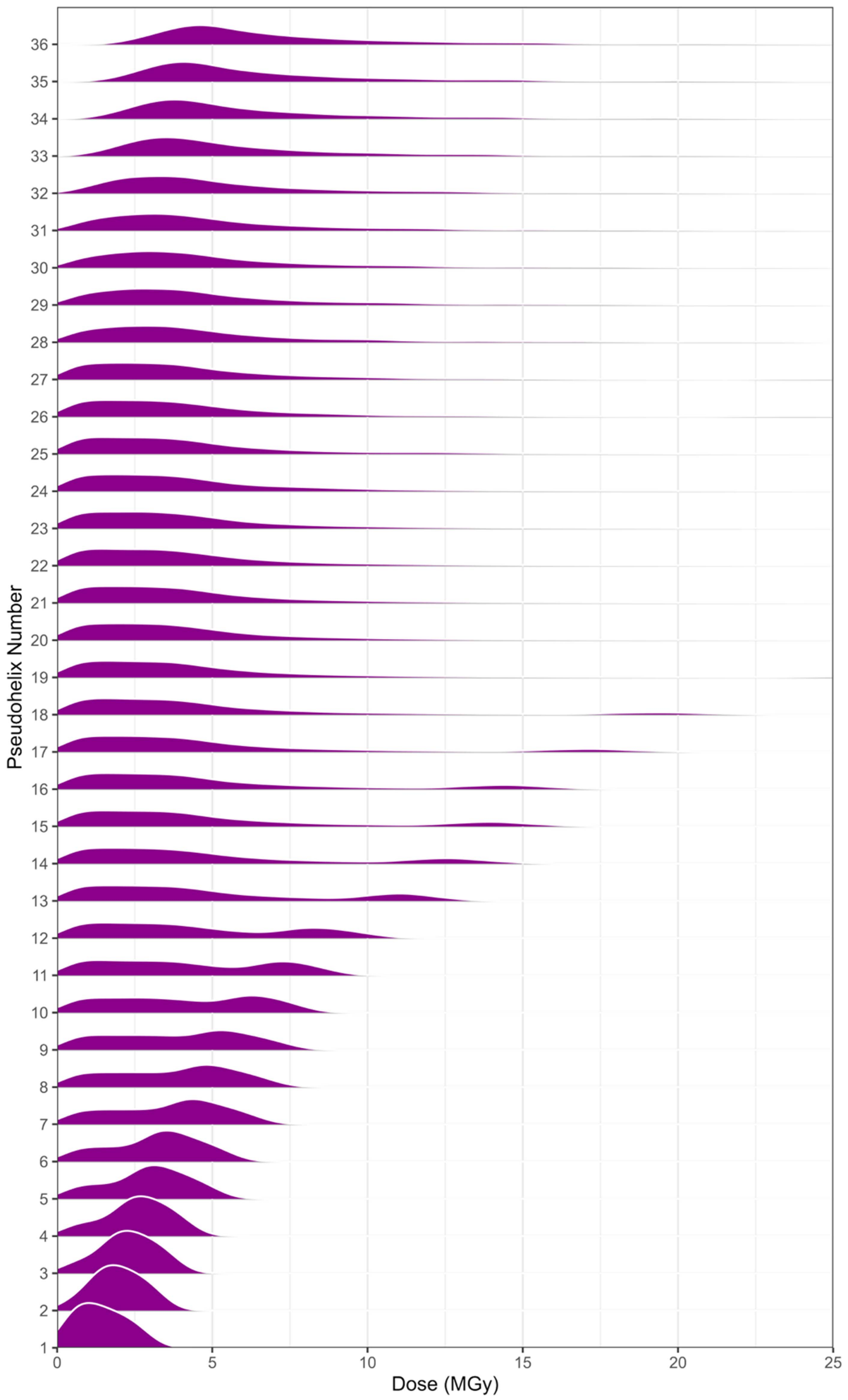

Figure S3 Fluence- and diffraction decay-weighted dose distributions of pseudohelices 1–36. As more dose accumulates at the center of the crystal, voxels with anomalously high dose begin to "run off" from the rest of the dose distribution.

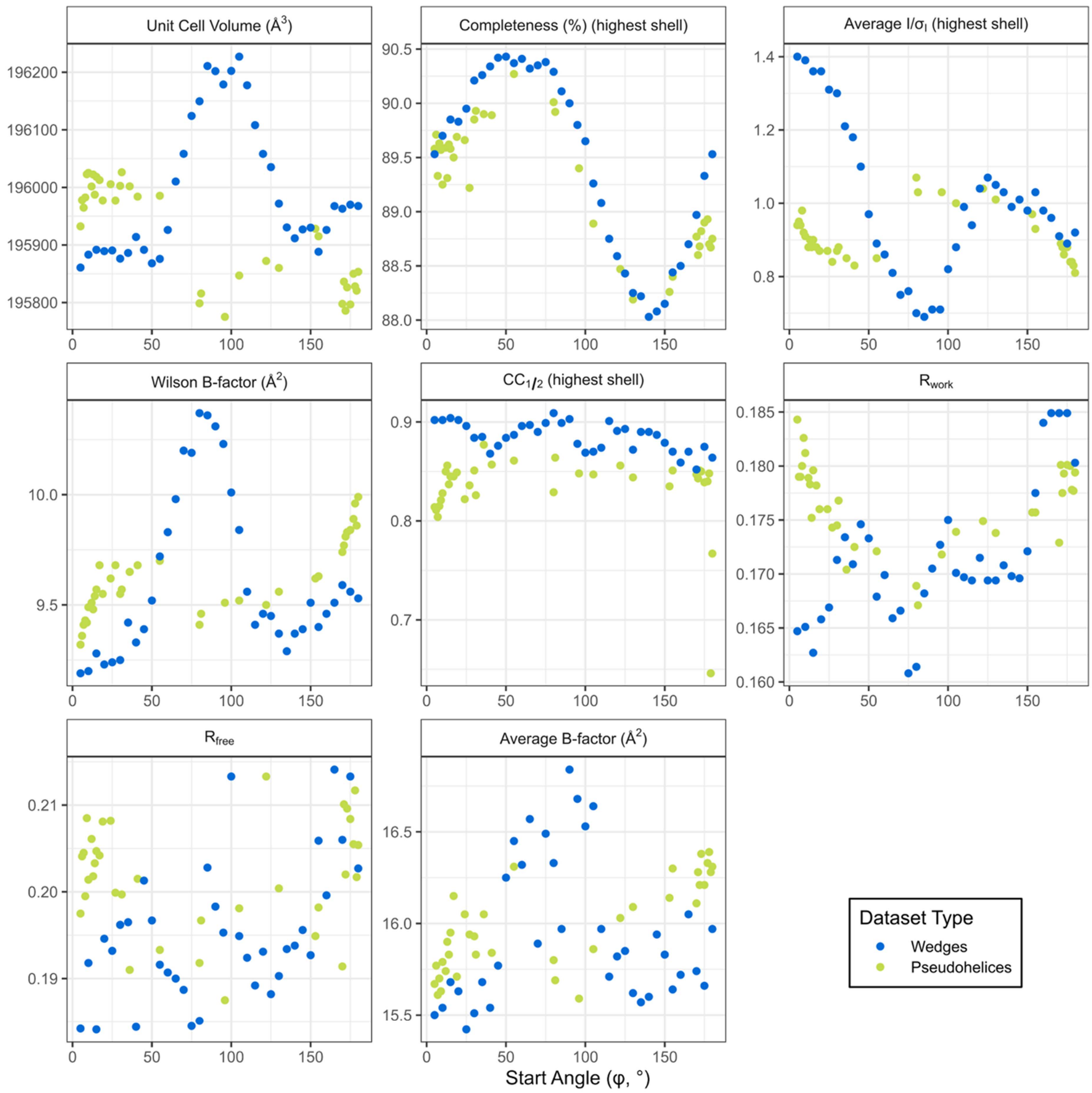

Figure S4 Data collection and processing statistics scatterplots. The wedge datasets exhibit non-monotonic trends in unit cell volume, Wilson B-factor, and average B-factor, which suggest more disorder at the center of the crystal. While the pseudohelices exhibit minimal unit cell volume expansion, they exhibit an overall increase in Wilson B-factor and average B-factor. The highest resolution shell is 1.11 to 1.1 Å.

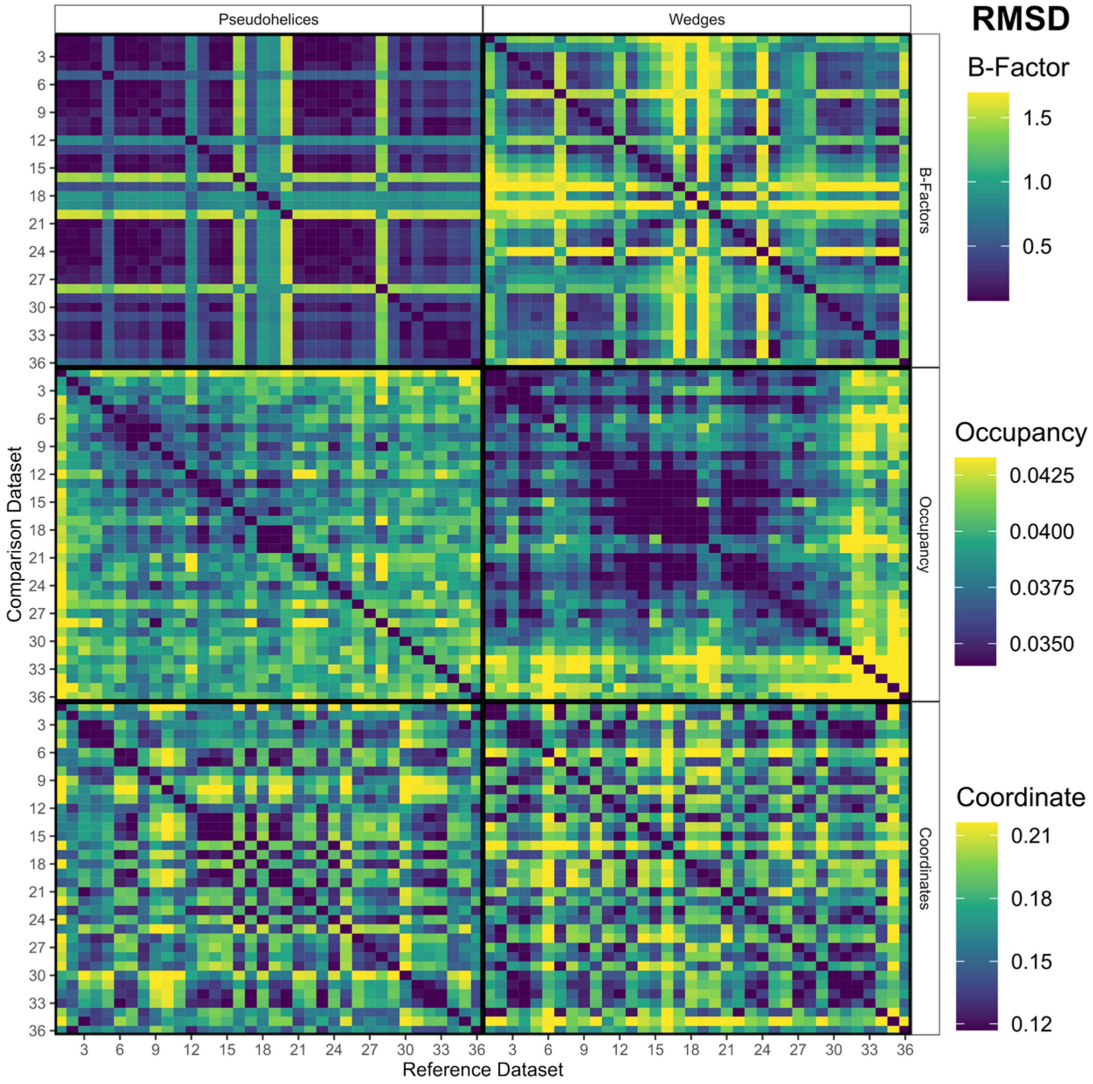

Figure S5 Pairwise RMSD comparisons between wedges and pseudohelices.

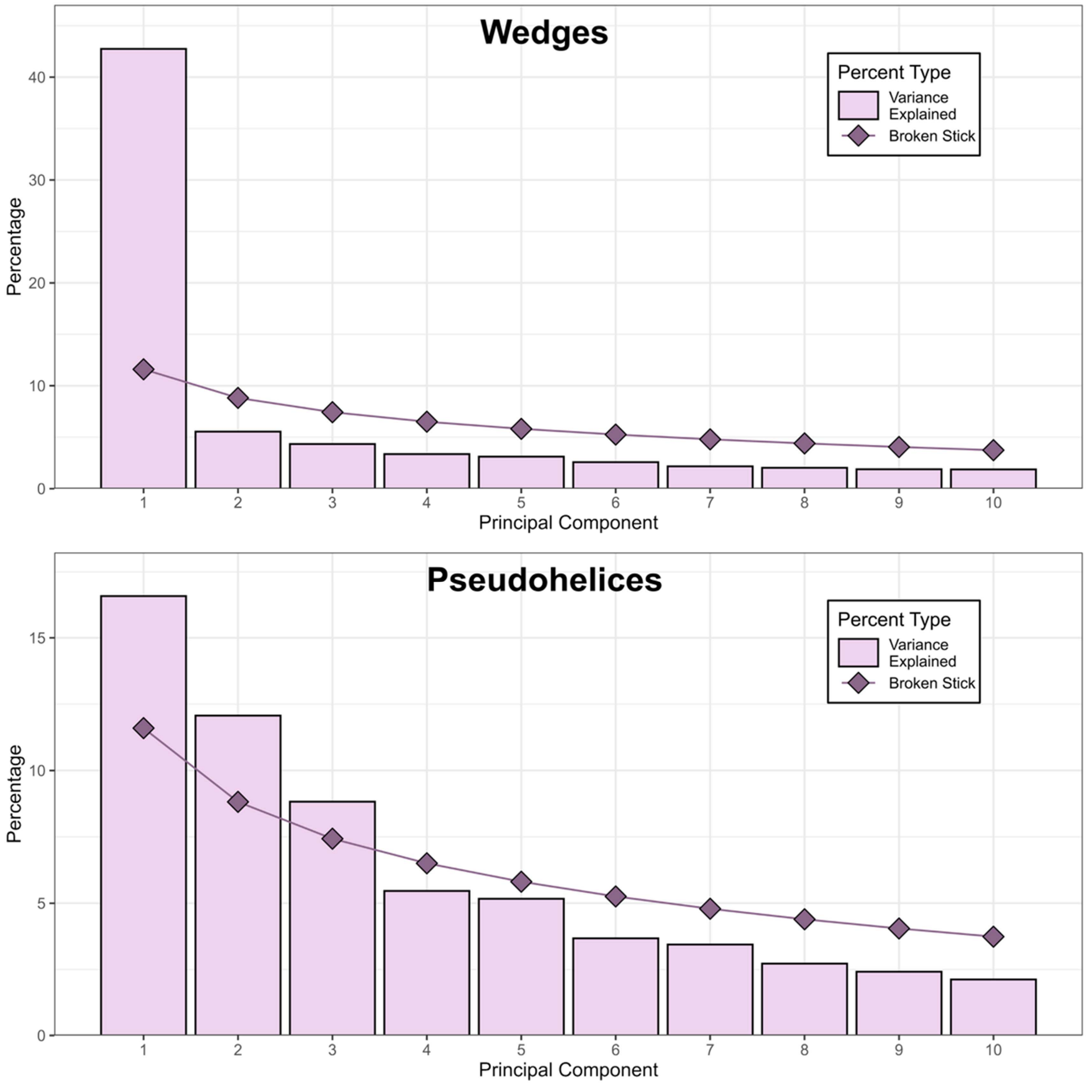

Figure S6 Broken stick model to determine the number of significant components. A component is considered significant if its observed variance (pink bars) is larger than the variance from random data (mauve diamonds). Two principal components were kept for the wedges (for ease of plotting) and three were kept for the pseudohelices.

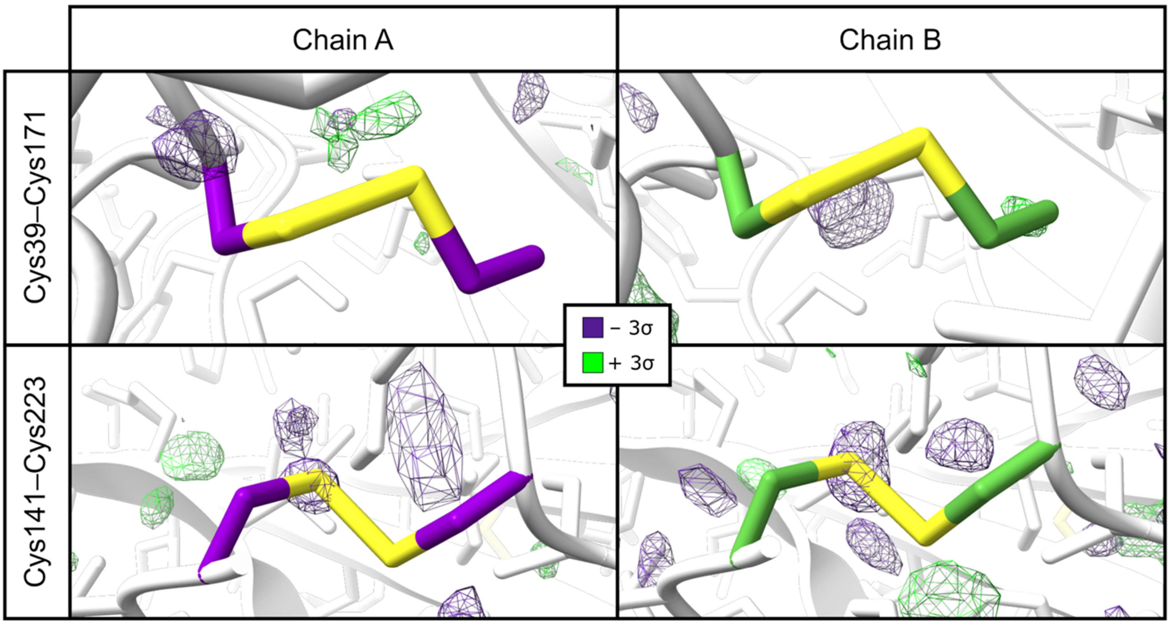

Figure S7 Radiation damage of *Nc*AA9D at disulfide bonds. The ΔF_{dose} maps represent the difference in electron density between the highest- and lowest-dose pseudohelix datasets. They are contoured at ±3.0σ and represented as a green (positive) and purple (negative) mesh. Significant negative ΔF_{dose} peaks at the C-terminal cysteines indicate specific radiation damage in the form of disulfide bond cleavage. Residues belonging to chain A are colored purple, and those belonging to chain B are colored green.

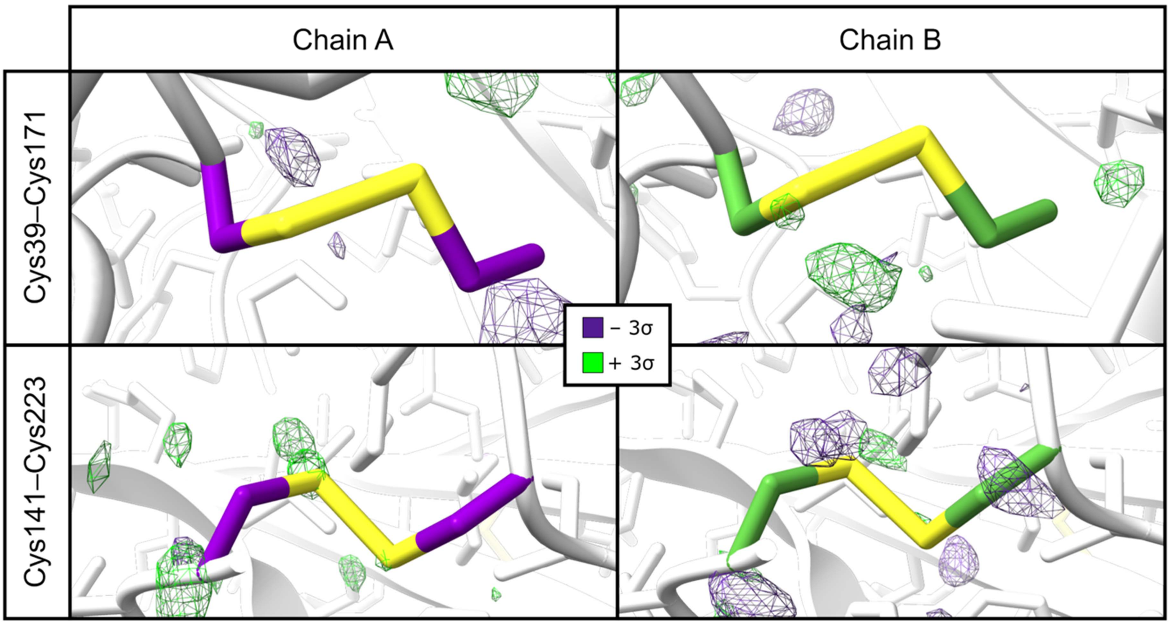

Figure S8 Crystal position-dependent electron density shifts in NcAA9D at disulfide bonds. The ΔF_{wedge} maps represent the difference in electron density between the last and first wedge datasets. They are contoured at ±3.0σ and represented as a green (positive) and purple (negative) mesh. There are no significant negative peaks at any of the disulfide bonds. Residues belonging to chain A are colored purple, and those belonging to chain B are colored green.

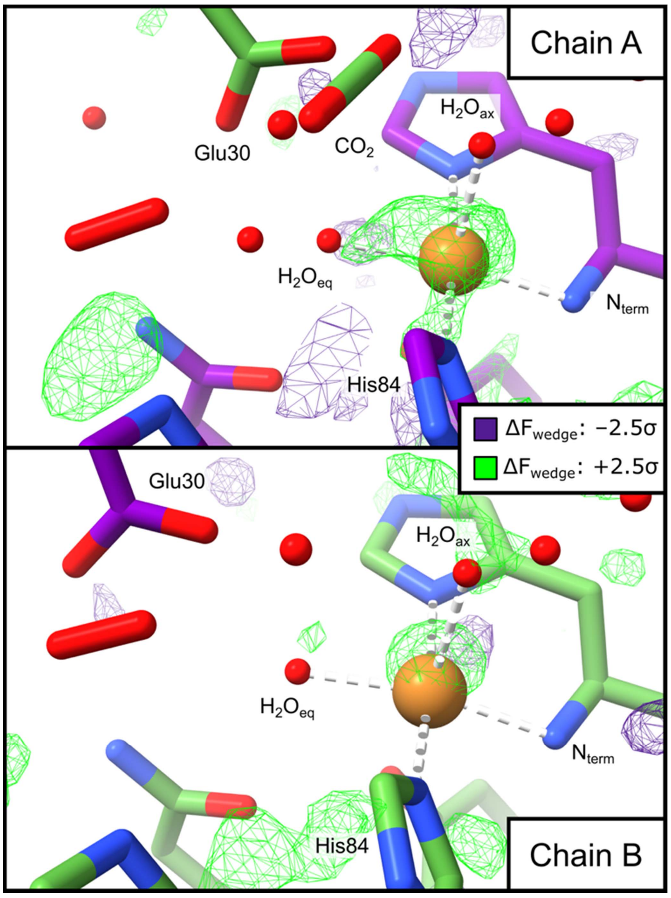

Figure S9 Crystal position-dependent electron density changes at the active and pre-bound sites of chain A (top, purple) and chain B (bottom, green). The ΔF_{wedge} map is contoured at ±2.5σ and represented as a green (positive) and purple (negative) mesh. There is a lack of significant features, indicating insignificant changes dependent on where the dataset was taken in the crystal.

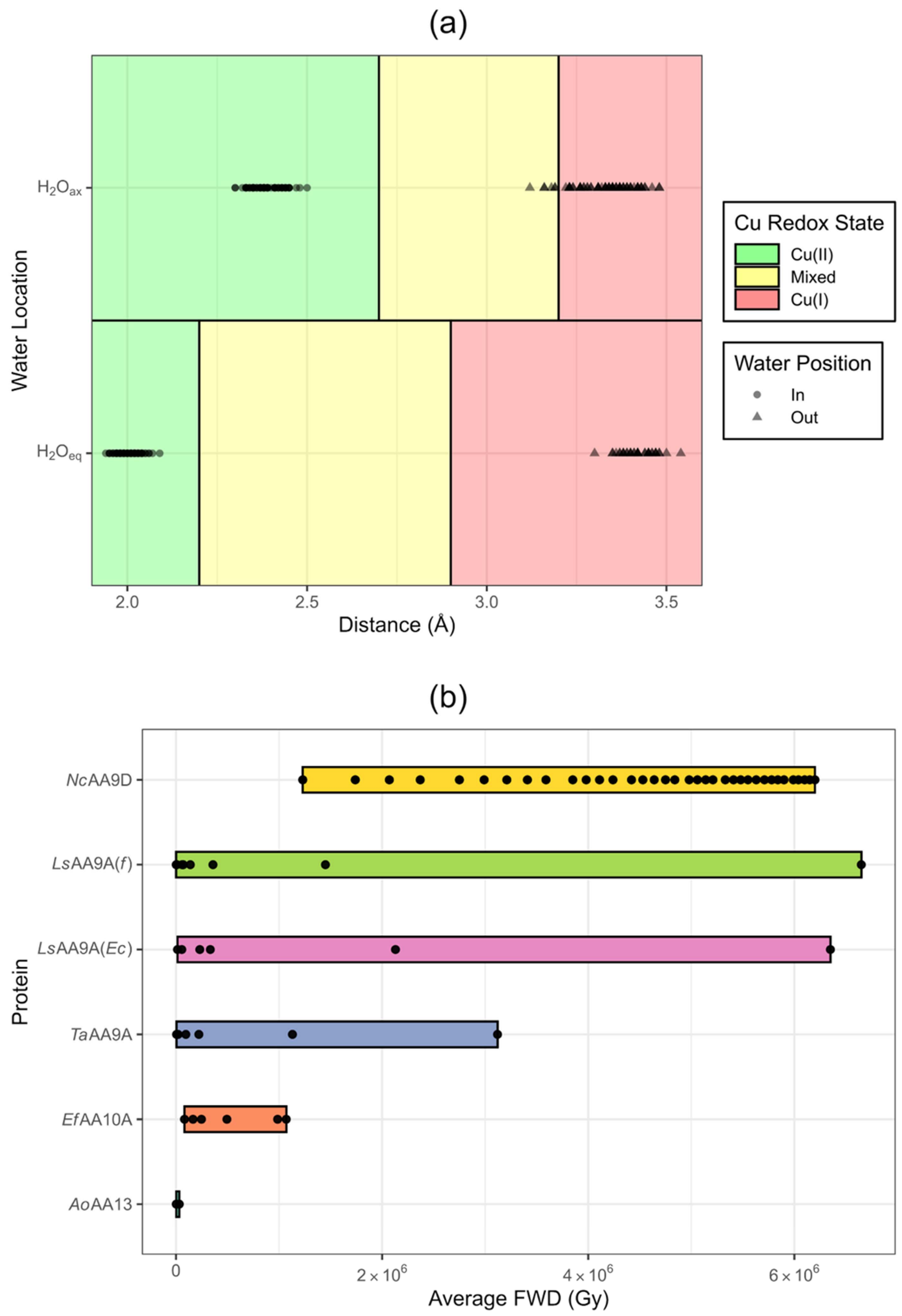

Figure S10 Comparison of LPMO radiation damage studies in the literature. (a) Comparison of average FWD across LPMO radiation damage studies. Each structure's dose is marked by a point, and the span of doses covered by an enzyme is represented by a rectangle. (b) Comparison of Cu–H_2O distances measured in this study to the distances defined by Tandrup *et al.* (2022) for different copper redox states. Waters in the "in" and "out" positions correspond well to the Cu(II)- and Cu(I)-states, respectively. Note that because of the hole-burning observed in our datasets, we caution against the direct comparison of FWD values to those from other studies.

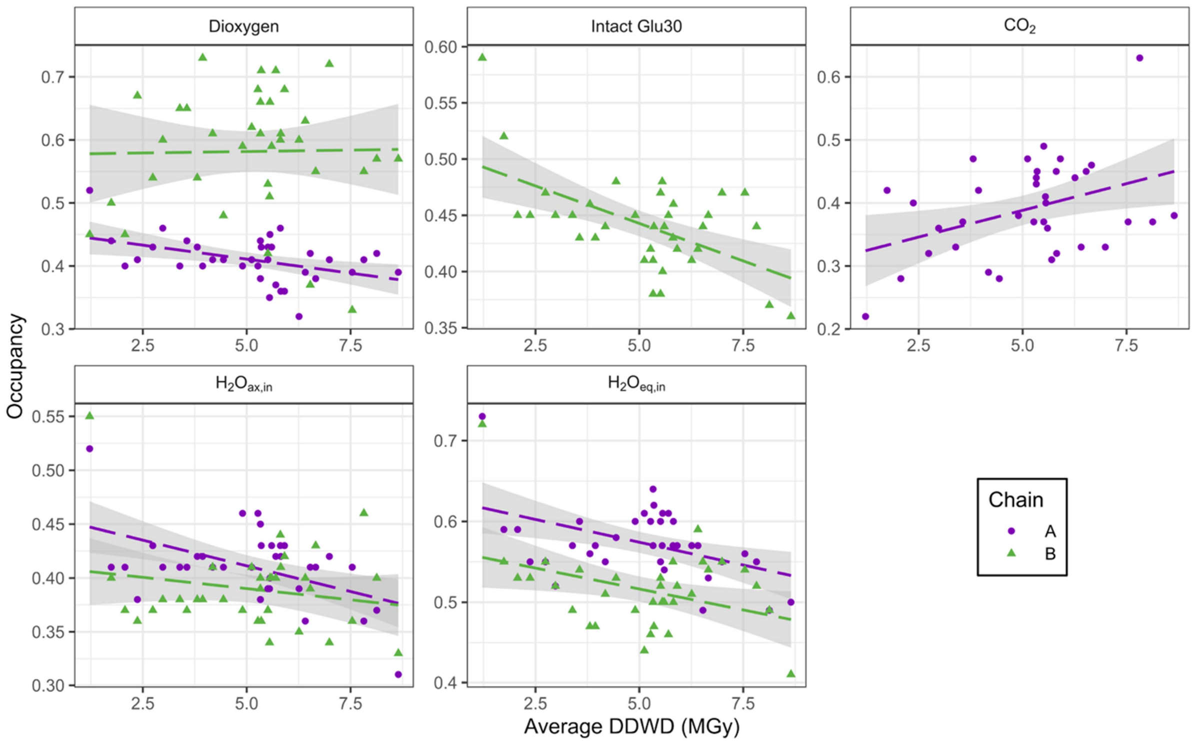

Figure S11 Scatterplot of occupancies versus dose for chain A (purple circles) and chain B (green triangles). Calculated trends for each chain are represented as a dashed line with a 95% confidence interval shaded in gray.

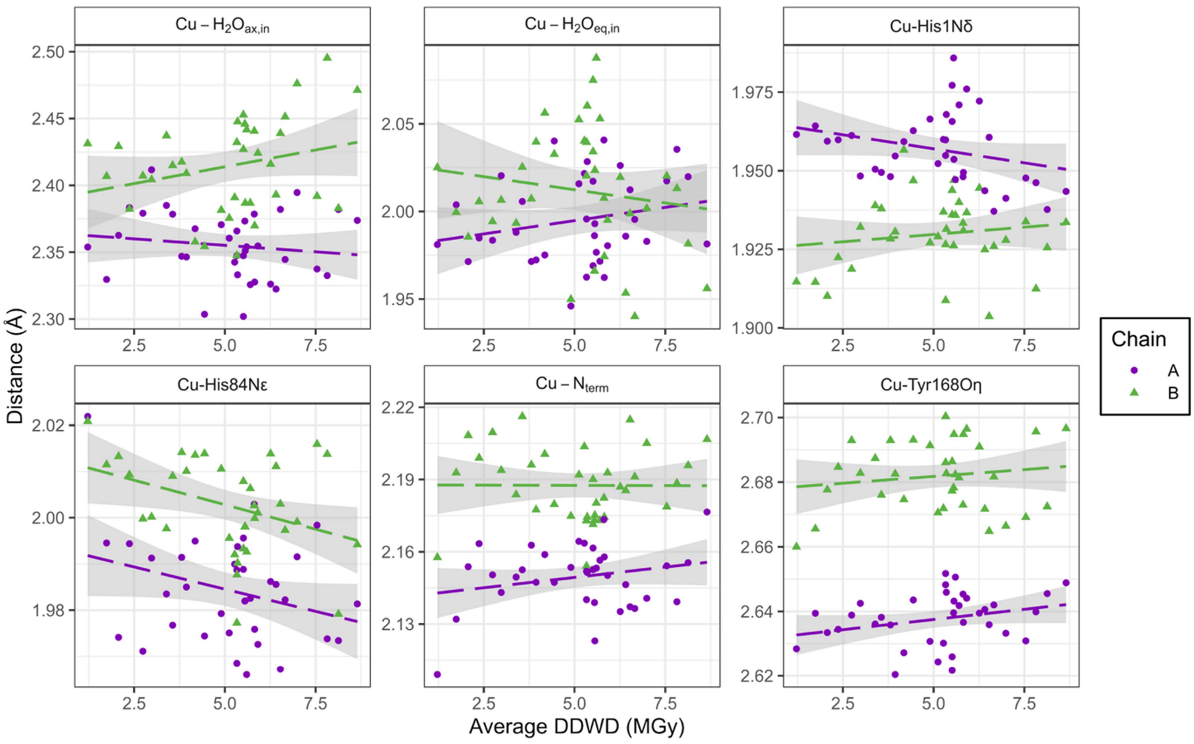

Figure S12 Scatterplot of Cu–ligand distances versus dose for chain A (purple circles) and chain B (green triangles). Calculated trends for each chain are represented as a dashed line with a 95% confidence interval shaded in gray.

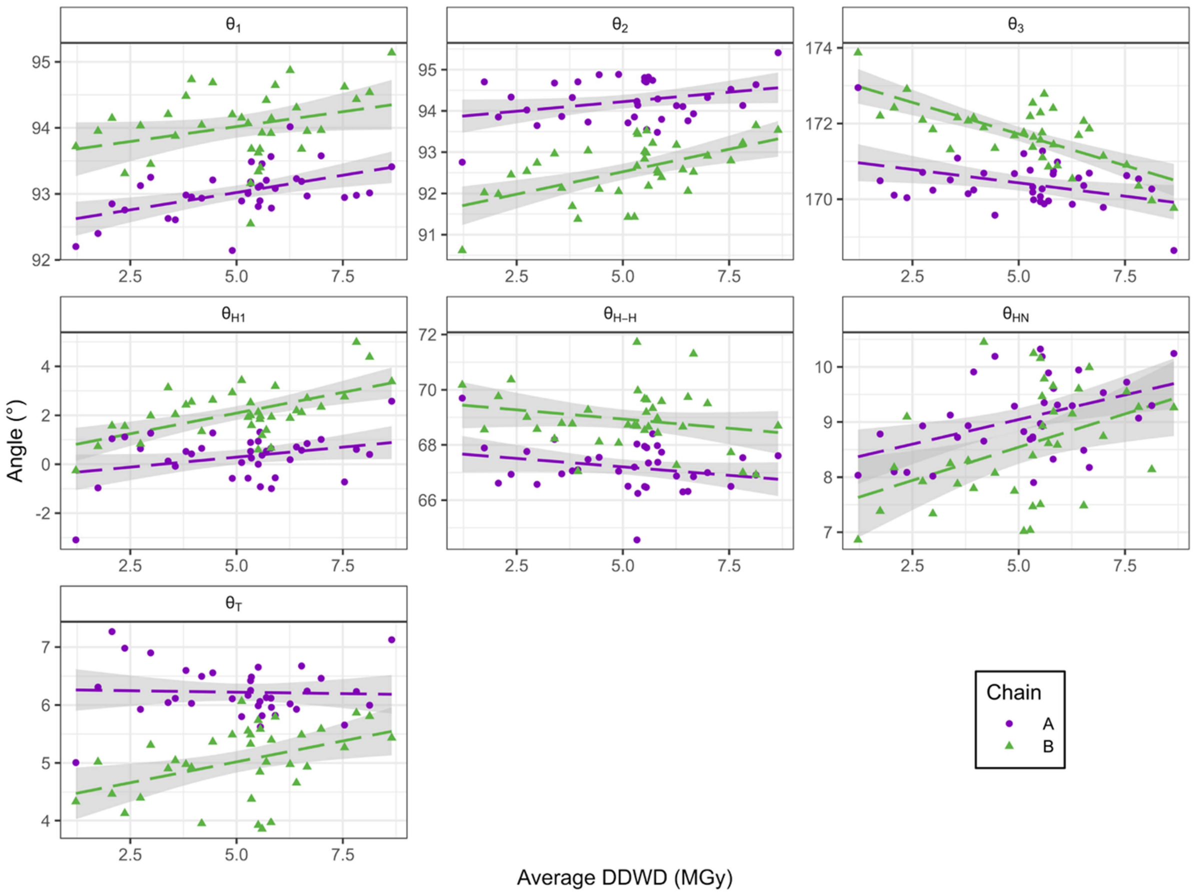

Figure S13 Scatterplot of active site angles versus dose for chain A (purple circles) and chain B (green triangles). Calculated trends for each chain are represented as a dashed line with a 95% confidence interval shaded in gray.

Table S1 Data collection, processing, and refinement statistics for pseudohelices 1–6.

Values in parentheses are for the outer shell.

	Pseudohelix 1	Pseudohelix 2	Pseudohelix 3	Pseudohelix 4	Pseudohelix 5	Pseudohelix 6
**X-ray diffraction**						
φ range	5–185°	6–186°	7–187°	8–188°	9–189°	10–190°
Dose (MGy)	1.22	1.74	2.07	2.37	2.74	2.98
Wavelength (Å)	1	1	1	1	1	1
Resolution range (Å)	44.54–1.10 (1.11–1.10)	44.54–1.10 (1.11–1.10)	44.54–1.10 (1.11–1.10)	44.54–1.10 (1.11–1.10)	44.54–1.10 (1.11–1.10)	44.54–1.10 (1.11–1.10)
Space group	P1 2_1 1	P1 2_1 1	P1 2_1 1	P1 2_1 1	P1 2_1 1	P1 2_1 1
a, b, c (Å)	67.559, 42.21, 69.553	67.566, 42.213, 69.557	67.566, 42.212, 69.554	67.566, 42.214, 69.557	67.569, 42.219, 69.56	67.572, 42.220, 69.558
α, β, γ (°)	90, 98.94, 90	90, 98.94, 90	90, 98.94, 90	90, 98.94, 90	90, 98.94, 90	90, 98.95, 90
Total reflections	540709 (20456)	540405 (20400)	540977 (20431)	541054 (20399)	542034 (20475)	541601 (20430)
Unique reflections	287526 (10263)	287740 (10285)	287785 (10239)	287847 (10283)	288167 (10285)	288174 (10249)
Multiplicity	3.6 (3.8)	3.6 (3.8)	3.6 (3.8)	3.6 (3.8)	3.6 (3.8)	3.6 (3.8)
Completeness (%)	93.26 (89.58)	93.31 (89.71)	93.33 (89.33)	93.34 (89.63)	93.43 (89.57)	93.43 (89.25)
Average I/σ(I)	5.23 (0.94)	5.31 (0.95)	5.41 (0.94)	5.45 (0.98)	5.32 (0.92)	5.30 (0.91)
Wilson B-factor	9.32	9.36	9.41	9.43	9.42	9.49
R_{merge}	0.1238 (0.3662)	0.1219 (0.3505)	0.1202 (0.3567)	0.12 (0.3529)	0.1212 (0.3469)	0.1205 (0.3465)
R_{meas}	0.1539 (0.4247)	0.1517 (0.4072)	0.1496 (0.4142)	0.1494 (0.4098)	0.1506 (0.4028)	0.1498 (0.4024)
R_{pim}	0.08978 (0.214)	0.08861 (0.206)	0.08729 (0.2093)	0.08737 (0.207)	0.0877 (0.2035)	0.08737 (0.2035)
$CC_{1/2}$	0.954 (0.814)	0.952 (0.811)	0.952 (0.804)	0.951 (0.815)	0.951 (0.821)	0.951 (0.828)
CC*	0.988 (0.947)	0.988 (0.947)	0.988 (0.944)	0.987 (0.948)	0.987 (0.95)	0.987 (0.952)
**Refinement**						
Working set reflections	287526 (10263)	287740 (10285)	287785 (10239)	287847 (10283)	288167 (10285)	288174 (10249)
Test set reflections	3799 (135)	3788 (138)	3818 (145)	3820 (131)	3813 (150)	3822 (155)
R_{work}	0.1843 (0.2894)	0.1790 (0.2724)	0.1790 (0.2839)	0.1800 (0.2837)	0.1826 (0.2857)	0.1812 (0.2938)
R_{free}	0.1975 (0.3299)	0.2041 (0.2966)	0.2045 (0.3165)	0.1995 (0.3209)	0.2085 (0.2983)	0.2014 (0.3382)
No. of non-H atoms	5101	5101	5101	5101	5101	5101
Macromolecules	3860	3860	3860	3860	3860	3860
Ligands	131	131	131	131	131	131
Solvent	1110	1110	1110	1110	1110	1110
Protein residues	446	446	446	446	446	446
RMSD - Bonds (Å)	0.003	0.005	0.003	0.004	0.004	0.005
RMSD - Angles (°)	0.81	0.85	0.8	0.84	0.84	0.88
Ramachandran Favored (%)	95.7	95.48	95.93	95.7	95.93	96.15
Ramachandran Allowed (%)	3.85	4.07	3.62	3.85	3.62	3.39
Ramachandran Outliers (%)	0.45	0.45	0.45	0.45	0.45	0.45
Rotamer outliers (%)	1.62	1.39	1.62	1.62	1.39	1.39
Clashscore	2.53	1.39	1.39	1.77	1.65	1.64
Average B-factor	15.67	15.77	15.61	15.7	15.63	15.79
Macromolecules	12.59	12.67	12.6	12.68	12.56	12.67
Ligands	28.16	27.88	26.29	26.73	26.25	29.32
Solvent	24.93	25.13	24.79	24.92	25.05	25.07

Table S2 Data collection, processing, and refinement statistics for pseudohelices 7–12.

Values in parentheses are for the outer shell.

	**Pseudohelix 7**	**Pseudohelix 8**	**Pseudohelix 9**	**Pseudohelix 10**	**Pseudohelix 11**	**Pseudohelix 12**
**X-ray diffraction**						
φ range	12–192°	13–193°	14–194°	15–195°	17–197°	19–199°
Dose (MGy)	3.39	3.56	3.81	3.94	4.18	4.44
Wavelength (Å)	1	1	1	1	1	1
Resolution range (Å)	44.54–1.10 (1.11–1.10)	44.54–1.10 (1.11–1.10)	44.54–1.10 (1.11–1.10)	44.54–1.10 (1.11–1.10)	44.53–1.10 (1.11–1.10)	44.53–1.10 (1.11–1.10)
Space group	$P1\ 2_1\ 1$	$P1\ 2_1\ 1$	$P1\ 2_1\ 1$	$P1\ 2_1\ 1$	$P1\ 2_1\ 1$	$P1\ 2_1\ 1$
a, *b*, *c* (Å)	67.57, 42.218, 69.555	67.572, 42.220, 69.557	67.568, 42.218, 69.552	67.569, 42.221, 69.557	67.567, 42.221, 69.559	67.566, 42.220, 69.551
α, *β*, *γ* (°)	90, 98.95, 90	90, 98.95, 90	90, 98.95, 90	90, 98.95, 90	90, 98.96, 90	90, 98.97, 90
Total reflections	542990 (19697)	542326 (20392)	542780 (19714)	543409 (20453)	543476 (20447)	543286 (20466)
Unique reflections	288389 (9908)	288420 (10257)	288516 (9911)	288741 (10289)	288818 (10270)	288905 (10297)
Multiplicity	3.6 (3.8)	3.6 (3.8)	3.6 (3.8)	3.6 (3.8)	3.6 (3.8)	3.6 (3.8)
Completeness (%)	93.50 (89.59)	93.50 (89.31)	93.55 (89.62)	93.62 (89.58)	93.64 (89.50)	93.69 (89.69)
Average I/σ(I)	5.31 (0.88)	5.27 (0.90)	5.23 (0.88)	5.28 (0.90)	5.23 (0.88)	5.20 (0.87)
Wilson B-factor	9.51	9.48	9.54	9.57	9.68	9.55
R_{merge}	0.1191 (0.3414)	0.1208 (0.3405)	0.1206 (0.3491)	0.1192 (0.3446)	0.12 (0.3469)	0.1206 (0.3499)
R_{meas}	0.1482 (0.3963)	0.15 (0.3954)	0.1499 (0.4051)	0.148 (0.4002)	0.149 (0.4028)	0.1496 (0.4056)
R_{pim}	0.08653 (0.2002)	0.08737 (0.1998)	0.08736 (0.2043)	0.08612 (0.2022)	0.08668 (0.2034)	0.08686 (0.2041)
$CC_{1/2}$	0.953 (0.85)	0.956 (0.856)	0.957 (0.837)	0.957 (0.845)	0.956 (0.845)	0.96 (0.849)
CC*	0.988 (0.959)	0.989 (0.96)	0.989 (0.955)	0.989 (0.957)	0.989 (0.957)	0.99 (0.958)
**Refinement**						
Working set reflections	288389 (9908)	288420 (10257)	288516 (9911)	288741 (10289)	288818 (10270)	288905 (10297)
Test set reflections	3851 (126)	3789 (134)	3865 (138)	3796 (130)	3847 (146)	3822 (145)
R_{work}	0.1789 (0.2829)	0.1783 (0.2795)	0.1752 (0.2813)	0.1796 (0.2812)	0.1782 (0.2759)	0.1760 (0.2959)
R_{free}	0.2061 (0.3205)	0.2018 (0.3090)	0.2033 (0.3222)	0.2047 (0.2979)	0.2042 (0.2948)	0.2081 (0.3622)
No. of non-H atoms	5101	5101	5101	5101	5101	5101
Macromolecules	3860	3860	3860	3860	3860	3860
Ligands	131	131	131	131	131	131
Solvent	1110	1110	1110	1110	1110	1110
Protein residues	446	446	446	446	446	446
RMSD - Bonds (Å)	0.007	0.005	0.01	0.006	0.004	0.008
RMSD - Angles (°)	1.07	0.87	1.12	0.91	0.88	1
Ramachandran Favored (%)	96.38	95.7	96.38	95.7	95.93	96.38
Ramachandran Allowed (%)	3.39	3.85	3.17	3.85	3.62	3.17
Ramachandran Outliers (%)	0.23	0.45	0.45	0.45	0.45	0.45
Rotamer outliers (%)	1.39	1.62	1.62	1.62	1.39	1.62
Clashscore	3.92	1.52	2.15	1.64	2.28	1.9
Average B-factor	15.74	15.9	15.83	15.95	16.15	15.71
Macromolecules	12.68	12.8	12.78	12.83	13.03	12.69
Ligands	26.83	27.6	26.7	29.14	26.9	26.62
Solvent	25.09	25.3	25.16	25.23	25.76	24.94

Table S3 Data collection, processing, and refinement statistics for pseudohelices 13–18.

Values in parentheses are for the outer shell.

	**Pseudohelix 13**	**Pseudohelix 14**	**Pseudohelix 15**	**Pseudohelix 16**	**Pseudohelix 17**	**Pseudohelix 18**
**X-ray diffraction**						
φ range	24–204°	27–207°	30–210°	31–211°	36–216°	41–221°
Dose (MGy)	4.90	5.12	5.27	5.33	5.56	5.70
Wavelength (Å)	1	1	1	1	1	1
Resolution range (Å)	44.52–1.10 (1.11–1.10)	44.52–1.10 (1.11–1.10)	44.52–1.10 (1.11–1.10)	44.53–1.10 (1.11–1.10)	44.53–1.10 (1.11–1.10)	44.52–1.10 (1.11–1.10)
Space group	$P1\ 2_1\ 1$	$P1\ 2_1\ 1$	$P1\ 2_1\ 1$	$P1\ 2_1\ 1$	$P1\ 2_1\ 1$	$P1\ 2_1\ 1$
a, b, c (Å)	67.558, 42.228, 69.558	67.554, 42.225, 69.557	67.556, 42.225, 69.564	67.562, 42.224, 69.564	67.551, 42.228, 69.562	67.539, 42.228, 69.568
α, β, γ (°)	90, 98.98, 90	90, 98.98, 90	90, 98.98, 90	90, 98.96, 90	90, 98.97, 90	90, 98.97, 90
Total reflections	542633 (20439)	543358 (20375)	543242 (20435)	543297 (20453)	544932 (20424)	544250 (20399)
Unique reflections	289242 (10284)	288981 (10228)	289492 (10309)	289601 (10327)	289779 (10308)	289947 (10312)
Multiplicity	3.6 (3.8)	3.6 (3.8)	3.6 (3.8)	3.6 (3.8)	3.6 (3.8)	3.6 (3.8)
Completeness (%)	93.78 (89.66)	93.71 (89.22)	93.86 (89.85)	93.89 (89.93)	93.95 (89.90)	94.02 (89.89)
Average I/σ(I)	5.26 (0.87)	5.22 (0.84)	5.28 (0.87)	5.32 (0.88)	5.37 (0.85)	5.30 (0.83)
Wilson B-factor	9.62	9.68	9.55	9.57	9.65	9.68
R_{merge}	0.1179 (0.346)	0.1179 (0.3483)	0.1177 (0.3365)	0.1178 (0.3376)	0.1137 (0.3296)	0.115 (0.3358)
R_{meas}	0.1462 (0.4016)	0.1462 (0.4043)	0.146 (0.3903)	0.1461 (0.3923)	0.1409 (0.3825)	0.1425 (0.3895)
R_{pim}	0.08482 (0.2026)	0.08493 (0.2042)	0.0848 (0.1967)	0.0848 (0.1986)	0.08156 (0.193)	0.08268 (0.1963)
$CC_{1/2}$	0.951 (0.822)	0.962 (0.836)	0.958 (0.851)	0.957 (0.826)	0.966 (0.877)	0.963 (0.857)
CC*	0.987 (0.95)	0.99 (0.954)	0.989 (0.959)	0.989 (0.951)	0.991 (0.967)	0.991 (0.961)
**Refinement**						
Working set reflections	289242 (10284)	288981 (10228)	289492 (10309)	289601 (10327)	289779 (10308)	289947 (10312)
Test set reflections	3778 (132)	3807 (150)	3821 (138)	3821 (143)	3848 (142)	3834 (123)
R_{work}	0.1760 (0.2836)	0.1743 (0.2870)	0.1745 (0.2833)	0.1768 (0.2801)	0.1704 (0.2835)	0.1725 (0.2900)
R_{free}	0.2082 (0.2994)	0.1999 (0.3148)	0.1962 (0.3078)	0.1997 (0.2945)	0.1910 (0.3361)	0.2015 (0.2782)
No. of non-H atoms	5101	5101	5101	5101	5101	5101
Macromolecules	3860	3860	3860	3860	3860	3860
Ligands	131	131	131	131	131	131
Solvent	1110	1110	1110	1110	1110	1110
Protein residues	446	446	446	446	446	446
RMSD - Bonds (Å)	0.003	0.007	0.005	0.003	0.009	0.005
RMSD - Angles (°)	0.81	0.99	0.93	0.78	1.09	0.91
Ramachandran Favored (%)	96.15	96.15	95.93	95.7	96.15	96.38
Ramachandran Allowed (%)	3.39	3.39	3.62	3.85	3.39	3.39
Ramachandran Outliers (%)	0.45	0.45	0.45	0.45	0.45	0.23
Rotamer outliers (%)	1.62	1.62	1.62	1.39	1.85	1.39
Clashscore	1.77	2.52	3.78	1.65	2.91	1.52
Average B-factor	16.05	15.94	15.93	15.83	16.05	15.84
Macromolecules	12.96	12.9	12.85	12.8	12.97	12.82
Ligands	27.21	27.47	28.46	26.54	28.32	27.13
Solvent	25.47	25.17	25.15	25.1	25.33	25.01

Table S4 Data collection, processing, and refinement statistics for pseudohelices 19–24.

Values in parentheses are for the outer shell.

	**Pseudohelix 19**	**Pseudohelix 20**	**Pseudohelix 21**	**Pseudohelix 22**	**Pseudohelix 23**	**Pseudohelix 24**
**X-ray diffraction**						
φ range	55–235°	80–260°	81–261°	96–276°	105–285°	122–302°
Dose (MGy)	5.91	5.82	5.81	5.60	5.51	5.35
Wavelength (Å)	1	1	1	1	1	1
Resolution range (Å)	44.51–1.10 (1.11–1.10)	34.35–1.10 (1.11–1.10)	34.35–1.10 (1.11–1.10)	44.49–1.10 (1.11–1.10)	44.50–1.10 (1.11–1.10)	44.51–1.10 (1.11–1.10)
Space group	$P1\ 2_1\ 1$	$P1\ 2_1\ 1$	$P1\ 2_1\ 1$	$P1\ 2_1\ 1$	$P1\ 2_1\ 1$	$P1\ 2_1\ 1$
a, b, c (Å)	67.522, 42.234, 69.580	67.510, 42.22, 69.551	67.512, 42.223, 69.552	67.492, 42.222, 69.556	67.484, 42.231, 69.573	67.485, 42.225, 69.587
α, β, γ (°)	90, 98.99, 90	90, 99.00, 90	90, 99.01, 90	90, 98.99, 90	90, 98.98, 90	90, 98.96, 90
Total reflections	545223 (19735)	544173 (20380)	544991 (19587)	545079 (20394)	545164 (20323)	544162 (20313)
Unique reflections	290096 (10017)	289006 (10329)	288960 (9915)	287673 (10256)	286997 (10171)	285679 (10118)
Multiplicity	3.6 (3.8)	3.6 (3.8)	3.6 (3.8)	3.6 (3.8)	3.6 (3.8)	3.6 (3.8)
Completeness (%)	94.06 (90.27)	93.80 (90.01)	93.77 (89.92)	93.38 (89.40)	93.12 (88.89)	92.69 (88.47)
Average I/σ(I)	5.32 (0.85)	5.78 (1.07)	5.75 (1.03)	5.75 (1.03)	5.79 (1.00)	5.78 (1.04)
Wilson B-factor	9.7	9.41	9.46	9.51	9.52	9.5
R_{merge}	0.114 (0.3285)	0.1132 (0.3191)	0.1116 (0.3139)	0.1134 (0.3267)	0.1135 (0.3356)	0.1131 (0.3269)
R_{meas}	0.1411 (0.3815)	0.1401 (0.371)	0.1382 (0.3646)	0.1407 (0.3795)	0.141 (0.3894)	0.1405 (0.3797)
R_{pim}	0.08168 (0.1927)	0.08111 (0.1881)	0.08003 (0.1844)	0.0818 (0.1919)	0.08224 (0.1965)	0.08184 (0.1922)
$CC_{1/2}$	0.965 (0.861)	0.961 (0.829)	0.965 (0.864)	0.965 (0.848)	0.956 (0.847)	0.964 (0.856)
CC*	0.991 (0.962)	0.99 (0.952)	0.991 (0.963)	0.991 (0.958)	0.989 (0.958)	0.991 (0.96)
**Refinement**						
Working set reflections	290096 (10017)	289006 (10329)	288960 (9915)	287673 (10256)	286997 (10171)	285679 (10118)
Test set reflections	3852 (122)	3814 (138)	3853 (142)	3803 (146)	3808 (125)	3795 (127)
R_{work}	0.1721 (0.2873)	0.1689 (0.2622)	0.1671 (0.2612)	0.1718 (0.2823)	0.1739 (0.2719)	0.1749 (0.2708)
R_{free}	0.1933 (0.2922)	0.1918 (0.3326)	0.1967 (0.2823)	0.1875 (0.3389)	0.1981 (0.2949)	0.2133 (0.2991)
No. of non-H atoms	5101	5101	5101	5101	5101	5101
Macromolecules	3860	3860	3860	3860	3860	3860
Ligands	131	131	131	131	131	131
Solvent	1110	1110	1110	1110	1110	1110
Protein residues	446	446	446	446	446	446
RMSD - Bonds (Å)	0.008	0.008	0.01	0.008	0.004	0.003
RMSD - Angles (°)	1.01	1.02	1.14	1.03	0.91	0.79
Ramachandran Favored (%)	96.15	95.48	96.38	95.93	96.15	95.93
Ramachandran Allowed (%)	3.39	4.07	3.17	3.62	3.39	3.62
Ramachandran Outliers (%)	0.45	0.45	0.45	0.45	0.45	0.45
Rotamer outliers (%)	1.85	1.85	1.85	1.85	1.62	1.39
Clashscore	2.78	1.9	3.04	3.29	3.29	1.26
Average B-factor	16.31	15.8	15.69	15.59	15.86	16.03
Macromolecules	13.16	12.73	12.63	12.53	12.72	12.92
Ligands	29.41	26.98	27.08	26.42	28.85	28.47
Solvent	25.74	25.15	25.01	24.96	25.25	25.39

Table S5 Data collection, processing, and refinement statistics for pseudohelices 25–30.

Values in parentheses are for the outer shell.

	Pseudohelix 25	Pseudohelix 26	Pseudohelix 27	Pseudohelix 28	Pseudohelix 29	Pseudohelix 30
**X-ray diffraction**						
φ range	130–310°	153–333°	155–335°	170–350°	171–351°	172–352°
Dose (MGy)	5.33	5.51	5.55	6.26	6.41	6.53
Wavelength (Å)	1	1	1	1	1	1
Resolution range (Å)	44.52–1.10 (1.11–1.10)	44.54–1.10 (1.11–1.10)	44.54–1.10 (1.11–1.10)	44.52–1.10 (1.11–1.10)	44.52–1.10 (1.11–1.10)	44.52–1.10 (1.11–1.10)
Space group	$P1\ 2_1\ 1$	$P1\ 2_1\ 1$	$P1\ 2_1\ 1$	$P1\ 2_1\ 1$	$P1\ 2_1\ 1$	$P1\ 2_1\ 1$
a, b, c (Å)	67.489, 42.226, 69.575	67.513, 42.224, 69.572	67.513, 42.223, 69.569	67.530, 42.213, 69.532	67.537, 42.217, 69.530	67.533, 42.211, 69.528
α, β, γ (°)	90, 98.95, 90	90, 98.92, 90	90, 98.92, 90	90, 98.95, 90	90, 98.94, 90	90, 98.95, 90
Total reflections	543691 (20323)	543292 (20366)	542698 (20397)	542929 (20393)	543393 (20414)	543295 (20451)
Unique reflections	285102 (10092)	285255 (10139)	285381 (10152)	286580 (10197)	286637 (10184)	286698 (10191)
Multiplicity	3.6 (3.8)	3.6 (3.8)	3.6 (3.8)	3.6 (3.8)	3.6 (3.8)	3.6 (3.8)
Completeness (%)	92.51 (88.19)	92.52 (88.26)	92.57 (88.40)	93.01 (88.77)	93.01 (88.60)	93.05 (88.68)
Average I/σ(I)	5.69 (1.01)	5.58 (0.97)	5.45 (0.93)	5.43 (0.91)	5.39 (0.89)	5.42 (0.88)
Wilson B-factor	9.56	9.62	9.63	9.74	9.77	9.81
R_{merge}	0.114 (0.3301)	0.1168 (0.3346)	0.1191 (0.337)	0.1177 (0.3472)	0.1188 (0.3499)	0.1179 (0.3521)
R_{meas}	0.1417 (0.3834)	0.1453 (0.3889)	0.1483 (0.3912)	0.1462 (0.4029)	0.1475 (0.4061)	0.1463 (0.4085)
R_{pim}	0.08263 (0.194)	0.08498 (0.1971)	0.08672 (0.1978)	0.08514 (0.2035)	0.08583 (0.205)	0.08518 (0.206)
$CC_{1/2}$	0.955 (0.844)	0.953 (0.835)	0.953 (0.851)	0.962 (0.847)	0.958 (0.843)	0.957 (0.851)
CC*	0.988 (0.957)	0.988 (0.954)	0.988 (0.959)	0.99 (0.958)	0.989 (0.956)	0.989 (0.959)
**Refinement**						
Working set reflections	285102 (10092)	285255 (10139)	285381 (10152)	286580 (10197)	286637 (10184)	286698 (10191)
Test set reflections	3796 (140)	3773 (143)	3757 (124)	3788 (156)	3773 (136)	3781 (136)
R_{work}	0.1738 (0.2691)	0.1757 (0.2575)	0.1757 (0.2653)	0.1729 (0.2847)	0.1801 (0.2860)	0.1775 (0.2920)
R_{free}	0.2004 (0.2857)	0.1949 (0.2492)	0.1982 (0.2791)	0.1914 (0.3176)	0.2101 (0.3188)	0.2020 (0.2968)
No. of non-H atoms	5101	5101	5101	5101	5101	5101
Macromolecules	3860	3860	3860	3860	3860	3860
Ligands	131	131	131	131	131	131
Solvent	1110	1110	1110	1110	1110	1110
Protein residues	446	446	446	446	446	446
RMSD - Bonds (Å)	0.007	0.005	0.006	0.011	0.005	0.01
RMSD - Angles (°)	1.05	0.91	0.94	1.17	0.86	1.18
Ramachandran Favored (%)	96.38	95.93	96.38	96.61	96.38	96.83
Ramachandran Allowed (%)	3.17	3.62	3.17	3.17	3.39	2.94
Ramachandran Outliers (%)	0.45	0.45	0.45	0.23	0.23	0.23
Rotamer outliers (%)	1.62	1.62	1.39	1.39	1.39	1.85
Clashscore	3.93	2.15	2.28	3.93	1.65	3.8
Average B-factor	16.09	16.14	16.3	16.11	16.28	16.21
Macromolecules	12.95	12.98	13.14	13.03	13.19	13.09
Ligands	29	29.78	29.29	28.04	27.58	27.49
Solvent	25.46	25.52	25.76	25.41	25.68	25.72

Table S6 Data collection, processing, and refinement statistics for pseudohelices 31–36.

Values in parentheses are for the outer shell.

	Pseudohelix 31	Pseudohelix 32	Pseudohelix 33	Pseudohelix 34	Pseudohelix 35	Pseudohelix 36
**X-ray diffraction**						
φ range	173–353°	175–355°	177–357°	178–358°	179–359°	180–360°
Dose (MGy)	6.66	6.99	7.54	7.82	8.13	8.65
Wavelength (Å)	1	1	1	1	1	1
Resolution range (Å)	44.52–1.10 (1.11–1.10)	44.52–1.10 (1.11–1.10)	44.52–1.10 (1.111.10)	44.52–1.10 (1.11–1.10)	44.52–1.10 (1.11–1.10)	44.52–1.10 (1.11–1.10)
Space group	$P1\ 2_1\ 1$	$P1\ 2_1\ 1$	$P1\ 2_1\ 1$	$P1\ 2_1\ 1$	$P1\ 2_1\ 1$	$P1\ 2_1\ 1$
a, b, c (Å)	67.539, 42.216, 69.528	67.540, 42.212, 69.523	67.547, 42.221, 69.518	67.543, 42.220, 69.516	67.543, 42.219, 69.515	67.544, 42.223, 69.519
α, β, γ (°)	90, 98.95, 90	90, 98.95, 90	90, 98.94, 90	90, 98.94, 90	90, 98.94, 90	90, 98.94, 90
Total reflections	543724 (20443)	543343 (20430)	544098 (20439)	543300 (20395)	544006 (20396)	546117 (20479)
Unique reflections	286860 (10207)	287011 (10222)	287104 (10218)	287029 (10186)	287195 (10173)	287461 (10193)
Multiplicity	3.6 (3.8)	3.6 (3.8)	3.6 (3.8)	3.6 (3.8)	3.6 (3.8)	3.6 (3.8)
Completeness (%)	93.08 (88.82)	93.15 (88.90)	93.16 (88.93)	93.14 (88.70)	93.21 (88.67)	93.27 (88.75)
Average I/σ(I)	5.37 (0.86)	5.45 (0.88)	5.32 (0.84)	5.32 (0.84)	5.31 (0.83)	5.22 (0.81)
Wilson B-factor	9.83	9.84	9.89	9.96	9.86	9.99
R_{merge}	0.1178 (0.3509)	0.1162 (0.3467)	0.1173 (0.3542)	0.1174 (0.3574)	0.1192 (0.366)	0.1198 (0.3638)
R_{meas}	0.1464 (0.4073)	0.1444 (0.4024)	0.1457 (0.4113)	0.1459 (0.4152)	0.1483 (0.4256)	0.1488 (0.4227)
R_{pim}	0.08533 (0.2057)	0.08413 (0.2032)	0.08484 (0.2078)	0.08506 (0.2101)	0.08652 (0.2157)	0.08657 (0.2137)
$CC_{1/2}$	0.961 (0.85)	0.954 (0.839)	0.958 (0.84)	0.961 (0.848)	0.946 (0.646)	0.953 (0.767)
CC*	0.99 (0.958)	0.988 (0.955)	0.989 (0.956)	0.99 (0.958)	0.986 (0.886)	0.988 (0.932)
**Refinement**						
Working set reflections	286860 (10207)	287011 (10222)	287104 (10218)	287029 (10186)	287195 (10173)	287461 (10193)
Test set reflections	3813 (132)	3803 (138)	3785 (136)	3790 (147)	3807 (141)	3806 (128)
R_{work}	0.1793 (0.2922)	0.1801 (0.2963)	0.1800 (0.2890)	0.1778 (0.2867)	0.1777 (0.2936)	0.1794 (0.2947)
R_{free}	0.2096 (0.3314)	0.2084 (0.2517)	0.2055 (0.3161)	0.2117 (0.3021)	0.2017 (0.3116)	0.2054 (0.3300)
No. of non-H atoms	5101	5101	5101	5101	5101	5101
Macromolecules	3860	3860	3860	3860	3860	3860
Ligands	131	131	131	131	131	131
Solvent	1110	1110	1110	1110	1110	1110
Protein residues	446	446	446	446	446	446
RMSD - Bonds (Å)	0.004	0.003	0.005	0.006	0.005	0.005
RMSD - Angles (°)	0.81	0.8	0.89	0.93	0.92	0.94
Ramachandran Favored (%)	95.93	96.38	95.93	96.38	96.15	96.15
Ramachandran Allowed (%)	3.62	3.17	3.62	3.17	3.39	3.39
Ramachandran Outliers (%)	0.45	0.45	0.45	0.45	0.45	0.45
Rotamer outliers (%)	1.39	1.39	1.62	1.62	1.62	1.62
Clashscore	1.26	1.77	2.41	2.28	1.77	2.91
Average B-factor	16.38	16.21	16.33	16.39	16.28	16.31
Macromolecules	13.23	13.05	13.16	13.33	13.16	13.21
Ligands	29.39	29.02	28.39	27.17	27.76	28.4
Solvent	25.81	25.71	25.94	25.77	25.77	25.66

Table S7 Data collection, processing, and refinement statistics for wedges 1–6.

Values in parentheses are for the outer shell.

	Wedge 1	Wedge 2	Wedge 3	Wedge 4	Wedge 5	Wedge 6
**X-ray diffraction**						
φ range	5–185°	10–190°	15–195°	20–200°	25–205°	30–210°
Dose (MGy)	5.45	5.48	5.48	5.49	5.49	5.50
Wavelength (Å)	1	1	1	1	1	1
Resolution range (Å)	44.51–1.10 (1.11–1.10)	44.50–1.10 (1.11–1.10)	44.50–1.10 (1.11–1.10)	44.49–1.10 (1.11–1.10)	44.49–1.10 (1.11–1.10)	44.49–1.10 (1.11–1.10)
Space group	$P1\ 2_1\ 1$	$P1\ 2_1\ 1$	$P1\ 2_1\ 1$	$P1\ 2_1\ 1$	$P1\ 2_1\ 1$	$P1\ 2_1\ 1$
a, b, c (Å)	67.490, 42.262, 69.521	67.496, 42.263, 69.520	67.498, 42.263, 69.519	67.501, 42.262, 69.518	67.497, 42.262, 69.519	67.490, 42.262, 69.521
α, β, γ (°)	90, 98.94, 90	90, 98.96, 90	90, 98.97, 90	90, 98.97, 90	90, 98.98, 90	90, 98.94, 90
Total reflections	544003 (20223)	542761 (20269)	543280 (20270)	542680 (19588)	542675 (19594)	539781 (20232)
Unique reflections	288405 (10281)	288688 (10327)	289038 (10350)	289263 (10021)	289549 (10035)	289529 (10371)
Multiplicity	3.6 (3.8)	3.6 (3.8)	3.6 (3.8)	3.6 (3.8)	3.6 (3.8)	3.6 (3.8)
Completeness (%)	93.56 (89.53)	93.64 (89.70)	93.75 (89.85)	93.82 (89.83)	93.92 (89.95)	93.91 (90.21)
Average I/σ(I)	6.64 (1.40)	6.52 (1.39)	6.52 (1.36)	6.48 (1.36)	6.37 (1.31)	6.12 (1.30)
Wilson B-factor	9.19	9.2	9.28	9.23	9.24	9.25
R_{merge}	0.1025 (0.2497)	0.1052 (0.2571)	0.1034 (0.2606)	0.106 (0.2615)	0.1046 (0.266)	0.1108 (0.2746)
R_{meas}	0.1272 (0.2904)	0.1307 (0.2987)	0.1284 (0.3027)	0.1319 (0.3039)	0.1301 (0.3092)	0.138 (0.3189)
R_{pim}	0.07396 (0.1477)	0.07628 (0.1514)	0.07469 (0.1533)	0.07705 (0.1541)	0.07591 (0.157)	0.08088 (0.1615)
$CC_{1/2}$	0.961 (0.902)	0.962 (0.902)	0.968 (0.904)	0.954 (0.902)	0.966 (0.896)	0.958 (0.884)
CC*	0.99 (0.974)	0.99 (0.974)	0.992 (0.974)	0.988 (0.974)	0.991 (0.972)	0.989 (0.969)
**Refinement**						
Working set reflections	288405 (10281)	288688 (10327)	289038 (10350)	289263 (10021)	289549 (10035)	289529 (10371)
Test set reflections	3814 (145)	3826 (107)	3798 (143)	3853 (148)	3856 (118)	3813 (134)
R_{work}	0.1647 (0.2234)	0.1651 (0.2358)	0.1627 (0.2350)	0.1658 (0.2324)	0.1669 (0.2410)	0.1713 (0.2409)
R_{free}	0.1842 (0.2329)	0.1918 (0.2762)	0.1841 (0.2542)	0.1946 (0.2740)	0.1932 (0.2746)	0.1962 (0.2413)
No. of non-H atoms	5101	5101	5101	5101	5101	5101
Macromolecules	3860	3860	3860	3860	3860	3860
Ligands	131	131	131	131	131	131
Solvent	1110	1110	1110	1110	1110	1110
Protein residues	446	446	446	446	446	446
RMSD - Bonds (Å)	0.003	0.004	0.009	0.004	0.004	0.004
RMSD - Angles (°)	0.79	0.92	1.1	0.84	0.91	0.82
Ramachandran Favored (%)	96.15	96.38	96.38	95.93	96.15	95.93
Ramachandran Allowed (%)	3.39	3.17	3.17	3.62	3.39	3.62
Ramachandran Outliers (%)	0.45	0.45	0.45	0.45	0.45	0.45
Rotamer outliers (%)	1.62	1.39	1.85	1.39	1.62	1.39
Clashscore	1.14	3.42	3.04	1.52	3.16	1.39
Average B-factor	15.5	15.54	15.68	15.63	15.42	15.51
Macromolecules	12.39	12.4	12.48	12.45	12.36	12.37
Ligands	27.8	28.44	28.89	28.25	26.53	27.6
Solvent	24.87	24.94	25.26	25.21	24.78	24.98

Table S8 Data collection, processing, and refinement statistics for wedges 7–12.

Values in parentheses are for the outer shell.

	Wedge 7	Wedge 8	Wedge 9	Wedge 10	Wedge 11	Wedge 12
**X-ray diffraction**						
φ range	35–215°	40–220°	45–225°	50–230°	55–235°	60–240°
Dose (MGy)	5.51	5.48	5.50	5.44	5.44	5.41
Wavelength (Å)	1	1	1	1	1	1
Resolution range (Å)	44.49–1.10 (1.11–1.10)	44.50–1.10 (1.11–1.10)	44.50–1.10 (1.11–1.10)	44.51–1.10 (1.11–1.10)	44.52–1.10 (1.11–1.10)	44.53–1.10 (1.11–1.10)
Space group	$P1\ 2_1\ 1$	$P1\ 2_1\ 1$	$P1\ 2_1\ 1$	$P1\ 2_1\ 1$	$P1\ 2_1\ 1$	$P1\ 2_1\ 1$
a, b, c (Å)	67.499, 42.258, 69.527	67.502, 42.253, 69.542	67.506, 42.239, 69.553	67.510, 42.223, 69.565	67.522, 42.208, 69.582	67.542, 42.193, 69.604
α, β, γ (°)	90, 98.98, 90	90, 98.98, 90	90, 98.98, 90	90, 98.97, 90	90, 98.98, 90	90, 98.98, 90
Total reflections	539796 (20196)	540145 (20226)	540893 (20202)	542181 (19541)	543869 (19500)	545106 (19501)
Unique reflections	289616 (10352)	289877 (10369)	290051 (10361)	290053 (10016)	290480 (10006)	290599 (10002)
Multiplicity	3.6 (3.8)	3.6 (3.8)	3.6 (3.8)	3.6 (3.8)	3.6 (3.8)	3.6 (3.8)
Completeness (%)	93.94 (90.26)	94.02 (90.34)	94.10 (90.42)	94.11 (90.43)	94.24 (90.37)	94.26 (90.41)
Average I/σ(I)	5.89 (1.21)	5.87 (1.18)	5.75 (1.10)	5.62 (0.97)	5.57 (0.89)	5.51 (0.86)
Wilson B-factor	9.42	9.33	9.39	9.52	9.72	9.83
R_{merge}	0.1122 (0.2908)	0.1123 (0.288)	0.1141 (0.2931)	0.1108 (0.2987)	0.1084 (0.2974)	0.1077 (0.2969)
R_{meas}	0.1395 (0.3379)	0.1397 (0.3349)	0.1417 (0.3408)	0.1374 (0.347)	0.1341 (0.3458)	0.1328 (0.3454)
R_{pim}	0.08154 (0.1714)	0.08157 (0.17)	0.08261 (0.173)	0.07984 (0.1759)	0.07753 (0.1756)	0.07629 (0.1755)
$CC_{1/2}$	0.963 (0.885)	0.96 (0.868)	0.954 (0.876)	0.964 (0.884)	0.968 (0.887)	0.966 (0.896)
CC*	0.99 (0.969)	0.99 (0.964)	0.988 (0.966)	0.991 (0.969)	0.992 (0.97)	0.991 (0.972)
**Refinement**						
Working set reflections	289616 (10352)	289877 (10369)	290051 (10361)	290053 (10016)	290480 (10006)	290599 (10002)
Test set reflections	3845 (142)	3825 (155)	3838 (132)	3862 (136)	3887 (143)	3871 (137)
R_{work}	0.1734 (0.2645)	0.1709 (0.2481)	0.1746 (0.2605)	0.1733 (0.2710)	0.1679 (0.2730)	0.1699 (0.2915)
R_{free}	0.1965 (0.2975)	0.1844 (0.2776)	0.2013 (0.2611)	0.1967 (0.2821)	0.1916 (0.2964)	0.1907 (0.2968)
No. of non-H atoms	5101	5101	5101	5101	5101	5101
Macromolecules	3860	3860	3860	3860	3860	3860
Ligands	131	131	131	131	131	131
Solvent	1110	1110	1110	1110	1110	1110
Protein residues	446	446	446	446	446	446
RMSD - Bonds (Å)	0.005	0.004	0.005	0.006	0.008	0.007
RMSD - Angles (°)	0.91	0.9	0.95	0.93	1.01	0.98
Ramachandran Favored (%)	95.93	96.15	96.15	96.15	95.7	96.15
Ramachandran Allowed (%)	3.62	3.39	3.62	3.39	4.07	3.39
Ramachandran Outliers (%)	0.45	0.45	0.23	0.45	0.23	0.45
Rotamer outliers (%)	1.39	1.62	2.08	1.62	1.62	1.85
Clashscore	3.29	2.15	3.17	3.8	1.52	2.66
Average B-factor	15.68	15.54	15.77	16.25	16.45	16.32
Macromolecules	12.52	12.47	12.59	13.07	13.31	13.24
Ligands	27.26	27.62	28.76	30.06	29.05	27.55
Solvent	25.32	24.8	25.33	25.66	25.89	25.69

Table S9 Data collection, processing, and refinement statistics for wedges 13–18.

Values in parentheses are for the outer shell.

	**Wedge 13**	**Wedge 14**	**Wedge 15**	**Wedge 16**	**Wedge 17**	**Wedge 18**
**X-ray diffraction**						
φ range	65–245°	70–250°	75–255°	80–260°	85–265°	90–270°
Dose (MGy)	5.42	5.43	5.42	5.45	5.44	5.49
Wavelength (Å)	1	1	1	1	1	1
Resolution range (Å)	44.55–1.10 (1.11–1.10)	44.56–1.10 (1.11–1.10)	44.57–1.10 (1.11–1.10)	44.58–1.10 (1.11–1.10)	44.59–1.10 (1.11–1.10)	44.59–1.10 (1.11–1.10)
Space group	$P1\ 2_1\ 1$	$P1\ 2_1\ 1$	$P1\ 2_1\ 1$	$P1\ 2_1\ 1$	$P1\ 2_1\ 1$	$P1\ 2_1\ 1$
a, *b*, *c* (Å)	67.569, 42.176, 69.636	67.592, 42.155, 69.666	67.616, 42.136, 69.698	67.630, 42.120, 69.721	67.643, 42.116, 69.736	67.640, 42.116, 69.734
α, *β*, *γ* (°)	90, 98.99, 90	90, 99.00, 90	90, 99.01, 90	90, 99.02, 90	90, 99.02, 90	90, 99.01, 90
Total reflections	546843 (20240)	547781 (20192)	548859 (19483)	549493 (19500)	547588 (19524)	545304 (20255)
Unique reflections	290886 (10377)	291118 (10342)	291134 (9974)	290968 (9963)	290550 (9957)	289810 (10318)
Multiplicity	3.6 (3.8)	3.6 (3.8)	3.6 (3.8)	3.6 (3.8)	3.6 (3.8)	3.6 (3.8)
Completeness (%)	94.30 (90.32)	94.36 (90.35)	94.34 (90.38)	94.26 (90.29)	94.09 (90.11)	93.86 (90.00)
Average I/σ(I)	5.50 (0.81)	5.34 (0.75)	5.49 (0.76)	5.40 (0.70)	5.30 (0.69)	5.39 (0.71)
Wilson B-factor	9.98	10.2	10.19	10.37	10.36	10.31
R_{merge}	0.106 (0.2997)	0.1061 (0.3046)	0.1025 (0.2996)	0.1019 (0.2957)	0.1045 (0.3109)	0.105 (0.3134)
R_{meas}	0.1306 (0.3489)	0.1303 (0.3543)	0.1257 (0.3485)	0.125 (0.344)	0.1285 (0.3618)	0.1295 (0.3644)
R_{pim}	0.07493 (0.1776)	0.07443 (0.18)	0.07148 (0.1772)	0.07124 (0.1748)	0.07358 (0.184)	0.07452 (0.185)
$CC_{1/2}$	0.965 (0.897)	0.967 (0.89)	0.977 (0.899)	0.971 (0.909)	0.972 (0.899)	0.969 (0.903)
CC*	0.991 (0.972)	0.992 (0.97)	0.994 (0.973)	0.993 (0.976)	0.993 (0.973)	0.992 (0.974)
**Refinement**						
Working set reflections	290886 (10377)	291118 (10342)	291134 (9974)	290968 (9963)	290550 (9957)	289810 (10318)
Test set reflections	3845 (152)	3849 (143)	3858 (137)	3871 (132)	3858 (129)	3806 (135)
R_{work}	0.1659 (0.2938)	0.1666 (0.3025)	0.1608 (0.3036)	0.1614 (0.3112)	0.1682 (0.3166)	0.1705 (0.3320)
R_{free}	0.1900 (0.3035)	0.1887 (0.3345)	0.1845 (0.3133)	0.1851 (0.2890)	0.2028 (0.3435)	0.1983 (0.3362)
No. of non-H atoms	5101	5101	5101	5101	5101	5101
Macromolecules	3860	3860	3860	3860	3860	3860
Ligands	131	131	131	131	131	131
Solvent	1110	1110	1110	1110	1110	1110
Protein residues	446	446	446	446	446	446
RMSD - Bonds (Å)	0.005	0.004	0.01	0.008	0.005	0.005
RMSD - Angles (°)	0.94	0.86	1.21	1.06	0.93	0.91
Ramachandran Favored (%)	96.15	96.15	96.15	96.38	96.38	96.15
Ramachandran Allowed (%)	3.39	3.39	3.62	3.39	3.39	3.39
Ramachandran Outliers (%)	0.45	0.45	0.23	0.23	0.23	0.45
Rotamer outliers (%)	1.85	1.39	1.62	1.62	1.62	1.62
Clashscore	2.66	2.03	3.92	4.05	3.67	2.28
Average B-factor	16.57	15.89	16.49	16.33	15.97	16.84
Macromolecules	13.44	12.79	13.47	13.13	12.94	13.79
Ligands	29.1	27.51	27.16	29.12	26.98	27.57
Solvent	25.98	25.3	25.72	25.98	25.24	26.16

Table S10 Data collection, processing, and refinement statistics for wedges 19–24.

Values in parentheses are for the outer shell.

	Wedge 19	Wedge 20	Wedge 21	Wedge 22	Wedge 23	Wedge 24
**X-ray diffraction**						
φ range	95–275°	100–280°	105–285°	110–290°	115–295°	120–300°
Dose (MGy)	5.46	5.49	5.46	5.50	5.50	5.50
Wavelength (Å)	1	1	1	1	1	1
Resolution range (Å)	44.58–1.10 (1.11–1.10)	44.58–1.10 (1.11–1.10)	44.57–1.10 (1.11–1.10)	44.55–1.10 (1.11–1.10)	44.54–1.10 (1.11–1.10)	44.52–1.10 (1.11–1.10)
Space group	$P1\ 2_1\ 1$	$P1\ 2_1\ 1$	$P1\ 2_1\ 1$	$P1\ 2_1\ 1$	$P1\ 2_1\ 1$	$P1\ 2_1\ 1$
a, *b*, *c* (Å)	67.626, 42.127, 69.722	67.614, 42.148, 69.708	67.599, 42.173, 69.689	67.579, 42.193, 69.657	67.558, 42.210, 69.626	67.543, 42.225, 69.599
α, *β*, *γ* (°)	90, 99.01, 90	90, 99.01, 90	90, 99.00, 90	90, 98.99, 90	90, 98.99, 90	90, 98.99, 90
Total reflections	544115 (20250)	540425 (20217)	540697 (20229)	540822 (20203)	541890 (20189)	540671 (20186)
Unique reflections	288968 (10288)	288495 (10273)	287990 (10249)	287523 (10209)	287123 (10181)	286609 (10167)
Multiplicity	3.6 (3.8)	3.6 (3.8)	3.6 (3.8)	3.6 (3.8)	3.6 (3.8)	3.6 (3.8)
Completeness (%)	93.60 (89.80)	93.43 (89.65)	93.26 (89.26)	93.14 (89.08)	93.04 (88.75)	92.90 (88.59)
Average I/σ(I)	5.37 (0.71)	5.48 (0.82)	5.58 (0.88)	5.85 (0.99)	5.78 (0.94)	5.99 (1.04)
Wilson B-factor	10.23	10.01	9.84	9.56	9.41	9.46
R_{merge}	0.1073 (0.3238)	0.1107 (0.3394)	0.1102 (0.3271)	0.1085 (0.3085)	0.1076 (0.2917)	0.1056 (0.2889)
R_{meas}	0.1328 (0.3764)	0.1371 (0.3946)	0.1365 (0.3802)	0.1342 (0.3588)	0.1328 (0.3393)	0.1306 (0.3361)
R_{pim}	0.07682 (0.1911)	0.07956 (0.2002)	0.07916 (0.1929)	0.0777 (0.1824)	0.07658 (0.1724)	0.0755 (0.1709)
$CC_{1/2}$	0.966 (0.878)	0.962 (0.869)	0.967 (0.87)	0.965 (0.874)	0.968 (0.901)	0.97 (0.891)
CC*	0.991 (0.967)	0.99 (0.964)	0.991 (0.964)	0.991 (0.966)	0.992 (0.974)	0.992 (0.971)
**Refinement**						
Working set reflections	288968 (10288)	288495 (10273)	287990 (10249)	287523 (10209)	287123 (10181)	286609 (10167)
Test set reflections	3844 (149)	3779 (136)	3797 (136)	3749 (116)	3785 (136)	3808 (144)
R_{work}	0.1727 (0.3258)	0.1750 (0.3041)	0.1701 (0.2806)	0.1697 (0.2781)	0.1694 (0.2928)	0.1715 (0.2738)
R_{free}	0.1953 (0.3827)	0.2133 (0.2971)	0.1949 (0.2990)	0.1924 (0.2765)	0.1892 (0.2928)	0.1931 (0.2809)
No. of non-H atoms	5101	5101	5101	5101	5101	5101
Macromolecules	3860	3860	3860	3860	3860	3860
Ligands	131	131	131	131	131	131
Solvent	1110	1110	1110	1110	1110	1110
Protein residues	446	446	446	446	446	446
RMSD - Bonds (Å)	0.004	0.006	0.005	0.005	0.005	0.003
RMSD - Angles (°)	0.82	1.01	0.92	0.94	0.89	0.8
Ramachandran Favored (%)	96.15	96.38	96.15	95.93	95.93	95.93
Ramachandran Allowed (%)	3.62	3.39	3.62	3.62	3.62	3.62
Ramachandran Outliers (%)	0.23	0.23	0.23	0.45	0.45	0.45
Rotamer outliers (%)	1.62	1.85	1.85	2.08	1.85	1.39
Clashscore	1.39	3.43	3.03	2.53	2.4	1.52
Average B-factor	16.68	16.53	16.64	15.97	15.71	15.82
Macromolecules	13.59	13.38	13.5	12.86	12.6	12.68
Ligands	28.76	27.33	29.87	27.61	27.38	27.94
Solvent	25.99	26.21	25.96	25.41	25.17	25.28

Table S11 Data collection, processing, and refinement statistics for wedges 25–30.

Values in parentheses are for the outer shell.

	Wedge 25	Wedge 26	Wedge 27	Wedge 28	Wedge 29	Wedge 30
**X-ray diffraction**						
φ range	125–305°	130–310°	135–315°	140–320°	145–325°	150–330°
Dose (MGy)	5.51	5.48	5.48	5.43	5.43	5.41
Wavelength (Å)	1	1	1	1	1	1
Resolution range (Å)	44.51–1.10 (1.11–1.10)	44.50–1.10 (1.11–1.10)	44.49–1.10 (1.11–1.10)	44.49–1.10 (1.11–1.10)	44.48–1.10 (1.11–1.10)	44.48–1.10 (1.11–1.10)
Space group	$P1\ 2_1\ 1$	$P1\ 2_1\ 1$	$P1\ 2_1\ 1$	$P1\ 2_1\ 1$	$P1\ 2_1\ 1$	$P1\ 2_1\ 1$
a, *b*, *c* (Å)	67.536, 42.239, 69.575	67.525, 42.248, 69.549	67.519, 42.255, 69.529	67.512, 42.262, 69.518	67.510, 42.269, 69.514	67.511, 42.269, 69.516
α, *β*, *γ* (°)	90, 98.99, 90	90, 98.99, 90	90, 98.99, 90	90, 98.99, 90	90, 98.99, 90	90, 99.00, 90
Total reflections	540764 (20185)	539594 (20206)	540835 (20250)	540709 (20262)	541453 (20300)	540643 (20302)
Unique reflections	286148 (10142)	285633 (10130)	285362 (10137)	284966 (10140)	285191 (10171)	285197 (10181)
Multiplicity	3.6 (3.8)	3.6 (3.8)	3.6 (3.8)	3.6 (3.8)	3.6 (3.8)	3.6 (3.8)
Completeness (%)	92.76 (88.43)	92.62 (88.25)	92.55 (88.22)	92.42 (88.03)	92.48 (88.08)	92.48 (88.15)
Average I/σ(I)	5.96 (1.07)	5.85 (1.05)	5.70 (1.03)	5.59 (0.99)	5.70 (1.01)	5.61 (0.98)
Wilson B-factor	9.45	9.37	9.29	9.37	9.39	9.51
R_{merge}	0.1072 (0.2829)	0.1088 (0.2825)	0.109 (0.2805)	0.1116 (0.2931)	0.1098 (0.287)	0.1116 (0.2978)
R_{meas}	0.1325 (0.329)	0.1349 (0.3288)	0.135 (0.3262)	0.1388 (0.341)	0.1365 (0.3339)	0.1391 (0.3464)
R_{pim}	0.0766 (0.1672)	0.07832 (0.1672)	0.07824 (0.1657)	0.081 (0.1734)	0.07966 (0.1698)	0.08161 (0.176)
$CC_{1/2}$	0.966 (0.893)	0.965 (0.872)	0.97 (0.89)	0.964 (0.89)	0.965 (0.887)	0.963 (0.879)
CC*	0.991 (0.971)	0.991 (0.965)	0.992 (0.971)	0.991 (0.97)	0.991 (0.97)	0.99 (0.967)
**Refinement**						
Working set reflections	286148 (10142)	285633 (10130)	285362 (10137)	284966 (10140)	285191 (10171)	285197 (10181)
Test set reflections	3778 (124)	3763 (129)	3765 (132)	3733 (132)	3753 (135)	3786 (131)
R_{work}	0.1694 (0.2697)	0.1694 (0.2721)	0.1708 (0.2717)	0.1698 (0.2753)	0.1696 (0.2635)	0.1721 (0.2625)
R_{free}	0.1882 (0.3334)	0.1903 (0.2682)	0.1934 (0.3260)	0.1938 (0.3146)	0.1956 (0.3131)	0.1927 (0.2803)
No. of non-H atoms	5101	5101	5101	5101	5101	5101
Macromolecules	3860	3860	3860	3860	3860	3860
Ligands	131	131	131	131	131	131
Solvent	1110	1110	1110	1110	1110	1110
Protein residues	446	446	446	446	446	446
RMSD - Bonds (Å)	0.004	0.004	0.003	0.007	0.005	0.011
RMSD - Angles (°)	0.83	0.88	0.81	1.02	0.92	1.2
Ramachandran Favored (%)	96.15	95.93	96.15	95.93	96.38	97.06
Ramachandran Allowed (%)	3.39	3.62	3.39	3.85	3.39	2.71
Ramachandran Outliers (%)	0.45	0.45	0.45	0.23	0.23	0.23
Rotamer outliers (%)	1.39	1.39	1.39	1.39	1.62	1.62
Clashscore	1.39	3.29	1.9	3.41	2.28	3.29
Average B-factor	15.85	15.62	15.57	15.6	15.94	15.83
Macromolecules	12.75	12.5	12.48	12.56	12.76	12.67
Ligands	28.38	28.3	27.84	26.84	29.86	28.67
Solvent	25.14	24.98	24.88	24.87	25.34	25.3

Table S12 Data collection, processing, and refinement statistics for wedges 31–36.

Values in parentheses are for the outer shell.

	**Wedge 31**	**Wedge 32**	**Wedge 33**	**Wedge 34**	**Wedge 35**	**Wedge 36**
**X-ray diffraction**						
φ range	155–335°	160–340°	165–345°	170–350°	175–355°	180–360°
Dose (MGy)	5.41	5.42	5.42	5.44	5.43	5.48
Wavelength (Å)	1	1	1	1	1	1
Resolution range (Å)	44.49–1.10 (1.11–1.10)	34.35–1.10 (1.11–1.10)	44.51–1.10 (1.11–1.10)	44.52–1.10 (1.11–1.10)	44.54–1.10 (1.11–1.10)	44.55–1.10 (1.11–1.10)
Space group	$P1\ 2_1\ 1$	$P1\ 2_1\ 1$	$P1\ 2_1\ 1$	$P1\ 2_1\ 1$	$P1\ 2_1\ 1$	$P1\ 2_1\ 1$
a, *b*, *c* (Å)	67.507, 42.257, 69.525	67.513, 42.245, 69.552	67.525, 42.227, 69.584	67.527, 42.206, 69.613	67.526, 42.190, 69.641	67.519, 42.180, 69.662
α, *β*, *γ* (°)	90, 99.00, 90	90, 99.00, 90	90, 99.00, 90	90, 98.99, 90	90, 98.98, 90	90, 98.97, 90
Total reflections	538491 (20225)	537787 (20215)	539062 (20219)	538466 (20207)	538392 (20229)	539759 (20293)
Unique reflections	285225 (10158)	285481 (10172)	286153 (10190)	286441 (10206)	286786 (10252)	287462 (10315)
Multiplicity	3.6 (3.8)	3.6 (3.8)	3.6 (3.8)	3.6 (3.8)	3.6 (3.8)	3.6 (3.8)
Completeness (%)	92.52 (88.44)	92.59 (88.50)	92.79 (88.70)	92.90 (88.97)	93.00 (89.33)	93.21 (89.53)
Average I/σ(I)	5.57 (1.03)	5.49 (0.98)	5.35 (0.96)	5.24 (0.91)	5.11 (0.89)	5.19 (0.92)
Wilson B-factor	9.4	9.46	9.51	9.59	9.56	9.53
R_{merge}	0.116 (0.3026)	0.1143 (0.306)	0.1184 (0.3086)	0.1198 (0.3189)	0.1217 (0.3176)	0.1208 (0.3102)
R_{meas}	0.1446 (0.352)	0.1427 (0.3559)	0.1474 (0.3588)	0.1496 (0.371)	0.1522 (0.3694)	0.1502 (0.3607)
R_{pim}	0.08477 (0.1789)	0.08387 (0.1809)	0.08638 (0.1821)	0.08804 (0.1887)	0.08972 (0.1877)	0.08784 (0.1832)
$CC_{1/2}$	0.959 (0.87)	0.965 (0.859)	0.96 (0.87)	0.951 (0.852)	0.954 (0.875)	0.96 (0.864)
CC*	0.989 (0.965)	0.991 (0.961)	0.99 (0.965)	0.987 (0.959)	0.988 (0.966)	0.99 (0.963)
**Refinement**						
Working set reflections	285225 (10158)	285481 (10172)	286153 (10190)	286441 (10206)	286786 (10252)	287462 (10315)
Test set reflections	3755 (135)	3772 (125)	3814 (136)	3774 (130)	3800 (130)	3781 (133)
R_{work}	0.1775 (0.2638)	0.1840 (0.2683)	0.1849 (0.2758)	0.1849 (0.2781)	0.1849 (0.2703)	0.1803 (0.2701)
R_{free}	0.2059 (0.3368)	0.1996 (0.2688)	0.2141 (0.3066)	0.2060 (0.2690)	0.2133 (0.2994)	0.2027 (0.2865)
No. of non-H atoms	5101	5101	5101	5101	5101	5101
Macromolecules	3860	3860	3860	3860	3860	3860
Ligands	131	131	131	131	131	131
Solvent	1110	1110	1110	1110	1110	1110
Protein residues	446	446	446	446	446	446
RMSD - Bonds (Å)	0.006	0.003	0.003	0.004	0.005	0.004
RMSD - Angles (°)	0.99	0.76	0.77	0.87	0.91	0.84
Ramachandran Favored (%)	95.93	95.93	95.7	96.38	96.15	95.7
Ramachandran Allowed (%)	3.85	3.62	3.85	3.17	3.39	3.85
Ramachandran Outliers (%)	0.23	0.45	0.45	0.45	0.45	0.45
Rotamer outliers (%)	1.62	1.39	1.39	1.39	1.39	1.39
Clashscore	3.55	1.27	1.9	2.02	3.03	0.89
Average B-factor	15.64	15.72	16.05	15.74	15.66	15.97
Macromolecules	12.59	12.63	12.91	12.61	12.66	12.89
Ligands	27.14	27.86	29.59	27.72	26.59	26.85
Solvent	24.92	25.04	25.35	25.21	24.8	25.42

Table S13 Occupancy multiple regression results.

Atom	Estimate	Coefficient	Standard Error	*p*-Value
**Dioxygen**	Chain A Trend	-0.00889	0.00310	0.00699
	Chain B Trend	0.00092	0.00930	0.92179
	Contrast	-0.00980	0.00980	0.32286
**Intact Glu30**	Chain A Trend	N/A	N/A	N/A
	Chain B Trend	-0.01335	0.00330	0.00028
	Contrast	N/A	N/A	N/A
**CO_2**	Chain A Trend	0.01693	0.00674	0.01699
	Chain B Trend	N/A	N/A	N/A
	Contrast	N/A	N/A	N/A
**H_2O_{ax}**	Chain A Trend	-0.00953	0.00286	0.00206
	Chain B Trend	-0.00418	0.00370	0.26618
	Contrast	-0.00535	0.00467	0.25613
**H_2O_{eq}**	Chain A Trend	-0.01128	0.00376	0.00501
	Chain B Trend	-0.01036	0.00448	0.02710
	Contrast	-0.00093	0.00585	0.87483

Table S14 Distance multiple regression results.

Atom Pair	Estimate	Coefficient	Standard Error	*p*-Value
**Cu–Tyr168Oη**	Chain A Trend	0.00126	0.00074	0.09585
	Chain B Trend	0.00084	0.00101	0.41094
	Contrast	0.00042	0.00125	0.73832
**Cu–N_{term}**	Chain A Trend	0.00171	0.00124	0.17586
	Chain B Trend	-0.00004	0.00144	0.97778
	Contrast	0.00175	0.00190	0.35898
**Cu–His1Nδ**	Chain A Trend	-0.00179	0.00105	0.09917
	Chain B Trend	0.00093	0.00110	0.40685
	Contrast	-0.00271	0.00153	0.08001
**Cu–His84Nε**	Chain A Trend	-0.00192	0.00105	0.07565
	Chain B Trend	-0.00212	0.00093	0.02814
	Contrast	0.00020	0.00140	0.88747
**Cu–H_2O_{eq}**	Chain A Trend	0.00303	0.00229	0.19547
	Chain B Trend	-0.00300	0.00334	0.37496
	Contrast	0.00603	0.00405	0.14183
**Cu–H_2O_{ax}**	Chain A Trend	-0.00193	0.00240	0.42548
	Chain B Trend	0.00502	0.00326	0.13288
	Contrast	-0.00695	0.00405	0.09070

Table S15 Angle multiple regression results.

Angle	Estimate	Coefficient	Standard Error	*p*-Value
**θ_1**	Chain A Trend	0.10476	0.03071	0.00169
	Chain B Trend	0.09028	0.04860	0.07189
	Contrast	0.01448	0.05749	0.80203
**θ_2**	Chain A Trend	0.09244	0.04773	0.06117
	Chain B Trend	0.21800	0.05587	0.00043
	Contrast	-0.12557	0.07348	0.09215
**θ_3**	Chain A Trend	-0.14086	0.05853	0.02678
	Chain B Trend	-0.33325	0.05483	0.00001
	Contrast	0.19240	0.08020	0.01924
**θ_T**	Chain A Trend	-0.01002	0.04287	0.81664
	Chain B Trend	0.14406	0.05328	0.01065
	Contrast	-0.15407	0.06839	0.02764
**θ_{H-H}**	Chain A Trend	-0.12233	0.07888	0.13029
	Chain B Trend	-0.13338	0.09996	0.19110
	Contrast	0.01105	0.12733	0.93109
**θ_{H1}**	Chain A Trend	0.16322	0.08671	0.06829
	Chain B Trend	0.33794	0.08103	0.00020
	Contrast	-0.17472	0.11868	0.14562
**θ_{HN}**	Chain A Trend	0.17838	0.05904	0.00474
	Chain B Trend	0.24125	0.08723	0.00914
	Contrast	-0.06287	0.10534	0.55288

Table S16 Measured occupancies for all structures.

Pseudohelix Number	Average DDWD (MGy)	Chain	Residue				
			CO_2	H_2O_{ax}	H_2O_{eq}	Dioxygen	Intact Glu_{30}
1	1.22	A	0.22	0.52	0.73	0.52	N/A
2	1.74	A	0.42	0.41	0.59	0.44	N/A
3	2.07	A	0.28	0.41	0.59	0.4	N/A
4	2.37	A	0.4	0.38	0.55	0.41	N/A
5	2.74	A	0.32	0.43	0.55	0.43	N/A
6	2.98	A	0.36	0.41	0.52	0.46	N/A
7	3.39	A	0.33	0.41	0.57	0.4	N/A
8	3.56	A	0.37	0.41	0.6	0.44	N/A
9	3.81	A	0.47	0.42	0.56	0.43	N/A
10	3.94	A	0.42	0.42	0.57	0.4	N/A
11	4.18	A	0.29	0.41	0.55	0.41	N/A
12	4.44	A	0.28	0.41	0.58	0.41	N/A
13	4.9	A	0.38	0.46	0.6	0.4	N/A
14	5.12	A	0.47	0.41	0.61	0.41	N/A
15	5.27	A	0.37	0.46	0.6	0.4	N/A
16	5.33	A	0.43	0.45	0.64	0.38	N/A
17	5.56	A	0.4	0.4	0.61	0.45	N/A
18	5.7	A	0.31	0.42	0.61	0.37	N/A
19	5.91	A	0.47	0.43	0.57	0.36	N/A
20	5.82	A	0.32	0.42	0.6	0.36	N/A
21	5.81	A	0.45	0.43	0.57	0.46	N/A
22	5.6	A	0.36	0.43	0.54	0.43	N/A
23	5.51	A	0.37	0.39	0.6	0.41	N/A
24	5.35	A	0.45	0.43	0.62	0.43	N/A
25	5.33	A	0.44	0.38	0.57	0.44	N/A
26	5.51	A	0.49	0.39	0.55	0.43	N/A
27	5.55	A	0.41	0.39	0.57	0.35	N/A
28	6.26	A	0.44	0.39	0.57	0.32	N/A
29	6.41	A	0.33	0.36	0.57	0.39	N/A
30	6.53	A	0.45	0.41	0.49	0.42	N/A
31	6.66	A	0.46	0.41	0.53	0.38	N/A
32	6.99	A	0.33	0.42	0.55	0.41	N/A
33	7.54	A	0.37	0.41	0.56	0.39	N/A
34	7.82	A	0.63	0.36	0.55	0.41	N/A
35	8.13	A	0.37	0.37	0.49	0.42	N/A
36	8.65	A	0.38	0.31	0.5	0.39	N/A
1	1.22	B	N/A	0.55	0.72	0.45	0.59
2	1.74	B	N/A	0.4	0.55	0.5	0.52
3	2.07	B	N/A	0.37	0.53	0.45	0.45
4	2.37	B	N/A	0.36	0.53	0.67	0.45
5	2.74	B	N/A	0.37	0.55	0.54	0.47
6	2.98	B	N/A	0.38	0.52	0.6	0.45
7	3.39	B	N/A	0.38	0.49	0.65	0.45
8	3.56	B	N/A	0.37	0.54	0.65	0.43
9	3.81	B	N/A	0.38	0.47	0.54	0.46
10	3.94	B	N/A	0.38	0.47	0.73	0.43
11	4.18	B	N/A	0.41	0.51	0.61	0.44

12	4.44	B	N/A	0.38	0.53	0.48	0.48
13	4.9	B	N/A	0.37	0.49	0.59	0.45
14	5.12	B	N/A	0.41	0.44	0.62	0.41
15	5.27	B	N/A	0.36	0.46	0.68	0.42
16	5.33	B	N/A	0.4	0.5	0.66	0.38
17	5.56	B	N/A	0.4	0.5	0.66	0.4
18	5.7	B	N/A	0.4	0.46	0.71	0.45
19	5.91	B	N/A	0.42	0.52	0.68	0.42
20	5.82	B	N/A	0.41	0.5	0.61	0.46
21	5.81	B	N/A	0.44	0.55	0.6	0.43
22	5.6	B	N/A	0.4	0.5	0.59	0.44
23	5.51	B	N/A	0.37	0.52	0.42	0.38
24	5.35	B	N/A	0.36	0.47	0.71	0.44
25	5.33	B	N/A	0.39	0.5	0.61	0.41
26	5.51	B	N/A	0.37	0.53	0.53	0.47
27	5.55	B	N/A	0.34	0.5	0.51	0.48
28	6.26	B	N/A	0.35	0.51	0.6	0.41
29	6.41	B	N/A	0.4	0.59	0.63	0.42
30	6.53	B	N/A	0.39	0.55	0.37	0.44
31	6.66	B	N/A	0.43	0.54	0.55	0.45
32	6.99	B	N/A	0.34	0.55	0.72	0.47
33	7.54	B	N/A	0.36	0.54	0.33	0.47
34	7.82	B	N/A	0.46	0.52	0.55	0.44
35	8.13	B	N/A	0.4	0.49	0.57	0.37
36	8.65	B	N/A	0.33	0.41	0.57	0.36

Table S17 Measured angles for all structures.

Pseudohelix Number	Average DDWD (MGy)	Chain	Angle (°)						
			θ_1	θ_2	θ_3	θ_T	θ_{H-H}	θ_{H1}	θ_{HN}
1	1.22	A	92.20	92.76	172.95	5.01	69.70	-3.09	8.03
2	1.74	A	92.40	94.70	170.49	6.31	67.89	-0.97	8.78
3	2.07	A	92.85	93.85	170.11	7.27	66.62	1.04	8.10
4	2.37	A	92.76	94.34	170.04	6.98	66.94	1.12	8.09
5	2.74	A	93.12	94.02	170.71	5.93	67.76	0.64	8.93
6	2.98	A	93.25	93.64	170.24	6.90	66.57	1.27	8.02
7	3.39	A	92.63	94.68	170.51	6.04	68.20	0.13	9.13
8	3.56	A	92.61	93.87	171.09	6.12	66.95	-0.08	8.72
9	3.81	A	92.98	94.32	170.15	6.60	67.06	0.52	8.93
10	3.94	A	92.95	94.70	170.24	6.03	67.10	0.42	9.91
11	4.18	A	92.93	93.73	170.69	6.50	67.47	0.65	8.65
12	4.44	A	93.21	94.88	169.58	6.56	67.55	1.28	10.19
13	4.9	A	92.14	94.88	170.68	6.11	67.05	-0.58	9.29
14	5.12	A	92.89	93.71	171.20	5.80	66.50	0.07	8.83
15	5.27	A	93.01	93.85	170.77	6.17	67.20	-0.57	8.69
16	5.33	A	93.18	94.23	170.18	6.42	68.03	0.53	8.73
17	5.56	A	93.12	93.55	171.27	5.63	67.90	-0.92	8.92
18	5.7	A	93.21	94.74	169.95	6.13	68.40	0.53	9.89
19	5.91	A	93.08	93.80	170.98	5.82	67.74	-0.55	9.31
20	5.82	A	92.79	94.29	170.74	5.96	67.37	-0.99	9.61
21	5.81	A	93.57	93.48	170.66	6.12	67.97	0.65	8.33
22	5.6	A	93.46	94.82	169.88	5.82	67.35	0.37	9.35
23	5.51	A	93.10	94.81	170.07	5.99	67.95	0.00	10.32
24	5.35	A	93.49	94.14	169.98	6.48	66.25	0.25	7.90
25	5.33	A	93.16	94.22	170.32	6.25	64.56	0.89	8.71
26	5.51	A	92.81	94.73	169.94	6.65	66.49	0.94	8.97
27	5.55	A	92.90	94.70	170.27	6.06	66.46	1.31	10.19
28	6.26	A	94.02	94.13	169.87	6.02	66.87	0.19	9.30
29	6.41	A	93.23	94.11	170.56	5.93	66.30	0.71	9.94
30	6.53	A	93.19	93.76	170.36	6.67	66.32	0.57	8.49
31	6.66	A	92.97	93.93	170.69	6.24	66.85	0.85	8.18
32	6.99	A	93.58	94.33	169.78	6.46	67.00	1.02	9.53
33	7.54	A	92.95	94.52	170.62	5.66	66.50	-0.72	9.72
34	7.82	A	92.98	94.13	170.54	6.23	67.54	0.61	9.07
35	8.13	A	93.01	94.64	170.27	6.00	66.91	0.40	9.30
36	8.65	A	93.41	95.41	168.65	7.13	67.61	2.58	10.24
1	1.22	B	93.72	90.62	173.87	4.33	70.17	-0.26	6.86
2	1.74	B	93.95	92.02	172.20	5.02	68.55	0.73	7.38
3	2.07	B	94.15	91.98	172.42	4.47	69.76	1.57	8.18
4	2.37	B	93.31	92.45	172.91	4.13	70.36	1.55	9.10
5	2.74	B	94.03	92.54	172.09	4.40	69.01	0.83	7.92
6	2.98	B	93.45	92.74	171.84	5.31	68.57	1.98	7.34
7	3.39	B	94.21	92.96	171.31	4.91	68.22	3.13	8.25
8	3.56	B	93.88	92.13	172.16	5.05	68.78	2.04	7.88
9	3.81	B	94.48	91.69	172.07	4.98	68.77	2.43	8.30
10	3.94	B	94.73	91.38	172.15	4.92	67.04	2.54	7.80
11	4.18	B	94.04	93.03	171.89	3.95	69.27	1.34	10.45

12	4.44	B	94.69	92.10	171.35	5.37	68.11	2.63	8.07
13	4.9	B	94.20	92.05	171.68	5.49	68.07	2.93	7.75
14	5.12	B	94.15	91.42	171.76	6.07	68.73	3.43	7.02
15	5.27	B	94.07	91.43	172.19	5.55	68.71	1.92	7.04
16	5.33	B	93.16	93.49	171.38	5.49	71.72	1.99	8.39
17	5.56	B	93.40	93.52	171.10	5.59	69.64	1.96	8.93
18	5.7	B	94.42	93.22	170.86	5.02	69.33	1.39	8.61
19	5.91	B	94.65	92.39	170.88	5.80	69.58	3.19	8.58
20	5.82	B	94.14	92.50	171.44	5.40	68.43	1.93	9.19
21	5.81	B	93.92	92.56	172.41	3.97	68.97	0.67	9.65
22	5.6	B	93.92	92.18	172.78	3.86	68.66	1.18	9.78
23	5.51	B	93.62	93.02	172.29	3.92	68.53	0.59	10.15
24	5.35	B	93.68	93.43	171.64	4.38	69.97	1.59	10.25
25	5.33	B	92.55	92.66	172.54	5.33	68.69	2.52	7.47
26	5.51	B	93.34	92.84	171.57	5.74	69.67	2.13	7.51
27	5.55	B	93.68	92.98	171.76	4.85	69.19	1.84	9.46
28	6.26	B	94.87	93.17	170.54	4.98	67.27	1.89	9.15
29	6.41	B	94.31	92.58	171.69	4.66	68.66	2.19	9.60
30	6.53	B	93.68	92.06	172.06	5.49	69.73	2.12	7.48
31	6.66	B	93.95	92.52	171.86	4.93	71.30	2.70	9.99
32	6.99	B	93.96	92.91	171.14	5.59	69.50	2.34	8.74
33	7.54	B	94.62	92.79	170.91	5.27	67.73	2.76	9.55
34	7.82	B	94.43	93.23	170.35	5.87	67.00	4.99	9.27
35	8.13	B	94.54	93.65	169.96	5.81	66.92	4.38	8.14
36	8.65	B	95.14	93.53	169.76	5.43	68.69	3.38	9.26

Table S18 Measured distances for all structures.

Pseudohelix Number	Average DDWD (MGy)	Chain	Distance (Cu–X, Å)					
			H_2O_{eq}	H_2O_{ax}	Tyr168Oη	N_{term}	His1Nδ	His84Nε
1	1.22	A	1.98	2.35	2.63	2.11	1.96	2.02
2	1.74	A	2.00	2.33	2.64	2.13	1.96	1.99
3	2.07	A	1.97	2.36	2.63	2.15	1.96	1.97
4	2.37	A	1.98	2.38	2.63	2.16	1.96	1.99
5	2.74	A	1.98	2.38	2.64	2.15	1.96	1.97
6	2.98	A	2.02	2.41	2.64	2.14	1.95	1.99
7	3.39	A	1.99	2.39	2.64	2.15	1.95	1.98
8	3.56	A	2.01	2.38	2.64	2.15	1.95	1.98
9	3.81	A	1.97	2.35	2.64	2.16	1.95	1.99
10	3.94	A	1.97	2.35	2.62	2.15	1.95	1.98
11	4.18	A	1.98	2.37	2.63	2.16	1.96	1.99
12	4.44	A	2.04	2.30	2.64	2.15	1.96	1.97
13	4.9	A	1.95	2.37	2.63	2.15	1.97	1.98
14	5.12	A	2.02	2.36	2.62	2.16	1.95	1.98
15	5.27	A	2.02	2.34	2.63	2.16	1.96	1.99
16	5.33	A	1.96	2.35	2.65	2.14	1.95	1.97
17	5.56	A	1.99	2.35	2.64	2.12	1.95	1.98
18	5.7	A	1.97	2.33	2.64	2.16	1.97	1.98
19	5.91	A	1.98	2.35	2.64	2.15	1.98	1.97
20	5.82	A	1.96	2.33	2.64	2.16	1.95	1.98
21	5.81	A	2.04	2.38	2.65	2.17	1.95	2.00
22	5.6	A	1.98	2.35	2.65	2.15	1.95	1.97
23	5.51	A	2.02	2.35	2.62	2.15	1.97	2.00
24	5.35	A	2.03	2.33	2.65	2.15	1.97	1.99
25	5.33	A	2.00	2.37	2.65	2.15	1.96	1.99
26	5.51	A	1.97	2.30	2.63	2.16	1.98	1.99
27	5.55	A	1.99	2.37	2.64	2.14	1.99	1.98
28	6.26	A	2.03	2.33	2.64	2.14	1.97	1.99
29	6.41	A	1.99	2.32	2.64	2.15	1.94	1.99
30	6.53	A	2.01	2.38	2.64	2.14	1.96	1.97
31	6.66	A	2.00	2.34	2.64	2.14	1.94	1.98
32	6.99	A	1.98	2.39	2.63	2.14	1.94	1.99
33	7.54	A	2.02	2.34	2.63	2.15	1.95	2.00
34	7.82	A	2.04	2.33	2.64	2.14	1.95	1.97
35	8.13	A	2.02	2.38	2.65	2.16	1.94	1.97
36	8.65	A	1.98	2.37	2.65	2.18	1.94	1.98
1	1.22	B	2.03	2.43	2.66	2.16	1.91	2.02
2	1.74	B	2.00	2.41	2.67	2.19	1.91	2.01
3	2.07	B	1.99	2.43	2.68	2.21	1.91	2.01
4	2.37	B	2.01	2.38	2.68	2.20	1.92	2.01
5	2.74	B	1.99	2.41	2.69	2.21	1.92	2.00
6	2.98	B	2.01	2.40	2.68	2.19	1.93	2.00
7	3.39	B	1.99	2.44	2.69	2.18	1.94	2.00
8	3.56	B	2.03	2.41	2.68	2.22	1.94	2.01
9	3.81	B	2.01	2.42	2.69	2.20	1.93	2.01
10	3.94	B	2.04	2.41	2.68	2.18	1.93	2.01
11	4.18	B	2.06	2.36	2.67	2.20	1.96	2.01

12	4.44	B	2.03	2.35	2.69	2.18	1.95	2.01
13	4.9	B	1.95	2.38	2.69	2.17	1.93	2.01
14	5.12	B	2.05	2.38	2.67	2.19	1.93	2.00
15	5.27	B	2.04	2.39	2.67	2.20	1.94	1.99
16	5.33	B	2.04	2.35	2.70	2.15	1.91	1.98
17	5.56	B	2.02	2.39	2.69	2.18	1.95	2.00
18	5.7	B	2.05	2.39	2.68	2.17	1.94	2.01
19	5.91	B	2.00	2.42	2.70	2.19	1.94	2.00
20	5.82	B	1.97	2.37	2.69	2.17	1.93	2.00
21	5.81	B	2.01	2.44	2.67	2.18	1.93	2.00
22	5.6	B	2.09	2.44	2.68	2.18	1.94	1.99
23	5.51	B	2.03	2.43	2.68	2.19	1.94	2.01
24	5.35	B	2.06	2.45	2.68	2.17	1.93	1.99
25	5.33	B	2.02	2.43	2.69	2.17	1.93	1.99
26	5.51	B	2.07	2.45	2.69	2.17	1.94	1.99
27	5.55	B	1.97	2.45	2.68	2.17	1.93	2.00
28	6.26	B	2.02	2.42	2.69	2.19	1.94	2.01
29	6.41	B	1.95	2.39	2.67	2.19	1.92	2.01
30	6.53	B	2.00	2.44	2.66	2.21	1.90	2.00
31	6.66	B	1.94	2.45	2.68	2.19	1.93	2.00
32	6.99	B	2.00	2.48	2.67	2.21	1.93	2.00
33	7.54	B	2.02	2.39	2.67	2.18	1.93	2.02
34	7.82	B	2.01	2.50	2.70	2.19	1.91	2.01
35	8.13	B	1.98	2.38	2.67	2.20	1.93	1.98
36	8.65	B	1.96	2.47	2.70	2.21	1.93	1.99
